# π-Extension of heterocycles *via* a Pd-catalyzed heterocyclic aryne annulation: π-extended donors for TADF emitters†

**DOI:** 10.1039/d2sc01788a

**Published:** 2022-05-04

**Authors:** Katie A. Spence, Jason V. Chari, Mattia Di Niro, Robert B. Susick, Narcisse Ukwitegetse, Peter I. Djurovich, Mark E. Thompson, Neil K. Garg

**Affiliations:** Department of Chemistry and Biochemistry, University of California at Los Angeles Los Angeles California 90095 USA neilgarg@chem.ucla.edu; Department of Chemistry, University of Southern California Los Angeles California 90089 USA met@usc.edu

## Abstract

We report the annulation of heterocyclic building blocks to access π-extended polycyclic aromatic hydrocarbons (PAHs). The method involves the trapping of short-lived hetarynes with catalytically-generated biaryl palladium intermediates and allows for the concise appendage of three or more fused aromatic rings about a central heterocyclic building block. Our studies focus on annulating the indole and carbazole heterocycles through the use of indolyne and carbazolyne chemistry, respectively, the latter of which required the synthesis of a new carbazolyne precursor. Notably, these represent rare examples of transition metal-catalyzed reactions of N-containing hetarynes. We demonstrate the utility of our methodology in the synthesis of heterocyclic π-extended PAHs, which were then applied as ligands in two-coordinate metal complexes. As a result of these studies, we identified a new thermally-activated delayed fluorescence (TADF) emitter that displays up to 81% photoluminescence efficiency, along with insight into structure–property relationships. These studies underscore the utility of heterocyclic strained intermediates in the synthesis and study of organic materials.

## Abstract 2

A method for accessing π-extended heterocycles through a palladium-catalyzed annulation of strained indolyne and carbazolyne intermediates is disclosed. We demonstrate the use of these products as donor ligands in TADF emitters.

## Graphical abstract

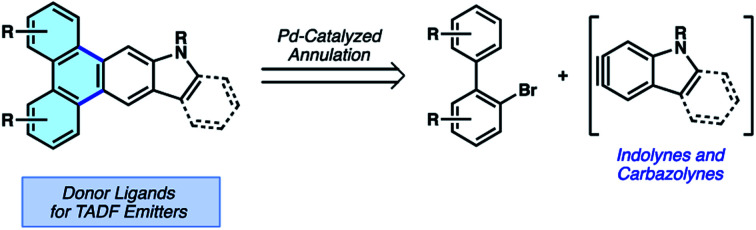

## Introduction

**Fig. 1:**
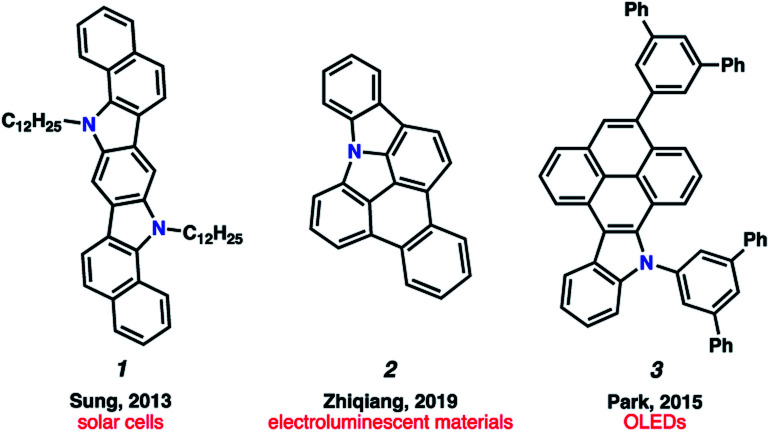
Examples of heteroatom-containing PAHs with applications in materials chemistry.

Polycyclic aromatic hydrocarbons (PAHs) have had a remarkable impact on materials science due to their desirable electronic and self-assembly properties.^1^ A privileged subset of PAHs, heterocyclic PAHs, are highly valued in solar cells,^2^ electroluminescent materials^3^ and organic light emitting diodes (OLEDs)^4^ (*e.g.*, 1–3, Fig. 1). Indeed, heteroatom incorporation in these systems provides several functional and electronic advantages. This includes the introduction of nitrogen functional handles for synthetic manipulations, capacity for N-coordination to metal centers, the potential for donor–acceptor systems and usage as stimuli-responsive materials.^5^ Accordingly, concise and diversifiable synthetic methods for accessing heterocyclic PAHs are highly desirable.^6^

**Fig. 2:**
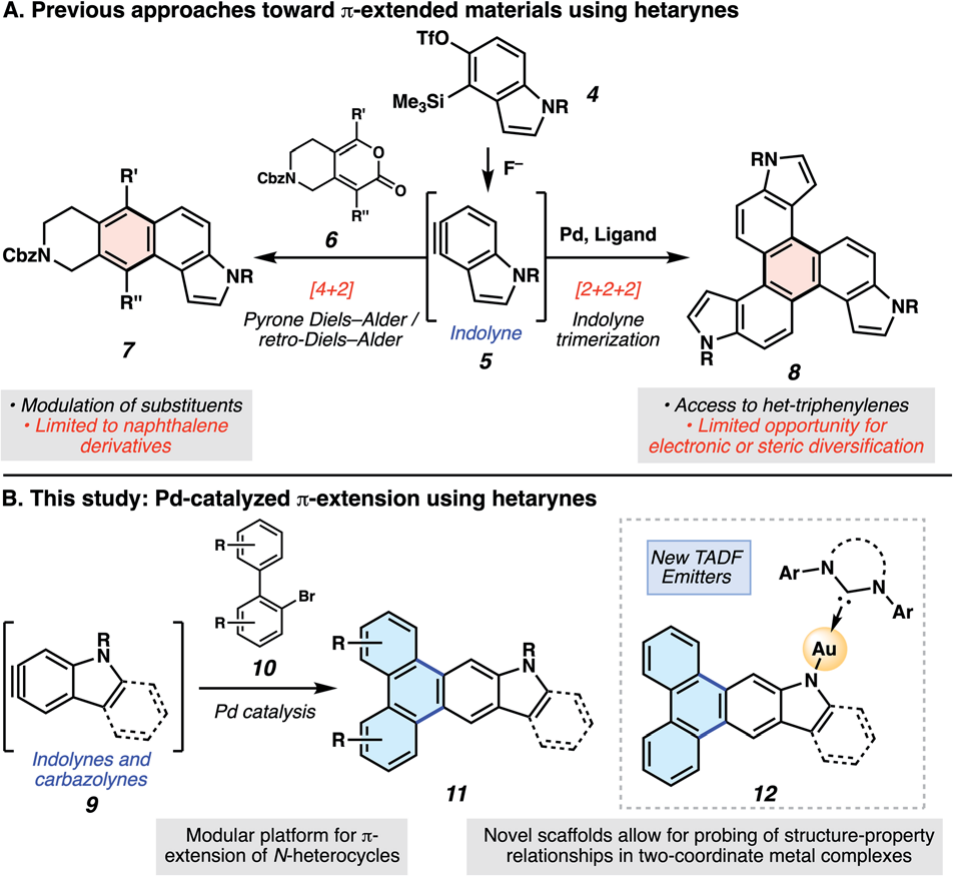
Approaches for the π-extension of hetarynes. (A) Prior studies involving the synthesis of π-extended materials using hetarynes. (B) Our studies, which leverage hetarynes in Pd-catalyzed annulations to access π-extended N-heterocycles and new TADF emitters.

An attractive approach toward heterocyclic PAHs involves assembling fused rings about a central heterocyclic building block through annulative π-extension (APEX).^6^ A compelling means to achieve this objective involves the use of *in situ* generated arynes, although such intermediates were historically avoided because of their high reactivity. However, the strain driven reactivity of arynes, along with the ability to form multiple bonds in a single step under mild conditions, has prompted the recent usage of arynes as modular building blocks in a wide array of applications, including in the synthesis of PAHs.^7–11^ In contrast, heterocyclic arynes (hetarynes) have only been used sparingly in PAH synthesis. This is in part due to the mild, fluoride-mediated generation of heterocyclic arynes (hetarynes) only becoming widespread in the past decade.^12^ Moreover, the ability to access and manipulate indole-derived arynes (indolynes) and their derivatives differs considerably from that of benzyne chemistry. The pyrrole ring can influence aryne structure and reactivity,^13^ and its nucleophilicity can often result in side reactions,^14^ posing numerous challenges for methodology development. Specifically, the reaction rate of pyrrole in electrophilic aromatic substitution reactions has been approximated to be roughly 3 × 10^18^ times faster than that of benzene.^15^Fig. 2A highlights two recent examples of the generation and capture of indolyne 5, which arises from indolyne silyl triflate precursor 4.^16–18^

In the present study, we aimed to develop a rapid, convergent approach to access heteroatom-containing PAH scaffolds. In particular, a method was devised that allows for the appendage of multiple aromatic rings to important heterocyclic building blocks in a controlled bimolecular reaction. This reaction provides access to a diverse set of aromatic products with electronic and steric modifications. The sequence we devised, inspired by pioneering studies by Larock,^19^ is shown in Fig. 2B. *In situ* generated hetarynes 9 would be united with bromobiaryl partners 10 using palladium catalysis to furnish heterocyclic PAHs 11.^20^ This annulative π-extension of hetarynes would expand on existing applications of hetaryne chemistry, create two new carbon–carbon (C–C) bonds, allow for the addition of three aromatic rings (shown in blue) to important heterocycles, and permit rapid access to heterocyclic triphenylene derivatives. Of note, this type of Larock annulation^19^ had not previously been achieved using nitrogen-containing, hetaryne intermediates. Additionally, metal-catalyzed transformations that utilize hetarynes are rare. Iwayama and Sato have reported [2+2+2] reactions of pyridynes.^21^ Only one study involving metal-catalyzed reactions of electron-rich N-containing hetarynes is available in the literature, as developed by our laboratory.^16^

**Scheme 1:**
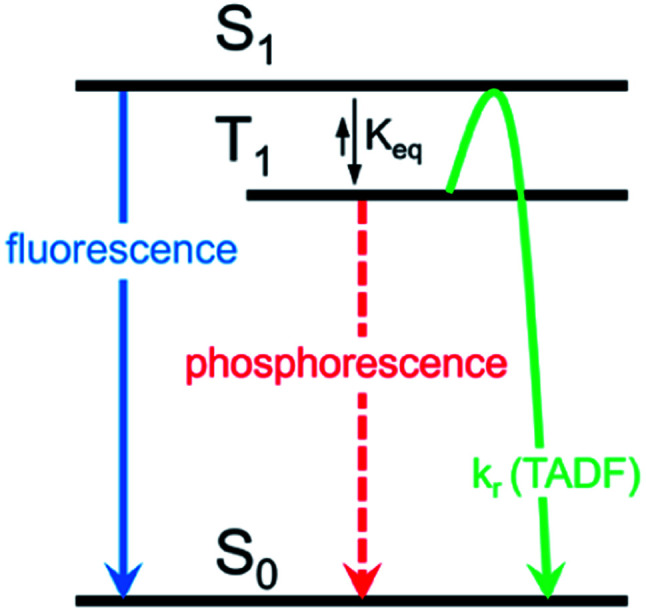
Mechanism for TADF emission compared to fluorescence and phosphorescence.

Herein, we describe the development of this methodology to access π-extended heterocyclic adducts, in addition to a concise synthetic route to a new carbazolyne precursor. We also show the utility of our methodology in the synthesis of π-extended ligands, which were utilized in novel two-coordinate metal complexes 12. Rapid access to complexes 12 allowed us to study the influence of extended conjugation on the efficiency of thermally activated delayed fluorescence (TADF) processes, which have received notable interest in recent years in the context of OLEDs.^22^ As described in Scheme 1, in OLEDs, singlets (S_1_) and triplets (T_1_) are generated upon hole and electron recombination. Fluorescence describes prompt decay from the S_1_ state, whereas phosphorescence describes delayed decay from the T_1_ state. In TADF emitters, T_1_ is thermally promoted to the S_1_ state, followed by radiative decay from S_1_. We show that extended π-conjugation can influence the performance of the TADF complex by modulating either the steric or electronic features of the ligand. Moreover, our studies permit access to a new TADF emitter that displays up to 81% photoluminescence efficiency.

## Results and discussion

**Fig. 3:**
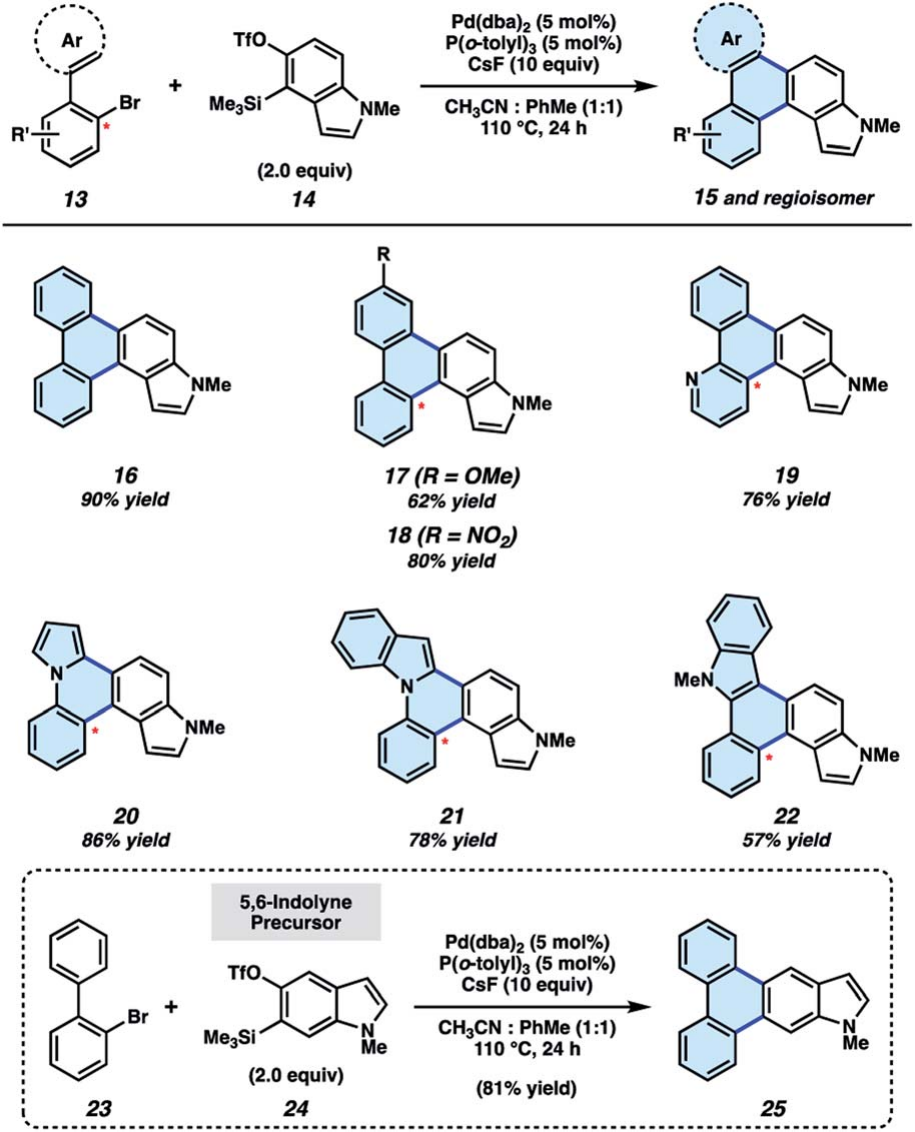
π-Extension of indolynes. Yields for 17–22 reflect isolated yields of a mixture of regioisomers (1–1.5 : 1; see ESI† for details).

We initiated our synthetic studies by pursuing a Pd-catalyzed annulation reaction of bromobiaryls 13 ^23^ with *N*-Me-4,5-indolyne precursor 14 (Fig. 3), the latter of which is accessible in a single step from its commercially available N–H derivative. In our initial studies, we used 2-bromobiphenyl as the aryl halide coupling partner and examined conditions reported by Larock for the annulation of carbocyclic arynes.^19^ This led to the formation of 16 in only 43% yield, highlighting the aforementioned challenges associated with using N-containing hetarynes in metal catalyzed reactions, as compared to simpler arynes. In prior studies,^24–26^ we found that metal-catalyzed trappings of heterocyclic strained intermediates necessitate careful optimization, as such reactions require that a transient strained intermediate be generated at a rate that allows for efficient reaction with an *in situ* generated organometallic species (*i.e.*, after 13 undergoes oxidative addition with Pd), while minimizing decomposition pathways commonly seen in strained intermediate chemistry. We ultimately found the desired reaction took place more efficiently by employing 5 mol% Pd(dba)_2_, 5 mol% P(*o*-tolyl)_3_, a 1 : 1 ratio of co-solvents acetonitrile and toluene, and 10 equivalents of cesium fluoride (CsF). The mixture of co-solvents, in particular, is thought to be important for modulating the rate of hetaryne formation.^27^ With optimal conditions, 16 could be accessed in 90% yield. Substituted biaryls could also be employed in the methodology. For example, methoxy- and nitro-substituted biaryls underwent the annulation smoothly to deliver adducts 17 and 18 in 62% and 80% yields, respectively. In these cases, mixtures of regioisomers are formed in roughly equal quantities.^28^ We also sought to incorporate additional heteroatoms into the products by employing heterocyclic derivatives of 2-bromobiphenyls. Use of a pyridyl substrate furnished 19 in 76% yield, which is an interesting aza-analog of parent compound 16. We were also able to replace one of the phenyl rings with pyrrole or indole units, as exemplified by the formation of 20–22. Lastly, we performed the annulation of 2-bromobiphenyl (23) with 5,6-indolyne precursor 24, which delivered adduct 25 in 81% yield. The results shown in Fig. 3 not only provide access to electronically and structurally diverse heteroatom-containing PAHs, but also validate our strategy to achieve the π-extension of heterocyclic building blocks.

Having validated the π-extension of indoles *via* the use of indolynes, we sought to develop analogous chemistry using the carbazole heterocycle. Carbazoles bear an additional aromatic ring in comparison to their indole counterparts and are valuable in materials-based applications,^29^ medicinal chemistry,^30^ and natural product total synthesis.^31^ However, aryne-derived carbazoles (carbazolynes) have seen sparce use in chemical synthesis. Recent examples involve carbazolyne generation from the hexadehydro-Diels–Alder reaction,^32^ the use of a silyl nonaflate precursor,^33^ and *via* classic dehydrohalogenation chemistry.^30,34^

**Fig. 4:**
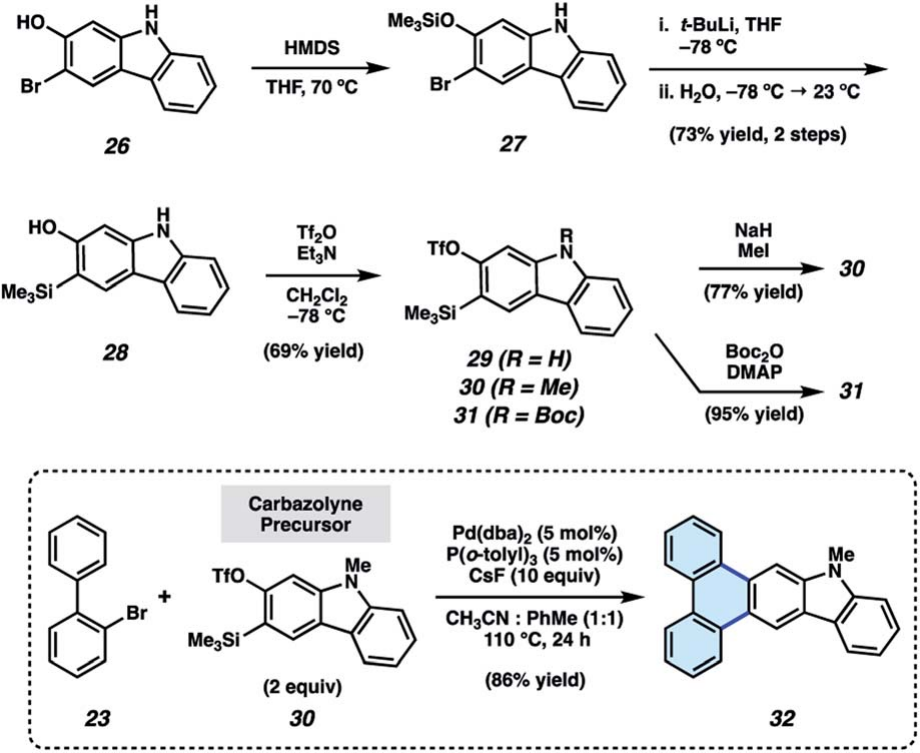
Synthesis of carbazolyne precursors.

As silyl triflate precursors to carbazolynes were not known in the literature, we developed the concise approach to carbazolyne precursors 29–31 shown in Fig. 4. 3-Bromo-2-hydroxycarbazole (26)^30,33^ was treated with HMDS to afford silyl ether 27, which, in turn, was carried forward in a retro-Brook rearrangement sequence to afford silyl alcohol 28. Triflation proceeded smoothly to deliver silyl triflate 29 in 69% yield. N–H compound 29 was elaborated to protected derivatives 30 and 31*via* methylation and Boc-protection, respectively. *N*-Me-carbazolyne precursor 30 was employed in our π-extension methodology using our previously optimized conditions. We were delighted to find that reaction of 2-bromobiphenyl (23) and *N*-Me-carbazolyne precursor 30 using Pd-catalysis furnished π-extended carbazole 32 in 86% yield. This is the first example of a transition metal-catalyzed trapping of a carbazolyne intermediate. Notably, this permits the one-step installment of a carbazole moiety into a π-extended system.

**Fig. 5:**
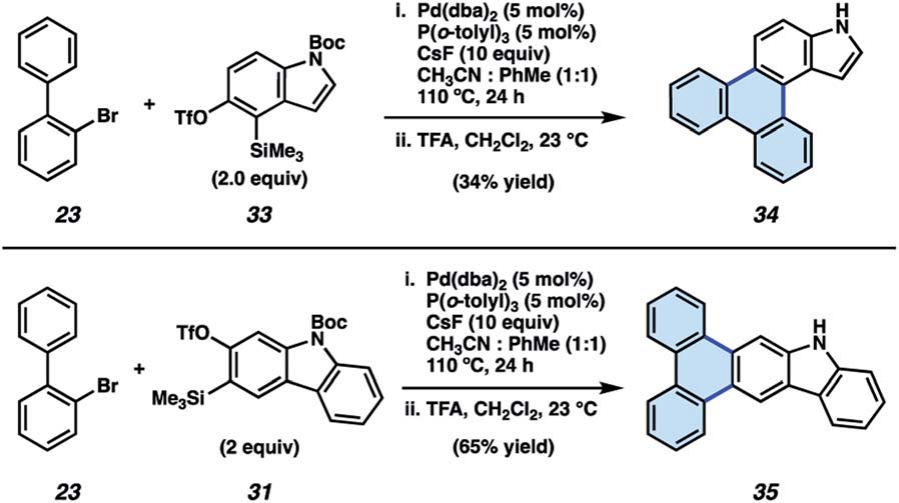
Synthesis of N–H products 34 and 35.

We also sought to determine if N–H derivatives of our indole and carbazole annulation products could be accessed using our methodology. It was ultimately found that N–H products were accessible by employing *N*-Boc protected hetaryne precursors in our methodology (Fig. 5).^35^ Subjection of 23 and indolyne precursor 33 to our standard reactions conditions, followed by treatment with trifluoroacetic acid (TFA) to remove the Boc protecting group, gave deprotected indole scaffold 34 in 34% yield. Similarly, N–H carbazole adduct 35 was accessed in 65% yield *via* the corresponding reaction of 23 and *N*-Boc-carbazolyne precursor 31. The ability to access π-extended N–H products (*e.g.*, 34 and 35) is expected to prove generally useful, as the N-position can be easily substituted. PAHs 34 and 35 also proved useful in our subsequent studies (*vide infra*) pertaining to the synthesis and evaluation of thermally activated delayed fluorescence (TADF) complexes.

**Fig. 6:**
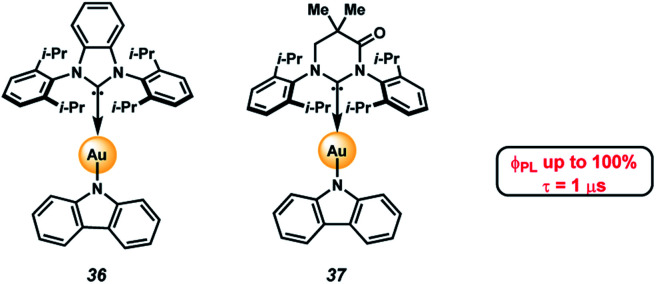
Heterocyclic PAHs as donor ligands in TADF complexes.^48,49^

A potential application of indole- and carbazole-based PAHs is in organic light emitting diodes (OLEDs), which are crucial for high-performance display technologies and solid-state lighting applications. Specifically, the recent development of TADF complexes of the general structure donor–M–acceptor, where M is Cu(i), Ag(i), or Au(i), and where the donor and acceptor are amide and carbene ligands, respectively, (*e.g.*, 36 and 37, Fig. 6),^36–40^ has allowed access to emissive dopants with high photoluminescence efficiencies (*Φ*_PLQY_ up to 100% in both solution and in the solid state) and short-lived excited states (*τ* < 1 μs). These compounds also offer potential economic advantages over Ir- or Pt-centered phosphorescent dopants.^41,42^

The photophysical properties of these two-coordinate complexes can be manipulated by altering either the donor or acceptor ligands^43^. For example, compounds 36 and 37 have the same amide donor ligand (carbazolyl) but different acceptor carbene ligands. Consequently, the energy for the interligand charge transfer (ICT) transition is different in each complex. Compound 36, with the carbene BZI (1,3-bis(2,6-diisopropylphenyl)-1-*H*-benzo[*d*]imidazole-2-ylidene) as an acceptor ligand emits at 432 nm when doped in polystyrene (PS) films whereas 37, which has MAC (1,3-bis(2,6-diisopropylphenyl)-5,5-dimethyl-4-keto-tetrahydropyridylidene) as an acceptor, emits at 506 nm in the same media. The difference in energy for the ICT state is due to the poorer electrophilicity of BZI (36, *E*_red_ = −2.82 V *vs.* Fc^+^/Fc) relative to MAC (37, *E*_red_ = −2.50 V). The low electron affinity of BZI raises the energy of the ^3^ICT state such that it is near resonant with that of the locally excited triplet state (^3^LE) of carbazolyl (^3^LE = 415 nm). It follows that altering the nature of amide donor will also modify the energy of the ICT state, and consequently the photophysical properties of the complex.

Previous studies on carbazolyl donor ligands have focused on electronic modifications of carbazole using electron-donating or withdrawing groups, steric hindrance, and structural flexibility, leading to important structure–property relationships.^36*c*,40,43–45^ Moreover, extending the conjugation in aromatic π-systems by benzannulation has been shown to impact relative HOMO and LUMO energy levels in ways that sometimes lead to counterintuitive changes in emission color.^42,46,47^ Thus, the effect of similar π-extension on the photophysical properties of donor–M–acceptor complexes may not be obvious. Heterocyclic PAHs accessible by our π-extension methodology provide an opportunity to study and compare new π-extended TADF complexes.

**Fig. 7:**
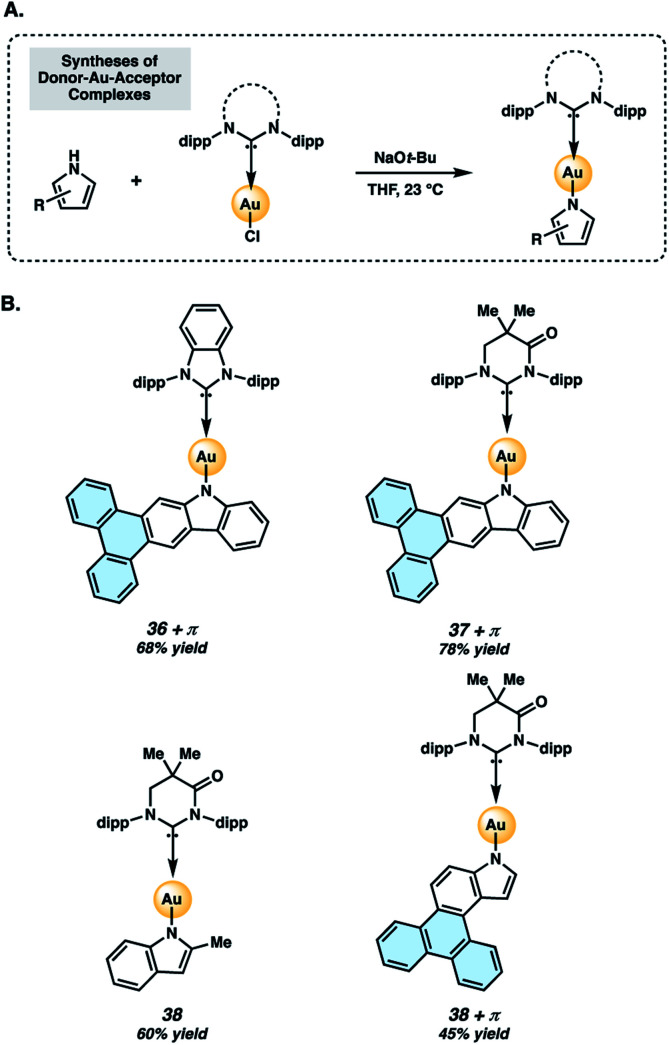
Preparation (A) and structures (B) of two-coordinate gold complexes. Dipp = 2,6-diisopropylphenyl, as shown in Fig. 6.

Two-coordinate donor–Au–acceptor complexes were prepared by treatment of 34 or 35 with sodium *tert*-butoxide in the presence of the NHC–Au–Cl complex (Fig. 7A). The metal complexes evaluated in the present study (Fig. 7B) are Au complexes due to their superior stability compared to Ag or Cu analogs.^40^ Complexes 36+π and 37+π were obtained in 68% and 78% yields, respectively, whereas the respective indolyl complexes 38 and 38+π were prepared in 60% and 45% yields using this protocol. Complexes 36+π and 37+π enable comparison of the new π-extended phenanthrocarbazolyl donors to their respective carbazolyl counterparts, 36 ^48^ and 37.^43,49^ The methylindolyl (38) and phenanthroindolyl (38+π) derivatives allow us to assess the impact of π-extension within a new indole series of complexes, as well as compare the carbazolyl and indolyl complexes.

**Fig. 8:**
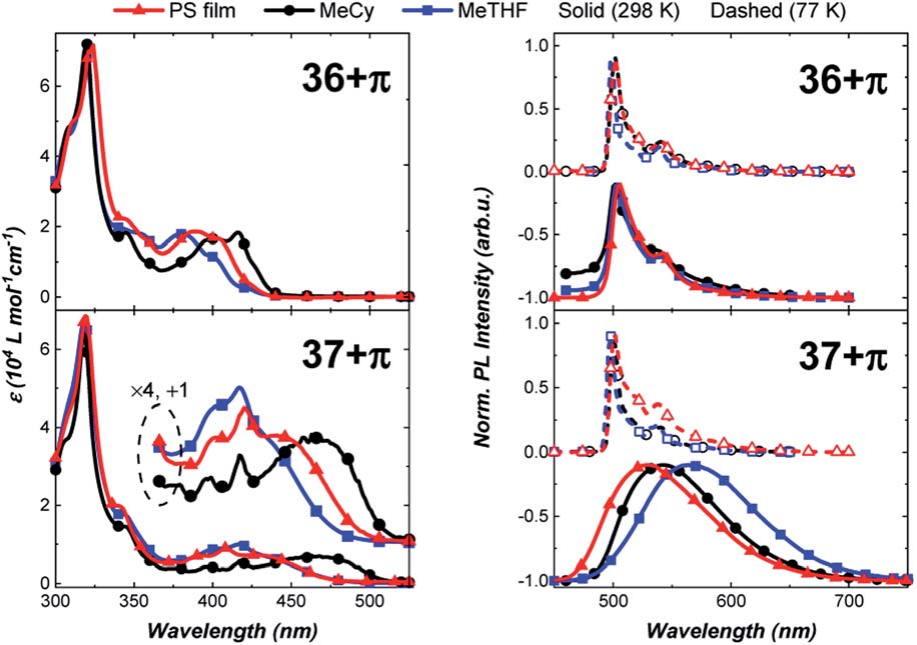
Absorption (left) and emission spectra (right) for the carbene–Au–carbazolyl complexes. Note that the absorption spectra of the PS films were scaled to match the intensity of the MeCy solution at *λ*_max_ = 320 nm. This feature is assigned to a solvent insensitive transition localized on the carbazolyl ligand.

The photophysical properties of the π-extended carbazolyl-based complexes, 36+π and 37+π, were examined in different media (Fig. 8) and compared to data of the parent carbazole-containing complexes 36 and 37 (Table 1).^43,48,49^ The UV-visible absorption spectra of 36+π and 37+π display a strong solvent-independent band at 320 nm attributed to a π–π* transition localized on the phenanthrocarbazolyl ligand. Weaker bands at lower energy are more structured and display a pronounced negative solvatochromism (*e.g.*, 36+π at 416 nm in MeCy and 382 nm in MeTHF; 37+π at 460 nm in MeCy and 417 nm in MeTHF). This band is assigned to the ICT transition between the π-extended carbazole donor and carbene acceptor ligand that is overlapped with π–π* transitions on the phenanthrocarbazolyl ligand. The solvatochromic behavior of the ICT band is ascribed to the dipole moment interactions between the solvent and complex molecules, in which the dipole of the excited state is larger and is oriented in the opposite direction as that of the ground state. The energy of the ICT transition in 36+π and 37+π is comparable to values for 36 and 37 (see Table 1) indicating that the donor strength of the phenanthrocarbazolyl and carbazolyl ligands are nearly equivalent.

**Table 1:** Photophysical data for carbene–Au–carbazolyl complexes

Complex	Solvent	*λ*_abs_ a	*λ*_em,298 K_ (*λ*_77 K_)	*Φ*_PLQY_	*τ*_298 K_ (10^−6^ s)	*k*_r_ (10^5^ s^−1^)	*k*_nr_ (10^5^ s^−1^)	*τ*_77 K_ (10^−3^ s)
36 ^48^	1% PS film	385	452 (426)	1.00	3.6 (54%)	4.4^b^	<0.04^b^	0.19
					0.74 (46%)			
	MeCy	405	424 (424)	0.89	1.2	7.8	0.9	0.34
	MeTHF	365	452 (426)	0.79	2.6	3.0	0.8	0.64
36+π	1% PS film	388	506 (502)	0.52	5.1 (70%)	3.1^b^	11^b^	1.8
					6.7 (30%)			
	MeCy	416	502 (504)	0.03	30.0	0.01	0.3	2.9 (90%)
								14 (8%)
								100 (2%)
	MeTHF	382	504 (498)	0.02	57	0.004	0.2	3.2 (94%)
								33 (6%)
37 ^49^	1% PS film	425^c^	512 (506)	0.85	0.83	10.0	1.8	0.043
	MeCy	450	522 (456)	0.88	1.1	8.0	1.1	0.068
	MeTHF	412	544 (428)	0.50	0.79	6.3	6.3	0.26
37+π	1% PS film	420	529 (500)	0.74	3.3 (47%)	3.4^b^	1.2^b^	4.4 (43%)
					1.0 (45%)^d^			2.2 (33%)
								0.7 (24%)
	MeCy	460	544 (500)	0.81	0.9 (91%)^d^	9.1	2.1	1.8 (59%)
								3.1 (41%)
	MeTHF	417	566 (498)	0.39	0.6 (96%)^d^	6.9	11	2.4 (72%)
								5.3 (28%)

a ICT band.

b Calculated from the weighted averages of both contributions.

c Obtained from excitation spectrum.

d An additional minor contribution from a longer lifetime component is needed to fit the observed data and assigned to p-type delayed fluorescence. See ESI.

**Table 2:** Photophysical data for carbene–Au–indolyl complexes

Complex	Solvent	*λ*_abs_ a	*λ*_em,298 K_ (*λ*_77 K_)	*Φ*_PLQY_	*τ*_298 K_ (10^−6^ s)	*k*_r_ (10^5^ s^−1^)	*k*_nr_ (10^5^ s^−1^)	*τ*_77 K_ (10^−3^ s)
38	1% PS film	414	534 (525)	0.12	0.71 (80%)	2.5^b^	14^b^	0.067 (78%)
					0.25 (20%)			0.025 (22%)
	MeCy	435	565 (484)	0.03	0.13	2.5	74	0.10
	MeTHF	399	600 (476)	0.004	0.011	3.6	910	0.11
38+π	1% PS film	394	519 (495)	0.49	1.7 (65%)	3.9^b^	2.1^b^	7.0 (74%)
					0.41 (35%)			3.0 (26%)
	MeCy	420	530 (486)	0.24	0.54	4.1	14	9.3 (62%)
								13 (38%)
	MeTHF	380^a^	566 (482)	0.01	0.024	5.8	4100	14 (73%)
								19 (27%)

a ICT band.

b Calculated from the weighted averages of both contributions.

The effect of π-extension on the amide ligand is manifested more evidently in the luminescence properties of the complexes. In particular, whereas luminescence from 36 is broad and solvatochromic, emission spectra of 36+π are narrow, red-shifted and independent of solvent polarity. Radiative (*k*_r_) and non-radiative (*k*_nr_) rate constants are calculated using the relationship *k*_r_ = *Φ*_PL_/*τ*, where *Φ*_PL_ = *k*_r_/(*k*_r_ + *k*_nr_). The radiative rate for emission from 36 is rapid (*k*_r_ = 3.0 × 10^5^ s^−1^ in MeTHF) as opposed to being markedly slow in 36+π (*k*_r_ = 4 × 10^2^ s^−1^ in MeTHF). Unlike the photophysical properties of 36, which are characteristic of emission from an ICT state, luminescence from 36+π indicates that the excited state transitions are localized on the donor ligand, and hence undergo conventional phosphorescence rather than TADF. This assignment for the luminescence is confirmed by the minimal shift in energy and millisecond emission lifetime found upon cooling to 77 K. The difference in properties for 36+π*versus*36 is caused by the lower energy for ^3^LE state of the phenanthrocarbazolyl moiety (see ESI†) compared to that for the carbazolyl ligand. In contrast, complex 37+π is capable of efficient TADF from the ICT state as borne out by emission spectra (Fig. 8) that are similar, albeit redshifted, to spectra reported for 37. The fast radiative rate (*k*_r_ = 9.1 × 10^5^ s^−1^) and high photoluminescence efficiency (*Φ*_PLQY_ = 0.81) at room temperature in MeCy, along with luminescence that is redshifted from polar solvent (MeTHF) to non-polar solvent (MeCy and polystyrene film), is also consistent with emission from an ICT state.^50^ Emission at 77 K (500 nm) is structured and polarity-independent. In this case, solvent molecules are frozen as a glass around the complex molecules, thus restricting stabilization of the excited ICT triplet.^43^ Therefore, the triplet state localized on the donor becomes the lowest-lying emissive state and precludes ICT events. Notably, 37+π achieved 81% photoluminescence efficiency in MeCy (Fig. 8).

**Fig. 9:**
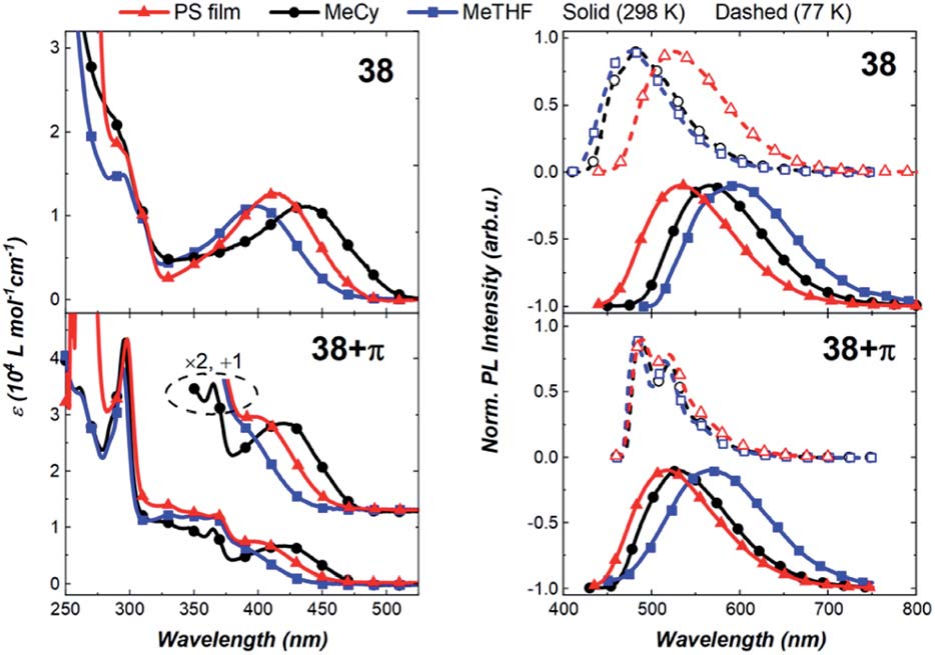
Absorption (left) and emission spectra (right) for carbene–Au–indolyl complexes. Note that the absorption spectra of the PS films were scaled to match the peak at *λ*_max_ = 295 nm in MeCy solution, which is assigned to a solvent insensitive transition localized on the indolyl ligand.

Next, we compared the optical properties of 38 and 38+π (Fig. 9 and Table 2). The ICT and π–π* absorption bands are more resolved in complex 38 than in 38+π. The high extinction coefficients for the ICT band of 38 suggest strong electronic coupling between the acceptor carbene and the 2-methylindolyl donor ligands. Emission spectra for both complexes at 298 K show broad featureless bands and radiative rates are relatively fast (*k*_r_ > 10^5^ s^−1^), both characteristics consistent with decay from an ICT excited state. The luminescence from 38 is slightly red-shifted (*e.g.*, *λ*_MeTHF_ = 600 nm) relative to spectra from 38+π (*e.g.*, *λ*_MeTHF_ = 566 nm), indicating that 2-methylindole is a stronger donor than the phenanthroindolyl ligand, which can be attributed to the different position of the π-extension in 38+π compared to 36+π and 37+π. Luminescence from the methylindolyl-based complex remains broad and featureless upon cooling from 298 K to 77 K, indicating that the transition retains ICT character even in frozen matrix. For 38, note that the destabilization of the ICT state upon going from 298 K to 77 K is greater in MeCy and MeTHF than in the PS film. This is likely due to enhanced solute–solute interactions on cooling the fluid solutions to 77 K, whereas the relative orientations of the solutes are fixed in a more random fashion at room temperature in the PS film. In contrast, the emission spectrum of 38+π is structured at 77 K and assigned to a low-lying ^3^LE transition on the phenanthroindolyl ligand. The short lifetime measured for 38 at 77 K in MeTHF (*τ* = 1.1 × 10^−4^ s) compared to that for 38+π (*τ* = 1.5 × 10^−2^ s) is consistent with an ^3^ICT transition for the former complex and ^3^LE phosphorescence for the latter derivative.

Complex 38+π exhibits higher photoluminescence quantum yields in all media compared to 38. The yields increase from MeTHF (*Φ*_PLQY_ = 1.4%) to MeCy (*Φ*_PLQY_ = 24.2%) to the PS film (*Φ*_PLQY_ = 49%). The lower emission efficiency in polar solvents is attributed to the greater reorganization of the excited state structure in polar solvents. Generally, complex 38 displays comparable, although slower radiative rates (*k*_r_) and faster non-radiative rates (*k*_nr_) in all solvents relative to 38+π. The slower *k*_nr_ of 38+π is likely due to a slower rate of rotation or exchange caused by the larger free volume of the 38+π ligand.

Comparing the carbazolyl and indolyl MAC complexes, it is noted that both appear to undergo TADF emission; however, the former (carbazolyl) family exhibits higher photoluminescence efficiencies due to lower rates of non-radiative decay. In comparing 38 to 38+π, the low steric profile about the indolyl ligand appears to have a greater influence on the photophysical performance than any energetic differences. In contrast, the discrepancy in performance between 36 and 36+π is attributed primarily to the energetics induced by π-conjugation. In other words, the photophysical properties of indolyl-based complexes are primarily influenced by decreased steric hindrance of the indolyl ligand, whereas the performance of the carbazolyl-based complexes is primarily dictated by the π-conjugation thermodynamics.

## Conclusions

In summary, we have developed a modular platform to access N-heterocycles with extended π-conjugation by leveraging hetarynes and Pd-catalysis. Of note, these represent rare examples of metal-mediated transformations of N-heterocyclic arynes. Through the construction of two C–C bonds in a single operation, this methodology allows for the direct π-extension of heterocyclic scaffolds through the appendage of three or more aromatic rings. The methodology offers a convergent platform for accessing important heterocycles with structural and electronic diversity. Notably, a new carbazolyne precursor, whose synthesis relies on a key retro-Brook rearrangement, can also be leveraged in this reaction to access carbazole derivatives. Heterocycles accessed in our methodology were ligated to Au–NHC complexes to give new two-coordinate metal complexes.

We find that extending the π-conjugation of the donor ligand influences the photophysical properties of the two-coordinate Au(i)–NHC complexes. The principal effect of π-extension in these compounds is stabilization of the triplet energy as opposed to only a minor perturbation of the donor strength. Therefore, depending on the nature of the carbene paired with the donor ligand, luminescence can be tuned to achieve emission from either the ^3^LE or ICT state. Thus, a relatively weak electron accepting carbene such as BZI in 36 and 36+π gives only inefficient ^3^LE emission, whereas the stronger electron accepting MAC carbene in 37 and 37+π leads to efficient emission from the ICT state. The carbazolyl (37 and 37+π) and indolyl (38 and 38+π) complexes allow for the direct comparison of two systems that undergo TADF emission from the ICT state. The π-extension of the carbazolyl ligand in 37+π leads to a red-shift in emission. However, π-extending the indolyl-based ligand in 38+π results in an unexpected blue-shift in emission energy that may owe to the different position of substitution.^42^

These studies should prompt further structure–photophysical property studies of donor ligands in these metal complexes to enhance OLED stability and efficiency. Furthermore, these studies demonstrate that hetarynes can be strategically leveraged as central building blocks for accessing π-extended scaffolds with notable properties.

## Data availability

Full details on the synthesis and characterization of compounds, photophysical data, and computational methods are accessible in the ESI.†

## Author contributions

K. A. S., J. V. C., and R. B. S. designed and performed experiments and analyzed experimental data pertaining to the heterocyclic PAHs. M. D. and N. U. synthesized, characterized, and studied the photophysical properties of the metal complexes. P. I. D., M. E. T., and N. K. G. directed the investigations and prepared the manuscript with contributions from all authors; all authors contributed to discussions.

## Conflicts of interest

One of the authors (Mark E. Thompson) has a financial interest in the Universal Display Corporation.

## Supplementary Material

SC-013-D2SC01788A-s001
