# Supplementary material for: π-Extension of heterocycles via a Pd-catalyzed heterocyclic aryne annulation: π-extended donors for TADF emitters
Source: Chem Sci. 2022 May 4;13(20):5884–92. doi: 10.1039/d2sc01788a (PMC9132060; doi:10.1039/d2sc01788a)
Supplement: SC-013-D2SC01788A-s001 [file SC-013-D2SC01788A-s001.pdf]

Electronic Supplementary Material (ESI) for Chemical Science.
This journal is © The Royal Society of Chemistry 2022

π-Extension of Heterocycles via a Pd-Catalyzed Heterocyclic Aryne Annulation: π-Extended Donors for TADF Emitters

Katie A. Spence,[§,†] Jason V. Chari,[§,†] Mattia Di Niro,[⊥] Robert B. Susick,[†] Narcisse Ukwitegetse,[⊥] Peter I. Djurovich,[⊥] Mark E. Thompson,*[,⊥] and Neil K. Garg*[,†]

[†]*Department of Chemistry and Biochemistry, University of California at Los Angeles, Los Angeles, California 90095, United States*

[⊥] *Department of Chemistry, University of Southern California, Los Angeles, California 90089, United States*

Electronic Supplementary Information – Table of Contents

Materials and Methods S2

Experimental Procedures S4
- **A. Scope of Pd-Catalyzed Annulation with *N*-Me-4,5-Indolyne S4**
- **B. Synthesis of Silyl Triflate Precursor to 2,3-Carbazolyne S10**
- **C. Annulation of *N*-Me-Carbazolyne S13**
- **D. Synthesis of N–H Annulation Products for Metal Coordination S14**
- **E. Synthesis of Two-Coordinate Metal Complexes S16**
- **F. General Procedure for Photophysical Property Analyses S20**
- **G. Absorption Spectra of Donor Ligands S20**
- **H. Emission Spectra of Deprotonated Donor Ligands S20**

Computational Methods S22
- **A. Complete Citation of Q-Chem 5.1 .... S22**

Photoluminescence (PL) Decay Lifetime Data S23
- **A. Lifetime Plots S23**

^{1}H NMR Spectra S29
^{13}C NMR Spectra S50
NOESY Spectra S68

References.. S71

Materials and Methods

Unless stated otherwise, reactions were conducted in flame-dried glassware under an atmosphere of nitrogen or argon and commercially obtained reagents were used as received. Anhydrous solvents were either freshly distilled or passed through activated alumina columns, unless otherwise stated. Reaction temperatures were controlled using an IKAmag temperature modulator and, unless stated otherwise, reactions were performed at room temperature (approximately 23 °C). Cesium Fluoride (CsF) and Bis(dibenzylidenacetone)palladium(0) ($Pd(dba)_2$) were obtained from Strem Chemicals and stored in a desiccator. Tri(*o*-tolyl)phosphine ($P(o\text{-tolyl})_3$), 1,1,1,3,3,3-hexamethyldisilazane (HMDS), *tert*-butyllithium (*t*-BuLi), sodium hydride (NaH), and sodium *ter*-butoxide (NaO*t*-Bu) were obtained from Sigma Aldrich. Methyl iodide (MeI) was acquired from Spectrum Chemical. Triflic anhydride was purchased from Oakwood Chemical and distilled over phosphorous pentoxide prior to use. Di-tert-butyl decarbonate and 4-dimethylaminopyridine (DMAP) were obtained from Oakwood Chemical. Triethylamine was purchased from Fischer Scientific and passed through an activated alumina column prior to use. 2-Bromobiphenyl (**23**) was obtained from Combi-Blocks and purified by flash chromatography (100% Hexanes) prior to use. 3-Bromo-2-phenylpyridine (**43**) was obtained from Combi-Blocks. Bromobiaryls **39**,[1] **41**,[2] **45**,[3] **47**,[4] and **49**,[5] were prepared according to literature procedures. The silyl triflates **14**,[6] **33**,[7] and **24**[7] were prepared following literature procedures. 3-Bromo-2-hydroxycarbazole (**26**) was prepared in one step from 2-hydroxycarbazole following a literature protocol.[8] NHC–Au–Cl complexes **51**[9] and **52**[10] were prepared following literature procedures. Regioisomeric ratios for indolyne annulation products were determined by analysis of the ^{1}H NMR spectra of the crude reaction mixtures. Thin-layer chromatography (TLC) was conducted with EMD gel 60 F254 pre-coated plates (0.25 mm for analytical chromatography and 0.50 mm for preparative chromatography) and visualized using UV(254 nm). Celite® was purchased from Fischer Scientific and used as received. Silicycle Siliaflash P60 (particle size 0.040–0.063 mm) was used for flash column chromatography. ^{1}H NMR spectra were recorded on Bruker spectrometers (at 400, 500 and 600 MHz) and are reported relative to residual solvent signals. Data for ^{1}H NMR spectra are reported as follows: chemical shift (δ ppm), multiplicity, coupling constant (Hz), integration. Data for ^{13}C NMR are reported in terms of chemical shift (at 101 and 125 MHz). IR spectra were recorded on a Perkin-Elmer UATR Two FT-IR spectrometer and are reported in terms of frequency absorption (cm^{-1}). DART-MS spectra were collected on a Thermo Exactive Plus MSD (Thermo

Scientific) equipped with an ID-CUBE ion source and a Vapur Interface (IonSense Inc.). Both the source and MSD were controlled by Excalibur software v. 3.0. The analyte was spotted onto OpenSpot sampling cards (IonSense Inc.) using CH_2Cl_2 as the solvent. Ionization was accomplished using UHP He plasma with no additional ionization agents. The mass calibration was carried out using Pierce LTQ Velos ESI (+) and (–) Ion calibration solutions (Thermo Fisher Scientific). UV-visible absorption spectra were recorded by using Hewlett-Packard 4853 diode array spectrometer. Steady state emission spectra were recorded on Quanta-Master Photon Technology International phosphorescence/fluorescence spectrofluorometer. Emission quantum yields were measured using Hamamatsu C9920 system equipped with a xenon lamp, integrating sphere and model C10027 photonic multichannel analyzer (PMA). Emission lifetimes were acquired on IBH Fluorocube instrument by using time-correlated single photon counting (TCSPC) method.

Experimental Procedures

A. Scope of Pd-Catalyzed Annulation with *N*-Me-4,5-Indolyne

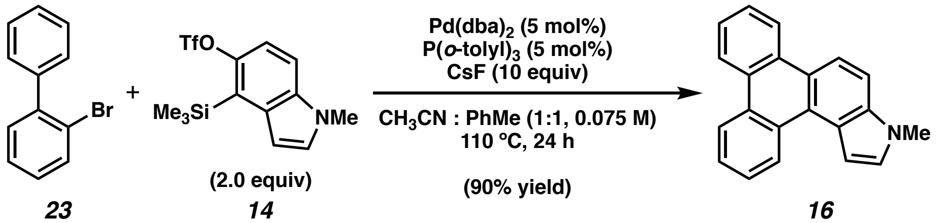

23 *14* *16*

Representative Procedure A for hetaryne annulations (Figure 3, annulation product 16 used as an example). A 1-dram vial was charged with Pd(dba)$_2$ (3.7 mg, 0.064 mmol, 5 mol%). Next, toluene (0.86 mL), P(*o*-tolyl)$_3$ (2.0 mg, 0.064 mmol, 5 mol%), 2-bromobiphenyl (**23**) (30.0 mg, 0.129 mmol, 1.0 equiv), silyl triflate **14** (90.5 mg, 0.257 mmol, 2.0 equiv), and acetonitrile (0.86 mL) were added, followed by an oven-dried magnetic stirbar and then CsF (195 mg, 1.29 mmol, 10 equiv). The vial was purged with nitrogen for 3 minutes, then sealed with a Teflon-lined screw cap and stirred at 110 °C for 24 h. Then, after cooling to 23 °C, the mixture was transferred with CH_2Cl_2 (10 mL) and H_2O (2 mL) to a 150 mL separatory funnel containing brine (15 mL). The layers were separated and the aqueous layer was extracted with CH_2Cl_2 (3 x 15 mL). The combined organic layers were dried over $MgSO_4$, filtered, and concentrated *in vacuo*. The resulting crude product was purified by flash chromatography (100% Hexanes → 200:1 Hexanes:EtOAc) to afford annulation product **16** (90% yield, average of two experiments) as an off-white solid. Indole **16**: mp: 139–144 °C; R_f 0.41 (4:1 Hexanes:EtOAc); ^{1}H NMR (500 MHz, $CDCl_3$): δ 9.24 (d, J = 8.0, 1H), 8.78 (d, J = 8.0, 1H), 8.72 (t, J = 7.0, 2H), 8.57 (d, J = 9.0, 1H), 7.74 (t, J = 7.4, 1H), 7.73–7.62 (m, 4H), 7.53 (d, J = 2.8, 1H), 7.28 (d, J = 3.0, 1H), 3.92 (s, 3H); ^{13}C NMR (125 MHz, $CDCl_3$): δ 136.4, 131.3, 131.2, 130.4, 128.9, 128.8, 127.17, 127.15, 126.7, 126.4, 125.9, 124.6, 124.1, 123.8, 123.6, 123.28, 123.25, 117.7, 110.5, 104.0, 33.3; IR (film): 3069, 2924, 2850, 1514, 1492, 1441, 1417, 1351, 1248, 754, 740, 718 cm^{-1}; HRMS-APCI (*m/z*) $[M + H]^+$ calcd for $C_{21}H_{16}N^+$, 282.12773; found 282.12717.

Any modifications of the conditions shown in the representative procedure above are specified in the following schemes, which depict all of the results shown in Figure 3.

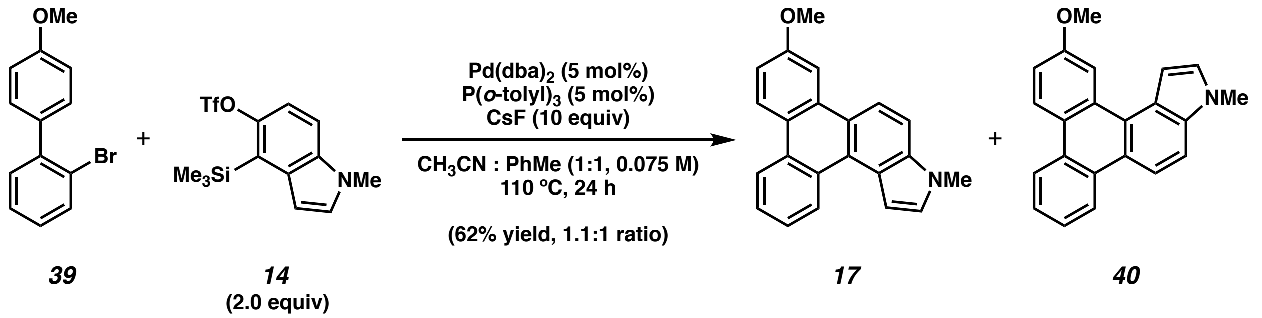

Indoles 17 and 40. Followed representative procedure A. Purification by flash chromatography (50:1 Hexanes:EtOAc) afforded an inseparable mixture of indoles **17** and **40** (62% yield, average of two experiments, 1.1:1 ratio, unassigned) as a white solid. Indoles **17** and **40**: R_f 0.27 (4:1 Hexanes:EtOAc); ^{1}H NMR (500 MHz, C_6D_6, combined): δ 9.42 (dd, J = 8.3, 1.3, 1H), 8.93 (d, J = 2.6, 1H), 8.69 (dd, J = 7.9, 1.3, 1H), 8.57–8.50 (m, 5H), 8.48 (d, J = 9.0, 1H), 8.22 (d, J = 2.5, 1H), 7.60–7.47 (m, 6H), 7.30–7.26 (m, 3H), 7.21 (dd, J = 9.2, 2.6, 1H), 6.72 (d, J = 6.7, 1H), 6.69 (d, J = 3.2, 1H), 3.61 (s, 3H), 3.55 (s, 3H), 3.001 (s, 3H), 2.995 (s, 3H); ^{13}C NMR (125 MHz, C_6D_6, combined): δ 159.6, 159.3, 136.8, 136.6, 133.32, 133.31, 131.2, 131.0, 130.8, 129.7, 128.6, 128.5, 127.6, 126.6, 126.4, 126.2, 126.0, 125.8, 125.4, 125.3, 125.2, 125.1, 125.0, 124.6, 124.5, 124.4, 124.0, 123.6, 123.27, 123.25, 118.1, 118.0, 115.5, 115.0, 110.8, 110.5, 110.1, 106.5, 104.5, 104.0, 55.0, 54.9, 32.31, 32.28; IR (film): 2934, 2834, 1614, 1510, 1414, 1246, 1227 cm^{-1}; HRMS-APCI (*m/z*) [M + H$^+$] calcd for $C_{22}H_{18}NO^+$, 312.13829; found 312.13897.

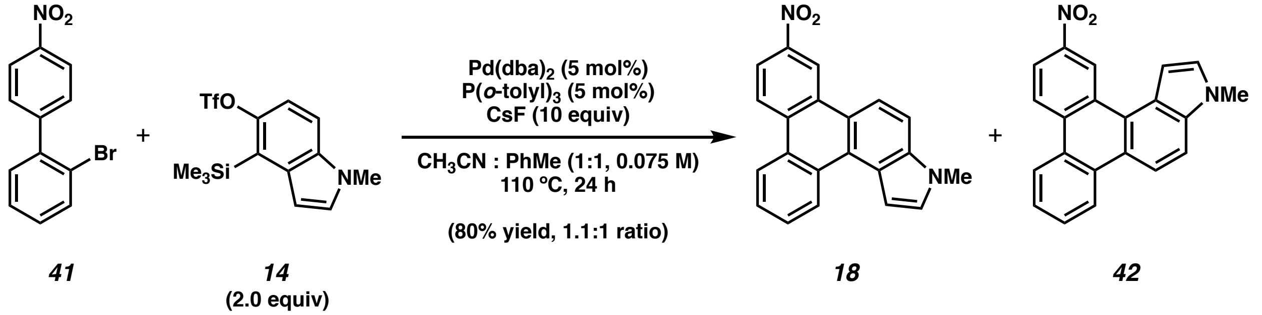

Indoles 18 and 42. Followed representative procedure A. Purification by flash chromatography (50:1 Hexanes:EtOAc → 20:1 Hexanes:EtOAc) afforded an inseparable mixture of indoles **18** and **42** (80% yield, average of two experiments, 1.4:1 ratio, unassigned) as a yellow solid. Indoles **18** and **42**: R_f 0.45 (4:1 Hexanes:EtOAc); ^{1}H NMR (600 MHz, $CDCl_3$, major): δ 10.10 (d, J = 2.4, 1H), 8.79 (d, J = 9.1, 1H), 8.71 (d, J = 8.3, 1H), 8.66 (d, J = 8.2, 1H), 8.55 (d, J = 9.2, 1H), 8.41 (dd, J = 9.0, 2.3, 1H), 7.78–7.72 (m, 2H), 7.67 (ddd, J = 8.1, 7.0, 1.2, 1H), 7.54 (d, J = 3.1, 1H),

7.39 (d, J = 3.2, 1H), 4.00 (s, 3H); ^{1}H NMR (600 MHz, $CDCl_3$, minor): δ 9.54 (d, J = 2.3, 1H), 9.21 (d, J = 8.3, 1H), 8.75 (d, J = 9.1, 1H), 8.70 (d, J = 8.3, 1H), 8.55 (d, J = 8.9, 1H), 8.35 (dd, J = 9.0, 2.3, 1H), 7.82 (ddd, J = 8.1, 7.0, 1.3, 1H), 7.76–7.71 (m, 2H), 7.51 (d, J = 3.1, 1H), 7.36 (d, J = 3.2, 1H), 4.00 (s, 3H); ^{13}C NMR (125 MHz, $CDCl_3$, combined): δ 146.4, 146.0, 136.8, 136.4, 134.8, 133.3, 132.51, 132.48, 131.3, 131.0, 129.9, 129.4, 129.0, 128.7, 128.6, 127.4, 127.3, 126.8, 126.4, 125.2, 124.6, 124.4, 124.21, 124.17, 124.1, 123.8, 123.60, 123.55, 123.5, 123.1, 122.8, 119.9, 119.53, 119.47, 117.5, 117.4, 111.7, 111.1, 104.0, 103.4, 33.4, 33.3; IR (film): 2919, 2852, 1597, 1515, 1346, 854, 747 cm^{-1}; HRMS-APCI (*m/z*) [M + H$^+$] calcd for $C_{21}H_{15}N_2O_2^+$, 327.11280; found 327.11387.

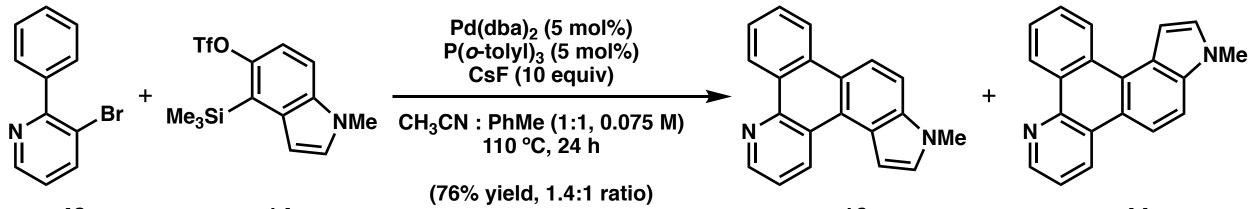

43 14 (2.0 equiv) 19 44

Indoles 19 and 44. Followed representative procedure A. Purification by flash chromatography (100% Hexanes → 50:1 Hexanes:EtOAc → 9:1 Hexanes:EtOAc) afforded an inseparable mixture of indoles **19** and **44** (76% yield, 1.4:1 ratio, average of two experiments) as a pale yellow solid. Indole **19**: R$_f$ 0.34 (4:1 Hexanes:EtOAc); ^{1}H NMR (500 MHz, $CDCl_3$): δ 9.40 (ddd, J = 8.2, 4.7, 1.4, 2H), 8.99 (dd, J = 4.3, 1.6, 1H), 8.66 (d, 8.2, 1H), 8.54 (d, 8.5, 1H), 7.76 (ddd, J = 8.1, 6.9, 1.6, 1H), 7.71 (ddd, J = 8.3, 6.8, 1.2, 1H), 7.65 (d, J = 9.0, 1H), 7.64–7.60 (m, 1H), 7.38 (d, J = 2.9, 1H), 7.27 (d, J = 3.2, 1H), 3.90 (s, 3H). Indole **44**: R$_f$ 0.34 (4:1 Hexanes:EtOAc); ^{1}H NMR (500 MHz, $CDCl_3$): δ 9.46 (dd, J = 8.1, 1.3, 1H), 9.18 (d, J = 8.2, 1H), 8.95 (dd, J = 4.3, 1.6, 1H), 8.89 (dd, J = 8.4, 1.3, 1H), 8.41 (d, J = 9.0, 1H), 7.83 (ddd, J = 8.2, 7.0, 1.7, 1H), 7.77 (ddd, J = 7.2, 5.6, 1.2, 1H), 7.64–7.60 (m, 1H), 7.55 (dd, J = 8.3, 4.3, 1H), 7.53 (d, J = 3.1, 1H), 7.29 (d, J = 3.2, 1H), 3.91 (s, 3H); ^{13}C NMR (125 MHz, $CDCl_3$, combined): 147.69, 147.66, 146.9, 145.6, 136.6, 136.3, 134.2, 132.8, 132.7, 131.4, 131.0, 130.1, 129.3, 129.2, 128.8, 128.4, 126.6, 126.4, 126.2, 126.1, 125.8, 125.4, 125.2, 124.6, 124.2, 124.0, 123.9, 123.2, 122.9, 122.8, 122.1, 121.5, 117.7, 117.3, 111.1, 110.6, 104.0, 103.2, 33.3 (2C); IR (film): 3059, 2920, 1739, 1609, 1579, 1513, 1477, 1444, 1418, 1399, 1349, 1290, 1241 cm^{-1}; HRMS-APCI (*m/z*) [M + H$^+$] calcd for $C_{20}H_{15}N_2^+$, 283.12297; found 283.11932.

The structure of **19** was verified by 2D-NOESY and 2D-COSY of the mixture, as the following interactions were observed:

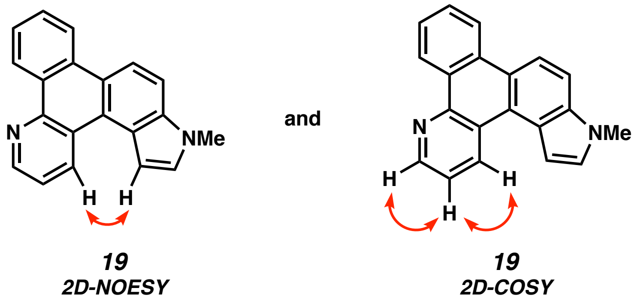

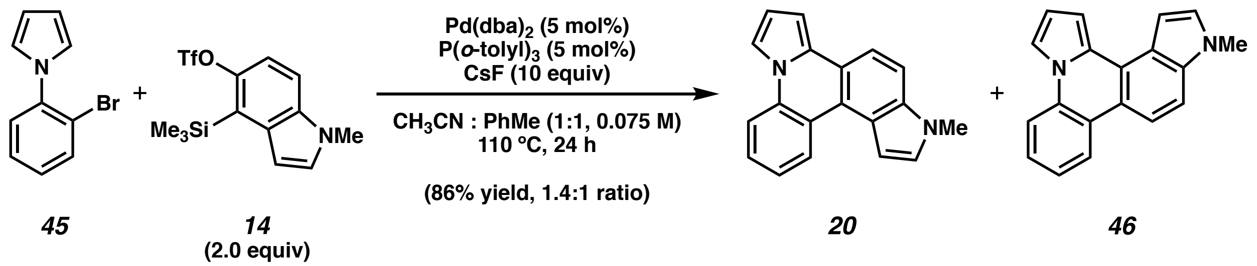

Pyrroles 20 and 46. Followed representative procedure A. Purification by flash chromatography (20:1 Hexanes:Benzene) afforded an inseparable mixture of pyrroles **20** and **46** (86% yield, 1.4:1 ratio, average of two experiments, unassigned) as a yellow solid. Pyrroles **20** and **46**: R_f 0.56 (4:1 Hexanes:EtOAc); ^{1}H NMR (500 MHz, C_6D_6, combined): δ 9.01 (dd, *J* = 8.0, 1.5, 1H), 8.33–8.28 (m, 1H), 8.13 (d, *J* = 8.9, 1H), 8.01 (d, *J* = 8.7, 1H), 7.65 (dd, *J* = 3.0, 1.4, 1H), 7.58 (dd, *J* = 3.0, 1.4, 1H), 7.4 (m, 3H), 7.28 (dd, *J* = 3.2, 0.7, 1H), 7.27–7.23 (m, 2H), 7.22–7.19 (m, 1H), 7.19–7.17 (m, 2H), 7.10 (t, *J* = 0.9, 1H), 7.09 (m, 2H), 6.92 (dd, *J* = 4.0, 2.9, 1H), 6.84 (dd, *J* = 3.9, 2.8, 1H), 6.67 (d, *J* = 3.0, 1H), 6.62 (d, *J* = 3.3, 1H), 2.96 (s, 3H), 2.92 (s, 3H); ^{13}C NMR (125 MHz, C_6D_6, combined): δ 136.7, 136.2, 134.0, 132.9, 131.5, 129.6, 128.9, 128.8, 127.60, 127.58, 127.0, 124.3, 124.2, 124.0, 123.7, 123.5, 123.4, 122.9, 121.5, 120.4, 118.9, 118.6, 117.9, 117.0, 115.3, 115.2, 113.0, 112.9, 112.7, 112.2, 111.5, 108.8, 105.6, 102.94, 102.88, 101.0, 32.22, 32.19; IR (film): 3102, 2923, 1500, 1441, 1355 cm^{-1}; HRMS-APCI (*m/z*) [M + H$^+$] calcd for $C_{19}H_{15}N_2^+$, 271.12297; found 271.12191.

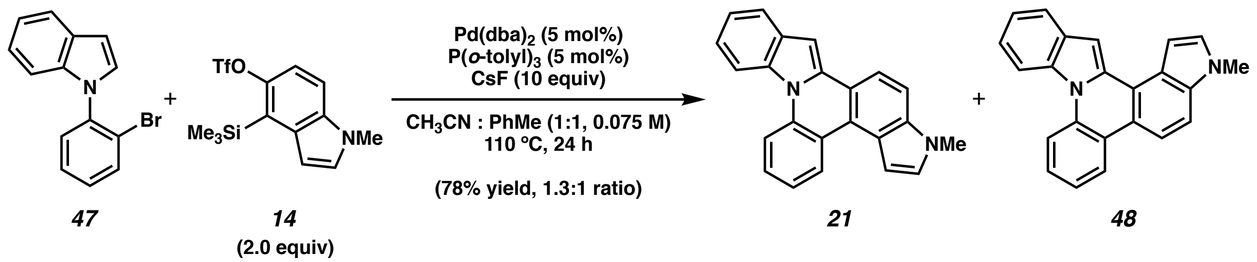

Indoles 21 and 48. Followed representative procedure A. Purification by flash chromatography (100% Hexanes → 100:1 Hexanes:EtOAc → 25:1 Hexanes:EtOAc → 9:1 Hexanes:EtOAc, followed by a second column of 9:1 Hexanes:EtOAc) afforded an inseparable mixture of indoles **21** and **48** (78% yield, 1.3:1 ratio, average of two experiments, unassigned) as a bright yellow solid. Indoles **21** and **48**: R_f 0.32 (4:1 Hexanes:EtOAc); ^{1}H NMR (500 MHz, C_6D_6, major): δ 8.43 (dd, J = 8.2, 1.0, 1H), 8.28 (dd, J = 8.0, 1.1, 2H), 8.04 (d, J = 9.0, 1H), 7.98 (d, J = 8.6, 1H), 7.79 (s, 1H), 7.41 (ddd, J = 7.8, 6.8, 0.9, 1H), 7.36–7.31 (m, 1H), 7.30–7.17 (m, 3H), 7.08 (dd, J = 8.9, 0.6, 1H), 6.63 (d, J = 3.2, 1H), 2.94 (s, 3H); ^{1}H NMR (500 MHz, C_6D_6, minor): δ 8.95 (dd, J = 8.0, 1.6, 1H), 8.41 (dd, J = 8.0, 1.1, 1H), 8.23 (d, J = 8.4, 1H), 7.98 (d, J = 7.6, 1H), 7.91 (d, J = 7.9, 1H), 7.41 (ddd, J = 7.5, 6.9, 0.9, 1H), 7.36–7.31 (m, 1H), 7.30–7.17 (m, 4H), 7.04 (dd, J = 8.7, 0.7, 1H), 6.58 (d, J = 3.2, 1H), 2.92 (s, 3H); ^{13}C NMR (125 MHz, C_6D_6, combined; 44 of 46 signals observed): δ 137.6, 137.2, 136.7, 136.5, 135.6, 135.5, 134.1, 133.9, 131.7, 131.6, 129.3, 129.2, 127.6, 127.3, 124.6, 124.3, 124.0, 123.8, 123.7, 123.04, 122.95, 122.3, 122.11, 122.06, 121.6, 121.5, 121.4, 121.1, 121.0, 120.7, 119.9, 118.7, 116.8, 116.7, 116.6, 114.8, 111.3, 110.5, 103.2, 102.9, 99.8, 95.2, 32.21, 32.18; IR (film): 3040, 2923, 1738, 1601, 1550, 1509, 1490, 1447, 1419, 1355; HRMS-APCI (m/z) $[M + H]^+$ calcd for $C_{23}H_{17}N_2^+$, 321.13862; found 321.13951.

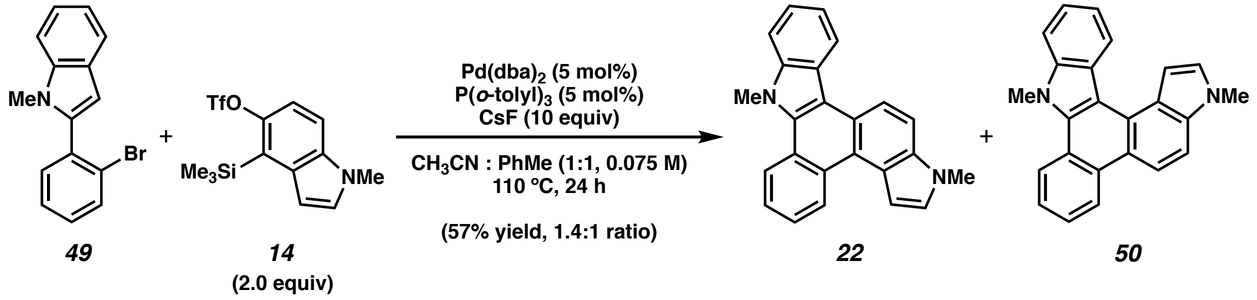

Indoles 22 and 50. Followed representative procedure A. Purification by flash chromatography (100% Hexanes → 200:1 Hexanes:EtOAc → 100:1 Hexanes:EtOAc) afforded an inseparable mixture of indoles **22** and **50** (57% yield, 1.4:1 ratio, average of two experiments) as a yellow

amorphous solid. Indole **22**: R_f 0.40 (3:1 Hexanes:EtOAc); ^{1}H NMR (500 MHz, C_6D_6): δ 9.74 (d, *J* = 8.5, 1H), 9.09 (d, *J* = 8.8, 1H), 8.92–8.88 (m, 1H), 8.48 (dd, *J* = 8.3, 0.9, 1H) 7.70–7.65 (m, 2H), 7.59–7.43 (m, 4H), 7.29 (dd, *J* = 7.5, 1.5, 1H), 6.81 (d, *J* = 3.1, 1H), 3.57 (s, 3H), 3.11 (s, 3H); ^{13}C NMR (125 MHz, C_6D_6; 22 of 24 signals observed): 141.8, 134.8, 134.2, 132.7, 125.7, 125.3, 125.2, 124.4, 123.9, 123.8, 123.6, 123.3, 122.6, 122.1, 120.3, 118.8, 115.7, 111.4, 109.9, 103.9, 34.0, 32.4. Indole **50**: R_f 0.40 (3:1 Hexanes:EtOAc); ^{1}H NMR (500 MHz, C_6D_6): δ 9.37 (d, *J* = 8.1, 1H), 8.92–8.88 (m, 1H), 8.68 (d, *J* = 9.1, 1H), 8.38 (dd, *J* = 8.4, 0.9, 1H), 7.95 (d, *J* = 3.1, 1H), 7.59–7.43 (m, 3H), 7.40 (ddd, *J* = 7.9, 7.1, 1.1, 1H), 7.34 (dd, *J* = 9.1, 0.6, 1H), 7.26 (d, *J* = 8.1, 1H), 6.72 (d, *J* = 3.1, 1H), 3.54 (s, 3H), 3.10 (s, 3H); ^{13}C NMR (125 MHz, C_6D_6; 23 of 24 signals observed): 141.5, 136.5, 136.2, 132.8, 126.3, 125.8, 125.1, 124.8, 124.54, 124.51, 124.46, 123.7, 123.6, 123.1, 121.7, 119.2, 118.3, 115.3, 109.7, 108.1, 105.8, 34.0, 32.4; IR (film, entire mixture): 3055, 2923, 2854, 1737, 1509, 1472, 1374, 1342, 1245, 1102 cm^{-1}; HRMS-APCI (*m/z*) $[M + H]^+$ calcd for $C_{24}H_{19}N_2^+$, 335.15428; found 335.15396.

The structure of **22** was verified by 2D-NOESY of the mixture, as the following interaction was observed:

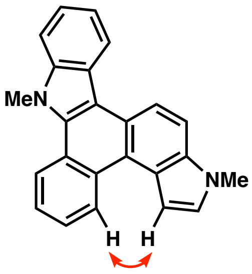

22

2D-NOESY

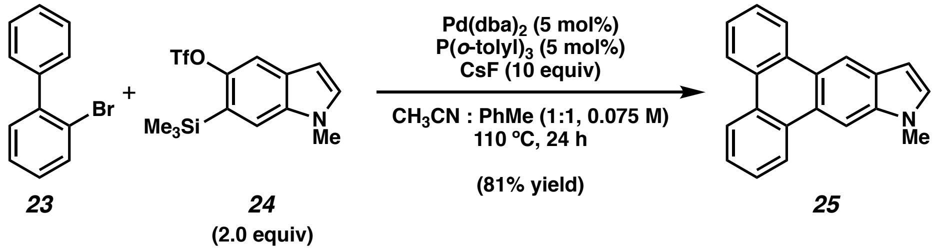

Indole 25. Followed representative Procedure A. Purification by flash chromatography (Hexanes → 1:1 Hexanes:Benzene) afforded indole **25** (81% yield, average of two experiments) as an off-white solid. Indole **25**: mp 169.6–172.0 °C; R_f 0.25 (9:1 Hexanes:EtOAc); ^{1}H NMR (500 MHz, $CDCl_3$): δ 8.92 (s, 1H), 8.73 (d, *J* = 8.3, 2H), 8.62 (t, *J* = 7.7, 2H), 8.51 (s, 1H), 7.66–7.56 (m, 4H),

7.27 (d, *J* = 2.6, 1H), 6.70 (dd, *J* = 0.8, 3.1, 1H), 3.99 (s, 3H); ^{13}C NMR (125 MHz, $CDCl_3$; 27 of 28 signals observed): δ 137.4, 132.0, 131.4, 131.2, 129.6, 129.3, 129.0, 127.3, 127.1, 126.6, 126.1, 125.7, 123.56, 123.55, 123.4, 123.22, 123.15, 115.1, 102.3, 101.1, 33.2; IR (film): 3081, 2928, 2811, 1628, 1601, 1520, 1446, 1218, 1085, 754 cm^{-1}; HRMS–APCI (m/z) $[M]^+$ calcd for $C_{21}H_{16}N^+$, 281.11990; found 281.12065.

B. Synthesis of Silyl Triflate Precursor to 2,3-Carbazolyne

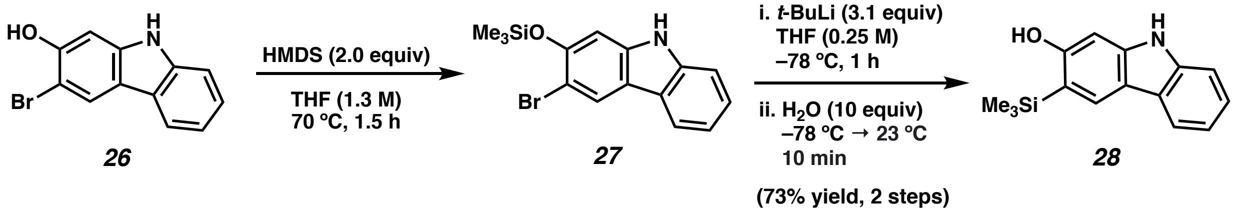

Silyl alcohol 28. A 20 mL scintillation vial was charged with 3-bromo-2-hydroxycarbazole (**26**, 4.3 g, 16 mmol, 1.0 equiv) and THF (5.6 mL, 1.3 M). HMDS (6.9 mL, 2.0 equiv, 33 mmol) was added in one portion. The vial was sealed with a Teflon cap, and subsequently placed in an aluminum block preheated to 70 °C, where it was allowed to stir for 1.5 h. After stirring for 1.5 h, the reaction mixture was cooled to 23 °C and concentrated under reduced pressure to afford the intermediate silyl enol ether **27** as a pink solid. This was carried forward without further purification.

The crude solid was dissolved in THF (8.5 mL, 0.25 M) and purged with nitrogen for 3 minutes, before being cooled to –78 °C. *tert*-Butyllithium (1.70 M, 4.17 mL, 7.09 mmol, 3.1 equiv) was then added dropwise over 10 min. The solution was allowed to stir for 1 hour at –78 °C. After the allotted time, deionized H_2O (408 μL, 22.5 mmol, 10 equiv) was added dropwise over 2 min. The solution was then allowed to warm to 23 °C over 10 min and the mixture was transferred to a separatory funnel with H_2O (20 mL) and CH_2Cl_2 (20 mL). The layers were then separated and the aqueous layer was extracted with CH_2Cl_2 (2 x 20 mL). The combined organic layers were dried over $MgSO_4$, filtered, and concentrated under reduced pressure. The crude residue was then purified by flash chromatography (100% Benzene) to afford silyl alcohol **28** as a white solid (3.1 g, 73% yield over two steps). Silyl alcohol **28**: mp: >200 °C; R_f 0.15 (100% Benzene); ^{1}H NMR (600 MHz, $CDCl_3$): δ 8.03 (s, 1H), 8.00 (d, *J* = 7.7, 1H), 7.84 (s, 1H), 7.36–7.32 (m, 2H), 7.21 (dd, J = 6.6, 2.2, 1H), 6.72 (d, J= 2.4, 1H), 4.94 (s, 1H), 0.39 (s, 9H); ^{13}C NMR (125 MHz, $CDCl_3$): δ

159.4, 141.8, 139.2, 126.9, 124.4, 123.4, 119.6, 119.3, 117.5, 117.2, 110.2, 96.0, –0.7; IR (film): 3416, 3016, 2970, 2926, 2854, 1738, 1366 cm^{-1}; HRMS-APCI (*m/z*) $[M + H]^+$ calcd for $C_{13}H_{18}NOSi^+$, 256.1152; found 256.1171.

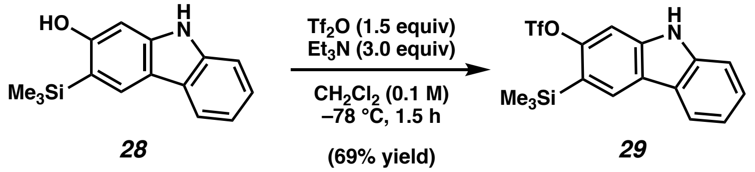

Silyl triflate 29. Silyl alcohol **28** (403 mg, 1.58 mmol, 1.00 equiv) was suspended in CH_2Cl_2 (15 mL). The solution was purged with nitrogen for 3 minutes, then cooled to –78 °C. Triethylamine (0.66 mL, 4.7 mmol, 3.0 equiv) was then added in one portion followed by trifluoromethanesulfonic anhydride (400 µL, 2.37 mmol, 1.5 equiv), which was added dropwise over 5 min. The solution was allowed to stir for 1.5 h at –78 °C. After the allotted time, saturated aqueous $NaHCO_3$ (20 mL) was added over 1 minute. The solution was then allowed to warm to 23 °C over 10 min and the mixture was transferred to a separatory funnel with H_2O (10 mL) and CH_2Cl_2 (20 mL). The layers were then separated and the aqueous layer was extracted with CH_2Cl_2 (2 x 20 mL). The combined organic layers were dried over $MgSO_4$, filtered, and concentrated under reduced pressure. The crude residue was then purified by flash chromatography (100% Benzene) to afford silyl triflate **29** as a clear oil (375 mg, 69% yield). Silyl triflate **29**: R_f = 0.59 (100% Benzene); ^1H NMR (500 MHz, $CDCl_3$): δ 8.22 (s, 1H), 8.16 (s, 1H), 8.08 (d, J = 7.9, 1H), 7.46 – 7.44 (m, 3H), 7.28 (ddd, J = 7.8, 5.0, 3.1, 1H), 0.44 (s, 9H); ^{13}C NMR (125 MHz, $CDCl_3$): δ 153.3, 140.4, 140.2, 127.5, 126.6, 122.7, 122.3, 121.9, 120.43, 120.41, 110.9, 102.0, 0.40; IR (film): 3443, 3016, 2970, 2948, 1739, 1217 cm^{-1}; HRMS-APCI (*m/z*) $[M + H]^+$ calcd for $C_{16}H_{17}NO_3F_3SSi^+$, 388.0645; found 388.0659.

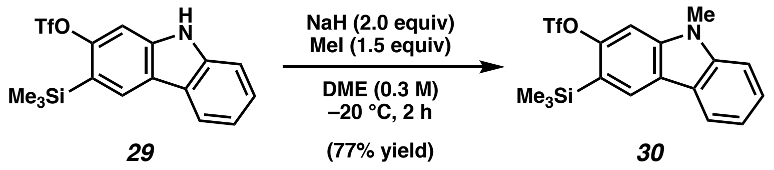

***N*-Me-Carbazole Silyl Triflate 30**. To a 2-dram vial was added carbazole silyl triflate **29** (196 mg, 0.506 mmol, 1.00 equiv) and DME (1.7 mL, 0.3 M). The resulting mixture was stirred under positive nitrogen pressure. The mixture was cooled to –20 °C in a dry ice and water/methanol (7:3)

bath for 5 min. To the cooled solution was added iodomethane (48 µL, 0.76 mmol, 1.5 equiv) in a single portion. Next, the septum was removed and NaH (60% dispersion in mineral oil, 40.5 mg, 1.01 mmol, 2.0 equiv) was added in a single portion, then the septum was quickly replaced. The reaction continued to stir under nitrogen at –20 °C for 2 h, at which point the reaction was quenched with sat. aq. NH_4Cl (5 mL) and allowed to stir at 23 °C for 5 min. The mixture was transferred to a separatory funnel containing water (10 mL) and EtOAc (10 mL). The layers were separated and the aqueous layer was extracted with EtOAc (3 x 10 mL). The organic layers were combined, dried over $MgSO_4$, filtered, and concentrated under reduced pressure to afford a yellow solid. The crude residue was purified by flash chromatography (16:1 Hexanes:Benzene) to afford *N*-Me-carbazole silyl triflate **30** (156 mg, 77% yield) as a white solid. *N*-Me-carbazole silyl triflate **30**: mp 128.2–130.3 °C; R_f 0.79 (9:1 Hexanes:EtOAc); ^{1}H NMR (600 MHz, $CDCl_3$): δ 8.18 (s, 1H), 8.11 (d, *J* = 7.9, 1H), 7.53–7.50 (m, 1H), 7.43 (d, *J* = 8.3, 1H), 7.38 (s, 1H), 7.30 (td, *J* = 7.5, 0.9, 1H), 3.85 (s, 3H), 0.44 (s, 9H); ^{13}C NMR (125 MHz, $CDCl_3$; 14 of 15 signals observed): δ 153.5, 142.1, 141.9, 127.4, 126.4, 122.1, 121.9, 121.0, 120.4, 119.9, 108.8, 100.0, 29.3, –0.4; IR (film): 3017, 2955, 1739, 1596, 1414, 1206 cm^{-1}; HRMS-APCI (*m/z*) [M + H]$^+$ calcd for $C_{17}H_{19}F_3NO_3SSi^+$, 402.0802; found 402.0690.

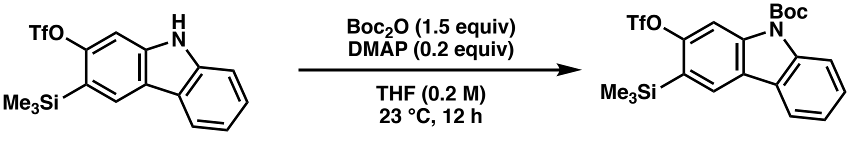

29 *31*

***N*-Boc-Carbazole Silyl Triflate 31**. To a 50 mL round bottom flask was added carbazole silyl triflate **29** (1.00 g, 2.58 mmol, 1.0 equiv), THF (13 mL, 0.2 M), 4-dimethylaminopyridine (63 mg, 0.51 mmol, 0.2 equiv), and di-*tert*-butyl dicarbonate (839 mg, 3.84 mmol, 1.5 equiv). The resulting mixture was purged with nitrogen for 3 minutes, then allowed to stir under positive nitrogen pressure at 23 °C for 2 h. The mixture was then quenched with deionized water (10 mL). The solution was transferred to a separatory funnel and the layers were separated. The aqueous layer was subsequently extracted with CH_2Cl_2 (3 x 10 mL). The organic layers were combined, dried over Na_2SO_4, filtered, and concentrated under reduced pressure to afford a crude, yellow solid. The crude material was purified by flash chromatography (100:1 Hexanes:EtOAc) to yield *N*-Boc-carbazole silyl triflate **31** (1.17 g, 93% yield) as a white solid. *N*-Boc-carbazole silyl triflate **31**: mp: 170.5–172.3 °C; R_f 0.57 (9:1 Hexanes:EtOAc); ^{1}H NMR (600 MHz, $CDCl_3$): δ 8.36 (d, *J* =

8.6, 1H), 8.35 (s, 1H), 8.08 (s, 1H), 8.00 (d, *J* = 7.9, 1H), 7.51 (td, *J* = 7.6, 1.0, 1H), 7.39 (td, *J* = 7.5, 1.0, 1H), 1.76 (s, 9H), 0.44 (s, 9H); ^{13}C NMR (125 MHz, $CDCl_3$; 16 of 17 signals observed): δ 153.9, 150.6, 139.6, 127.8, 126.6, 126.4, 124.9, 124.5, 123.6, 119.9, 119.8, 116.5, 108.3, 85.1, 28.4, –0.5; IR (film): 2983, 1728, 1393, 1355, 1212, 1155, 1140 cm^{-1}; HRMS-APCI (*m/z*) [M + H]$^+$ calcd for $C_{21}H_{25}F_3NO_5SSi^+$, 488.1169; found 488.1198.

C. Annulation of *N*-Me-Carbazolyne

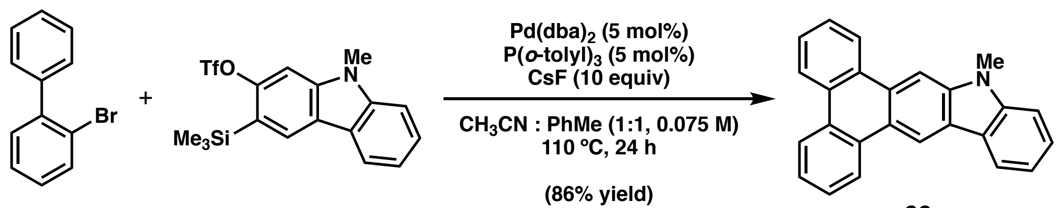

23 **30** (2.0 equiv) **32**

Carbazole 32. Followed representative procedure A. Purification by flash chromatography (100% Hexanes → 1:1 Hexanes:Benzene) afforded carbazole **32** (86% yield) as an off-white solid. Carbazole **32**: mp >200 °C; R_f 0.74 (4:1 Hexanes:EtOAc); ^{1}H NMR (400 MHz, $CDCl_3$): δ 9.38 (s, 1H), 8.86 (d, *J* = 8.2, 1H), 8.81 (d, *J* = 7.8, 1H), 8.68 (td, *J* = 8.6, 1.5, 2H), 8.54 (s, 1H), 8.31 (dt, *J* = 7.8, 0.9, 1H), 7.72–7.60 (m, 4H), 7.63 (td, *J* = 7.8, 1.1, 1H), 7.58 (td, *J* = 7.8, 1.1, 1H), 7.47 (d, *J* = 8.1, 1H), 7.33 (td, *J* = 7.4, 0.8, 1H), 4.03 (s, 3H); ^{13}C NMR (100 MHz, $CDCl_3$; 27 of 28 signals observed): δ 142.9, 141.3, 131.1, 130.6, 130.0, 128.85, 128.78, 127.4, 127.1, 127.0, 126.8, 126.1, 123.9, 123.6, 123.5, 123.4, 123.1, 123.0, 122.9, 120.8, 119.2, 114.8, 108.5, 101.1, 29.3; IR (film): 3049, 2923, 2854, 1638, 1603, 1500, 1443, 1258, 754 cm^{-1}; HRMS-APCI (m/z) [M]$^+$ calcd for $C_{25}H_{17}N^+$, 331.13555; found 331.13609.

D. Synthesis of N–H Annulation Products for Metal Coordination

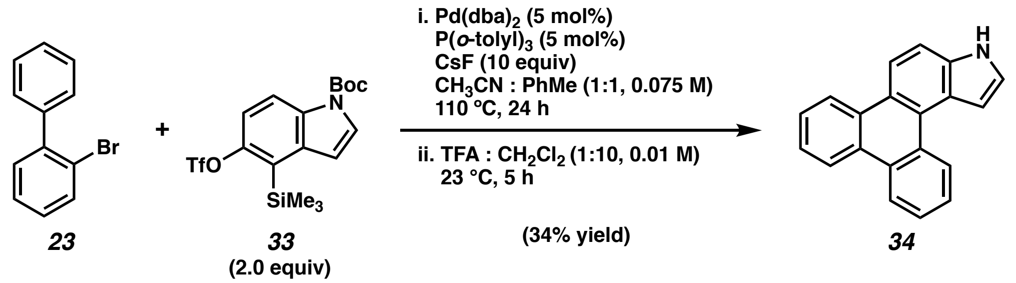

Indole 34. A 2-dram vial was charged with Pd(dba)$_2$ (7.0 mg, 0.012 mmol, 5 mol%). Next, toluene (1.5 mL), P(*o*-tolyl)$_3$ (3.7 mg, 0.012 mmol, 5 mol%), 2-bromobiphenyl (**23**) (56.4 mg, 0.242 mmol, 1.0 equiv), silyl triflate **33** (318 mg, 0.495 mmol, 2.0 equiv), and acetonitrile (1.5 mL) were added sequentially, followed by an oven-dried magnetic stirbar and then CsF (333 mg, 8.26 mmol, 10.0 equiv). The vial was purged with nitrogen for 3 minutes, then sealed with a Teflon-lined screw cap and stirred at 110 °C for 24 h. After allowing to cool to 23 °C, the mixture was transferred with CH_2Cl_2 (20 mL) and H_2O (10 mL) to a separatory funnel containing brine (15 mL). The layers were separated and the aqueous layer was extracted with CH_2Cl_2 (3 x 30 mL). The combined organic layers were dried over Na_2SO_4, filtered, and concentrated under reduced pressure to afford a brown residue that was carried forward without further purification.

The crude material was dissolved in 10:1 CH_2Cl_2:TFA (24.2 mL, 0.01 M) and stirred at 23 °C for 5 h. The reaction was then slowly transferred to a separatory funnel containing sat. aq. sodium bicarbonate (30 mL). The mixture was further diluted with CH_2Cl_2 (10 mL) and the layers were separated. The organic phase was dried over Na_2SO_4 and filtered. To the filtrate was added silica (500 mg). The resulting mixture was dried under reduced pressure until a free-flowing solid was obtained. The crude material purified by flash chromatography (2:3 CH_2Cl_2:Hexanes → 3:1 CH_2Cl_2:Hexanes) to afford indole **34** (22.0 mg, 34% yield) as a yellow solid. Indole **34**. mp: >200 °C; R$_f$ 0.22 (2:3 CH_2Cl_2:Hexanes); ^{1}H NMR (600 MHz, DMSO-d$_6$): δ 9.23 (dd, *J* = 8.6, 1.4, 1H), 8.77 (dd, *J* = 8.3, 1.7, 1H), 8.74–8.79 (m, 2H), 8.55 (d, *J* = 8.9, 1H), 8.50 (s, 1H), 7.76–7.60 (m, 6H), 7.41 (t, *J* = 3.6, 2.8, 1H); ^{13}C NMR (100 MHz, $CDCl_3$): δ 135.5, 131.1, 131.0, 130.3, 128.9, 127.1, 127.0, 126.7, 126.3, 125.9, 124.5, 124.4, 124.1, 123.5, 123.19, 123.18, 123.13, 118.1, 112.3, 105.6; IR (film): 3416, 3026, 3073, 2956, 1725, 1350 cm^{-1}; HRMS-APCI (*m/z*) [M + H]$^+$ calcd for $C_{20}H_{14}N^+$, 268.1121; found 267.6527.

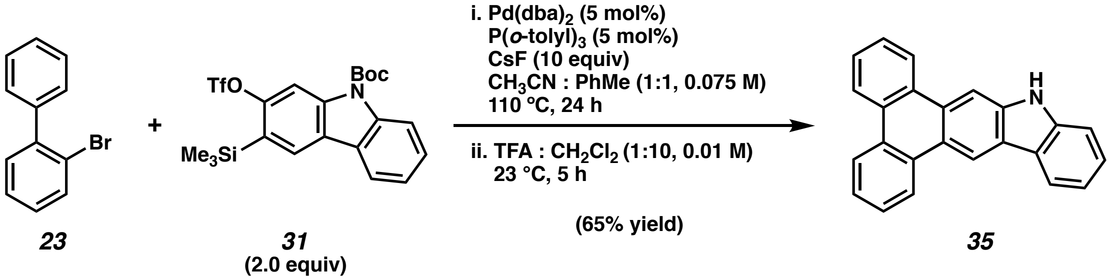

23　　*31* (2.0 equiv)　　*35*

Carbazole 35. A 2-dram vial was charged with $Pd(dba)_2$ (7.1 mg, 0.012 mmol, 5 mol%). Next, toluene (1.3 mL), P(*o*-tolyl)$_3$ (3.8 mg, 0.012 mmol, 5 mol%), 2-bromobiphenyl (**23**) (58.0 mg, 0.248 mmol, 1.0 equiv), silyl triflate **31** (241 mg, 0.495 mmol, 2.0 equiv), and acetonitrile (1.6 mL) were added, followed by an oven-dried magnetic stirbar and then CsF (376 mg, 2.48 mmol, 10 equiv). The vial was purged with nitrogen for 3 minutes, then sealed with a Teflon-lined screw cap and stirred at 110 °C for 24 h. After allowing to cool to 23 °C, the mixture was transferred with CH_2Cl_2 (20 mL) and H_2O (10 mL) to a separatory funnel containing brine (15 mL). The layers were separated and the aqueous layer was extracted with CH_2Cl_2 (3 x 30 mL). The combined organic layers were dried over Na_2SO_4, filtered, and concentrated under reduced pressure to afford a brown residue that was carried forward without further purification.

The crude material was dissolved in 10:1 CH_2Cl_2:TFA (24.2 mL, 0.01 M) and stirred at 23 °C for 5 h. The reaction was then slowly transferred to a separatory funnel containing sat. aq. sodium bicarbonate (30 mL). The mixture was further diluted with CH_2Cl_2 (10 mL) and the layers were separated. The organic phase was dried over Na_2SO_4 and filtered. To the filtrate was added silica (500 mg). The resulting mixture was dried under reduced pressure until a free-flowing solid was obtained. The crude material purified by flash chromatography (100% Hexanes → 9:1 Hexanes:EtOAc → 1:1 Hexanes:Benzene) to yield carbazole **35** (51 mg, 65% yield) as an off-white solid. Carbazole **35**. mp >200 °C; R_f 0.63 (4:1 Hexanes:EtOAc); ^{1}H NMR (600 MHz, $CDCl_3$): δ 11.35 (s, 1H), 9.62 (s, 1H), 9.0 (d, J = 8.0, 1H), 8.82 (d, J = 8.3, 1H), 8.76 (dd, J = 12.5, 8.3, 2H), 8.72 (s, 1H), 8.43 (d, J = 7.5, 1H), 7.73–7.69 (m, 2H), 7.67 (t, J = 6.9, 1H), 7.62 (t, J = 7.5, 1H), 7.55 (d, J = 8.0, 1H), 7.46 (t, J = 7.5, 1H), 7.24 (t, J = 7.5, 1H); ^{13}C NMR (100 MHz, $CDCl_3$; 22 of 24 signals observed): δ 141.3, 139.8, 131.0, 130.3, 129.9, 129.0, 128.8, 127.3, 127.1, 127.0, 126.9, 126.2, 124.4, 123.5, 123.43, 123.36, 123.0, 120.8, 119.8, 114.8, 110.6, 103.4; IR (film): 3413, 2923, 2852, 1611, 1435, 751 cm^{-1}; HRMS-APCI (*m/z*) $[M + H]^+$ calcd for $C_{24}H_{16}N^+$, 318.12773; found 318.12843.

E. Synthesis of Two-Coordinate Metal Complexes

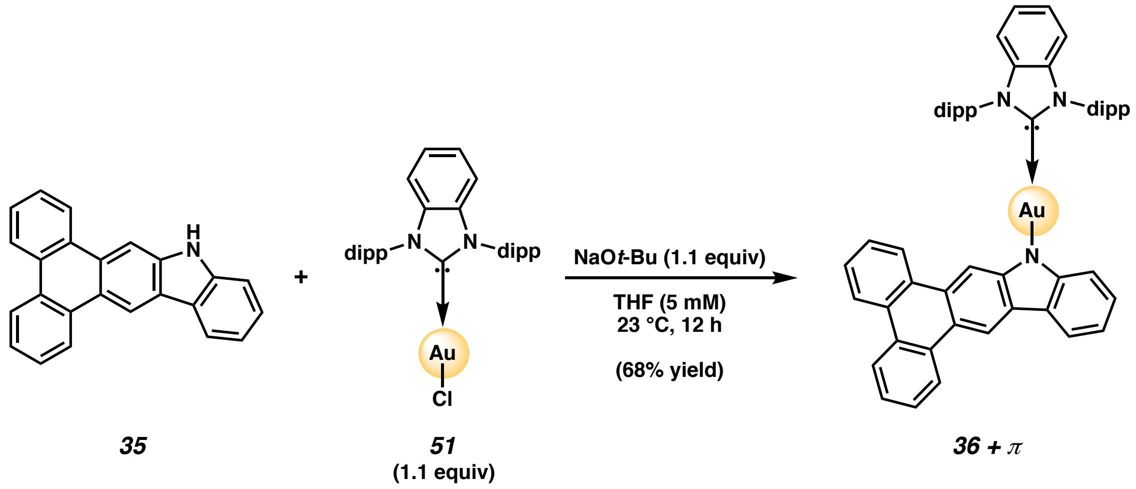

Representative Procedure B for metal coordination (Figure 7, complex 36+π used as an example). Sodium *tert*-butoxide (6.4 mg, 67 µmol, 1.1 equiv) was added to a solution of ligand **35** (20 mg, 63 µmol, 1.0 equiv) in THF (10 mL). **51** (44.4 mg, 66.2 µmol, 1.1 equiv) was added to the reaction flask in one portion and the mixture was left stirring under inert gas at 23 ºC for 12 hours. The solution was filtered through a plug of celite (2 cm in a 15 mL fritted funnel), washed with THF (10 mL), and the volatiles were removed under reduced pressure. The resulting product was recrystallized from CH_2Cl_2 and hexanes to afford an off-white precipitate (47 mg, 68% yield). Carbazole–Au–BZI **36+π**: ^{1}H NMR (400 MHz, $CDCl_3$): δ 9.25 (s, 1H), 8.77 (dd, *J* = 8.5, 1.3, 1H), 8.60 (ddd, *J* = 16.7, 8.4, 1.4, 2H), 8.28 (dd, *J* = 8.5, 1.4, 1H), 8.17 (dd, *J* = 7.6, 1.0, 1H), 8.13 (s, 1H), 7.86 (t, *J* = 7.8, 2H), 7.72 (ddd, *J* = 8.2, 6.9, 1.3, 1H), 7.65–7.56 (m, 6H), 7.52–7.47 (m, 3H), 7.29–7.26 (m, 2H), 7.16 (ddd, *J* = 8.2, 7.0, 1.3, 1H), 7.01 (ddd, *J* = 7.9, 7.0, 1.0, 1H), 6.67 (dd, *J* = 8.1, 0.9, 1H), 2.60 (hept, *J* = 6.9, 4H), 1.40 (d, *J* = 6.9, 12H), 1.19 (d, *J* = 6.8, 12H). ^{13}C NMR (151 MHz, $CDCl_3$): δ 207.21, 206.89, 185.66, 151.68, 149.68, 147.17, 146.43, 134.96, 132.19, 131.63, 131.44, 131.04, 129.51, 128.02, 127.23, 126.88, 126.05, 125.74, 125.54, 125.33, 124.75, 124.64, 123.80, 123.73, 123.17, 123.07, 122.78, 120.55, 119.85, 116.11, 113.50, 111.90, 105.58, 30.89, 29.19, 24.64, 24.08. Anal. Calcd for $C_{55}H_{52}AuN_3$: C, 69.39; N, 4.41; H, 5.51. Found: C, 68.05; N, 4.27; H, 5.30. MALDI-TOF (*m/z*) $[M]^+$ calcd for $C_{55}H_{52}AuN_3^+$, 951.38; found, 951.22.

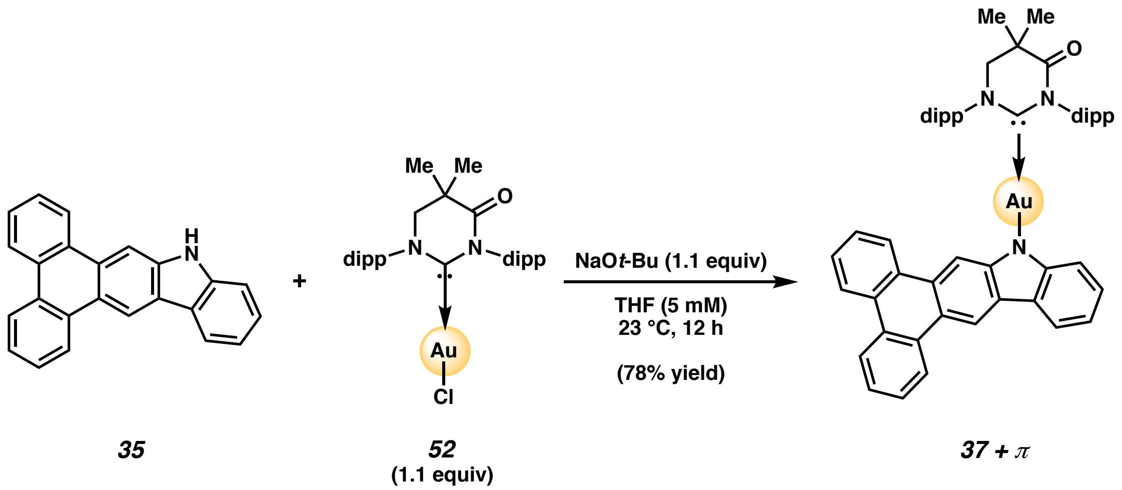

Annulated Carbazole–Au–MAC 37+π. Representative procedure B was followed to yield **37+π** (42 mg, 78% yield) as a yellow solid. Annulated Carbazole–Au–MAC **37+π**: ^{1}H NMR (400 MHz, $CDCl_3$): δ 9.17 (s, 1H), 8.73 (d, *J* = 7.8 Hz, 1H), 8.57 (ddd, *J* = 18.5, 8.3, 1.1 Hz, 2H), 8.38 (d, *J* = 8.1 Hz, 1H), 8.08 (d, *J* = 0.7 Hz, 1H), 7.82 – 7.70 (m, 3H), 7.65 – 7.44 (m, 8H), 7.04 – 6.91 (m, 2H), 5.81 (d, *J* = 7.6 Hz, 1H), 3.86 (s, 2H), 3.36 (hept, *J* = 6.5 Hz, 2H), 3.08 (hept, *J* = 6.8 Hz, 2H), 1.56 (s, 6H), 1.45 – 1.33 (m, 18H), 1.26 (d, *J* = 6.8 Hz, 6H). ^{13}C NMR (101 MHz, $CDCl_3$): δ 204.87, 171.43, 151.72, 149.54, 146.05, 144.95, 140.12, 135.57, 132.07, 131.35, 130.60, 130.44, 129.45, 127.98, 127.33, 126.87, 125.86, 125.79, 125.65, 125.63, 124.82, 124.73, 124.54, 124.16, 123.54, 123.14, 123.02, 122.80, 120.63, 119.41, 116.24, 114.06, 113.35, 105.48, 77.19, 62.27, 37.95, 29.35, 28.98, 24.70, 24.61, 24.58, 24.13, 23.93. Anal. Calcd for $C_{54}H_{56}AuN_3O$: C, 67.56; N, 4.38; H, 5.88. Found: C, 67.41; N, 4.35; H, 5.93. MALDI-TOF (*m/z*) $[M]^+$ calcd for $C_{54}H_{56}AuN_3O^+$, 959.41; found, 959.16.

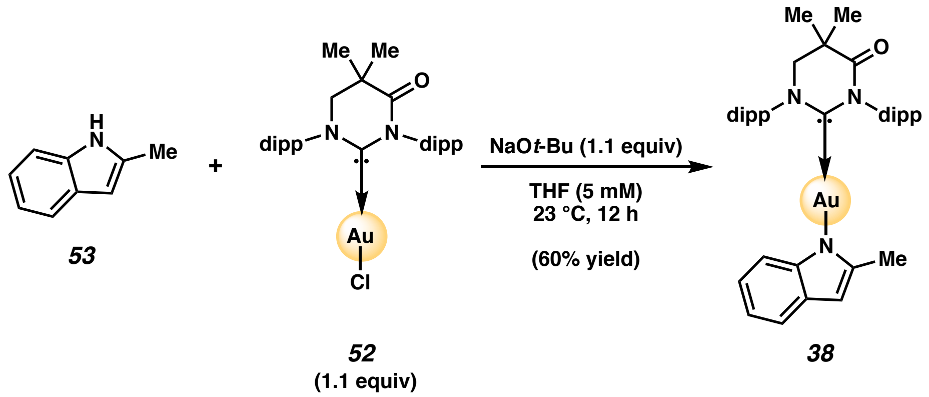

2-Methyl Indole–Au–MAC 38. Representative procedure B was followed to yield **38** (110 mg, 60% yield) as an off-white solid. 2-Methylindole–Au–MAC **38**: ^{1}H NMR (400 MHz, acetone-D$_6$): δ 7.64 (dt, *J* = 17.7, 7.7, 2H), 7.52 (d, *J* = 7.8, 2H), 7.46 (d, *J* = 7.7, 2H), 7.09 (dd, *J* = 7.6, 1.2, 1H), 6.62–6.53 (m, 1H), 6.49 (td, *J* = 7.5, 1.3, 1H), 5.84 (d, *J* = 8.0, 1H), 5.74 (d, *J* = 1.0, 1H), 4.25 (s, 2H), 3.50 (hept, *J* = 6.8, 2H), 3.24 (hept, *J* = 6.8, 2H), 1.69 (s, 6H), 1.48–1.43 (m, 8H), 1.42 (d, *J* = 3.2, 7H), 1.40 (d, *J* = 6.8, 6H), 1.25 (d, *J* = 6.8, 6H). ^{13}C NMR (101 MHz, acetone-D$_6$): δ 205.18, 171.91, 146.16, 145.94, 144.96, 144.43, 140.69, 136.56, 130.54, 130.07, 129.77, 125.29, 124.30, 117.03, 116.77, 116.13, 113.55, 98.29, 61.12, 37.98, 28.49, 23.94, 23.70, 23.65, 23.25, 15.60. Anal. Calcd for $C_{39}H_{50}AuN_3O$: C, 60.54; N, 5.43; H, 6.51. Found: C, 60.49; N, 5.32; H, 6.67. MALDI-TOF (*m/z*) [M]$^+$ calcd for $C_{39}H_{50}AuN_3O^+$, 773.36; found, 773.45.

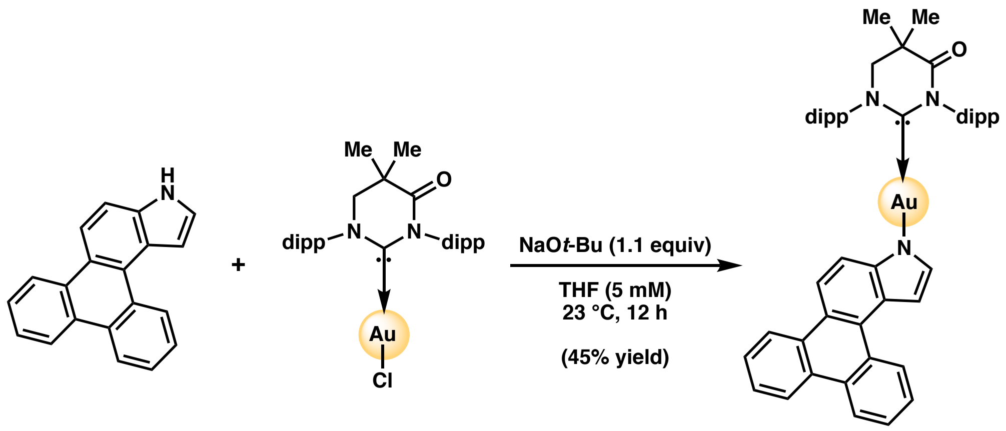

34 ***52*** (1.1 equiv) ***38 + π***

Annulated Indole–Au–MAC 38+π. Representative procedure B was followed to yield **38** (120 mg, 45% yield) as an off-white solid. Annulated Indole–Au–MAC **38+π**: ^{1}H NMR (400 MHz, acetone-D$_6$): δ 9.18 – 9.11 (m, 1H), 8.77 – 8.62 (m, 3H), 8.05 (d, *J* = 9.1 Hz, 1H), 7.72 (dt, *J* = 20.8, 7.8 Hz, 2H), 7.65 – 7.45 (m, 8H), 7.16 – 7.10 (m, 1H), 6.52 (d, *J* = 2.8 Hz, 1H), 6.49 (dd, *J* = 8.9, 1.0 Hz, 1H), 4.29 (s, 2H), 3.48 (h, *J* = 6.8 Hz, 2H), 3.24 (hept, *J* = 6.8 Hz, 2H), 1.68 (s, 6H), 1.50 – 1.31 (m, 18H), 1.22 (d, *J* = 6.8 Hz, 6H). ^{13}C NMR (151 MHz, acetone-D$_6$): δ 205.19, 203.69, 171.81, 146.27, 145.07, 145.00, 140.59, 136.57, 135.92, 132.09, 131.87, 130.31, 129.95, 129.45, 128.12, 126.78, 126.61, 126.17, 125.36, 125.10, 124.71, 124.37, 124.18, 124.05, 123.32, 123.20, 122.94, 122.74, 122.49, 117.26, 113.45, 103.02, 60.96, 38.13, 28.85, 28.35, 24.04, 23.97, 23.92, 23.86, 23.79, 23.72, 23.53, 23.21, 23.07. Anal. Calcd for $C_{50}H_{54}AuN_3O$: C, 66.00; N, 4.62; H,

5.98. Found: C, 65.50; N, 4.49; H, 6.09. MALDI-TOF (*m/z*) $[M]^+$ calcd for $C_{50}H_{54}AuN_3O^+$, 909.39; found, 909.27.

F. General Procedure for Photophysical Property Analyses

General Procedure for 1% Polystyrene Films. A mixture of polystyrene pellets (99 mg) and toluene (2 mL) was sonicated for 1 h, until all pellets are dissolved. Two-coordinate metal complex (1 mg) was dissolved in the solution. Using a pipet, ~0.5 mL of the solution was drop-casted onto a glass substrate (2 cm x 2 cm) to achieve an even surface. The film was left to air-dry for 30 min and then placed in the vacuum chamber for further drying overnight. The resulting film was used to acquire the photophysical data.

G. Absorption Spectra of Donor Ligands

The extinction coefficient for the donor ligands was measured in 2-MeTHF. The molar absorptivity values for the donor ligands are comparable in magnitude to the molar absorptivity values for the transitions localized on the donor ligands in the metal complexes.

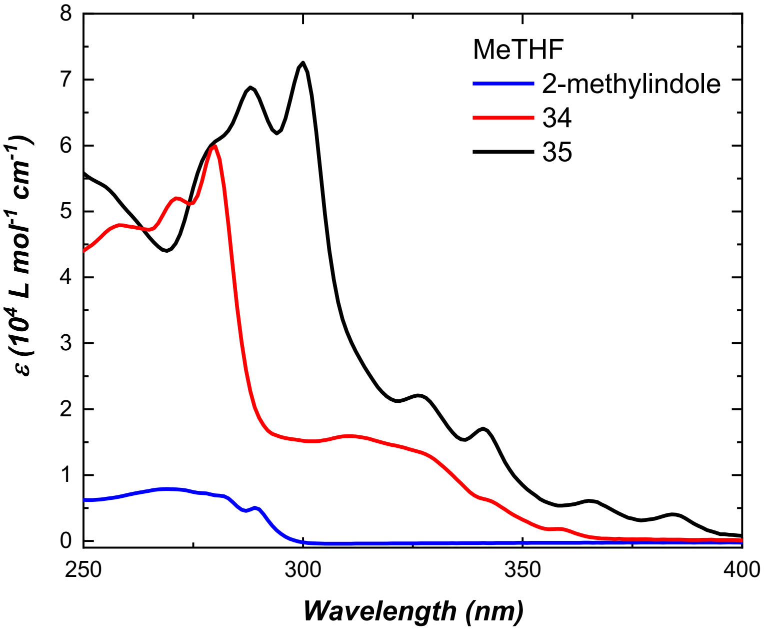

Supplementary Figure 1. Extinction coefficients for the donor ligands in 2-MeTHF.

H. Emission Spectra of Deprotonated Donor Ligands

A few milligrams of donor ligand (1–3 mg) were dissolved in THF (10 mL). The solution was cooled in an ice bath. Once completely dissolved, a solution containing 1.2 equivalents of *n*-BuLi diluted in THF was added. Although a color change was immediately observable, the reaction was

allowed to stir for 1 h. The solvent was then evaporated under reduced pressure, and the solid obtained was dissolved in 2-MeTHF.

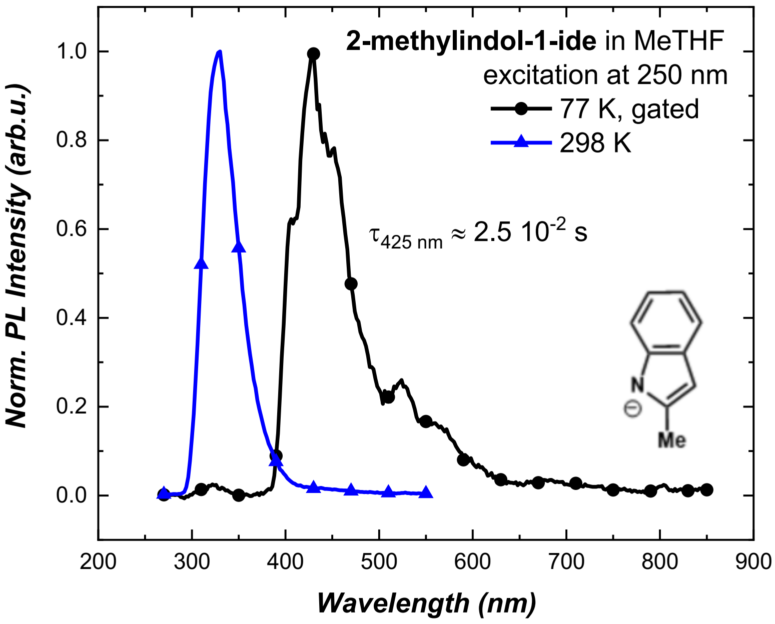

Supplementary Figure 2. Emission spectra of 2-methylindol-1-ide in 2-MeTHF.

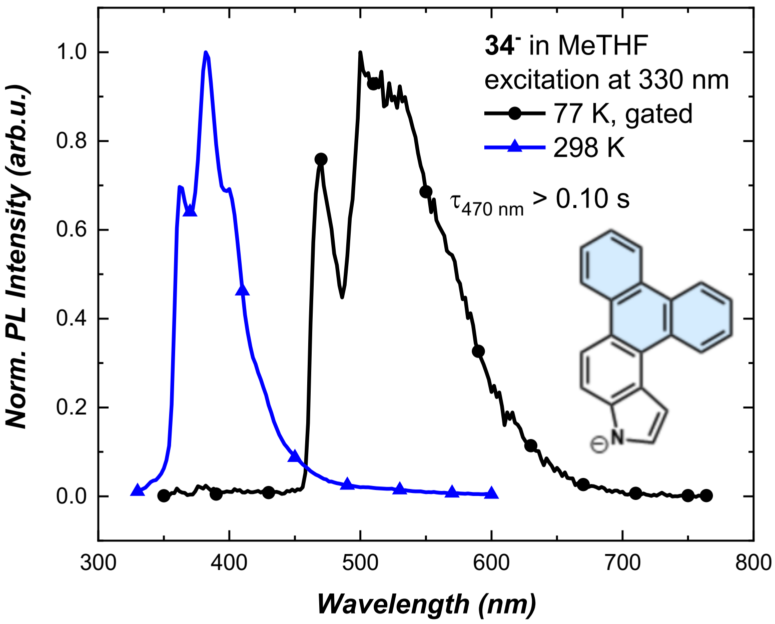

Supplementary Figure 3. Emission spectra of ligand **34**$^-$ in 2-MeTHF.

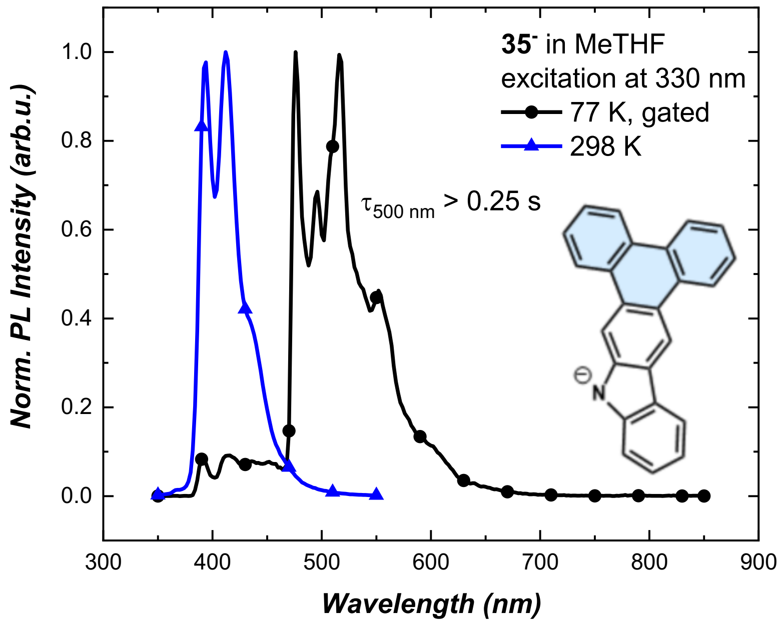

Supplementary Figure 4. Emission spectra of ligand **35**$^-$ in 2-MeTHF.

Computational Methods

A. Complete Citation of Q-Chem 5.1

Density functional theory (DFT) calculations were executed using Q-Chem 5.1 program[11] at the B3LYP/6-31G** level for ground state geometry optimization of the organic ligands and at the B3LYP/LACVP level for the metal complexes. Time-dependent density functional theory (TD-DFT) calculations were performed on the ground state optimized geometries at the CAM-B3LYP/LACVP** level for a balanced description of both charge-transfer and locally excited (LE) states (Supplementary Table 1).

B. Calculated Frontier Molecular Orbital Energies

Supplementary Table 1. Calculated frontier molecular orbital, singlet (S_1), and triplet (T_1) energies. CT = Charge Transfer state, LE = Locally Excited state.

	HOMO (eV)	LUMO (eV)	$\Delta E_{HOMO-LUMO}$ (eV)	S_1 (eV)	Nature of S_1 transition	T_1 (eV)	Nature of T_1 transition
**2-methylindole**	-5.28	0.00	5.28	5.21	-	3.16	-
**34**	-5.28	-0.90	4.38	4.24	-	2.55	-
**carbazole**	-5.47	-0.65	4.82	4.58	-	3.09	-
**35**	-5.28	-1.20	4.08	4.04	-	2.53	-
**36**	-4.22	-1.44	2.78	3.50	CT	3.07	LE
**36+π**	-4.27	-1.52	2.75	3.46	CT	2.48	LE
**37**	-4.30	-1.96	2.34	3.10	CT	2.85	CT
**37+π**	-4.35	-2.07	2.29	3.07	CT	2.48	LE
**38**	-4.16	-1.96	2.20	3.11	CT	2.87	CT
**38+π**	-4.38	-2.10	2.29	3.27	CT	2.51	LE

Photoluminescence (PL) Decay Lifetime Data

A. Lifetime Plots

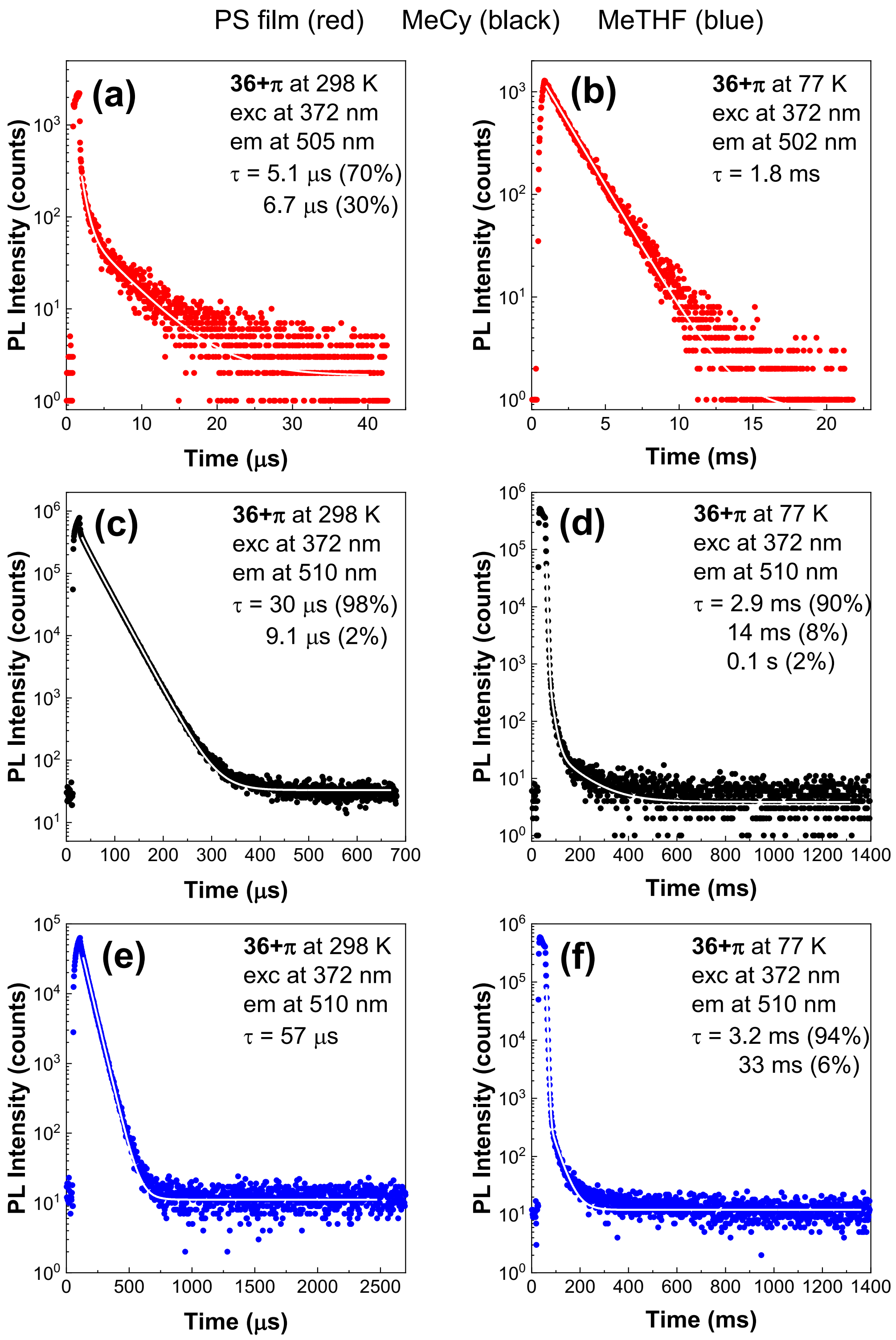

Supplementary Figure 5. Emission lifetime decay for **36+π** in (a-b) PS film, (c-d) MeCy, and (e-f) MeTHF at 298 K (left side) and 77 K (right side).

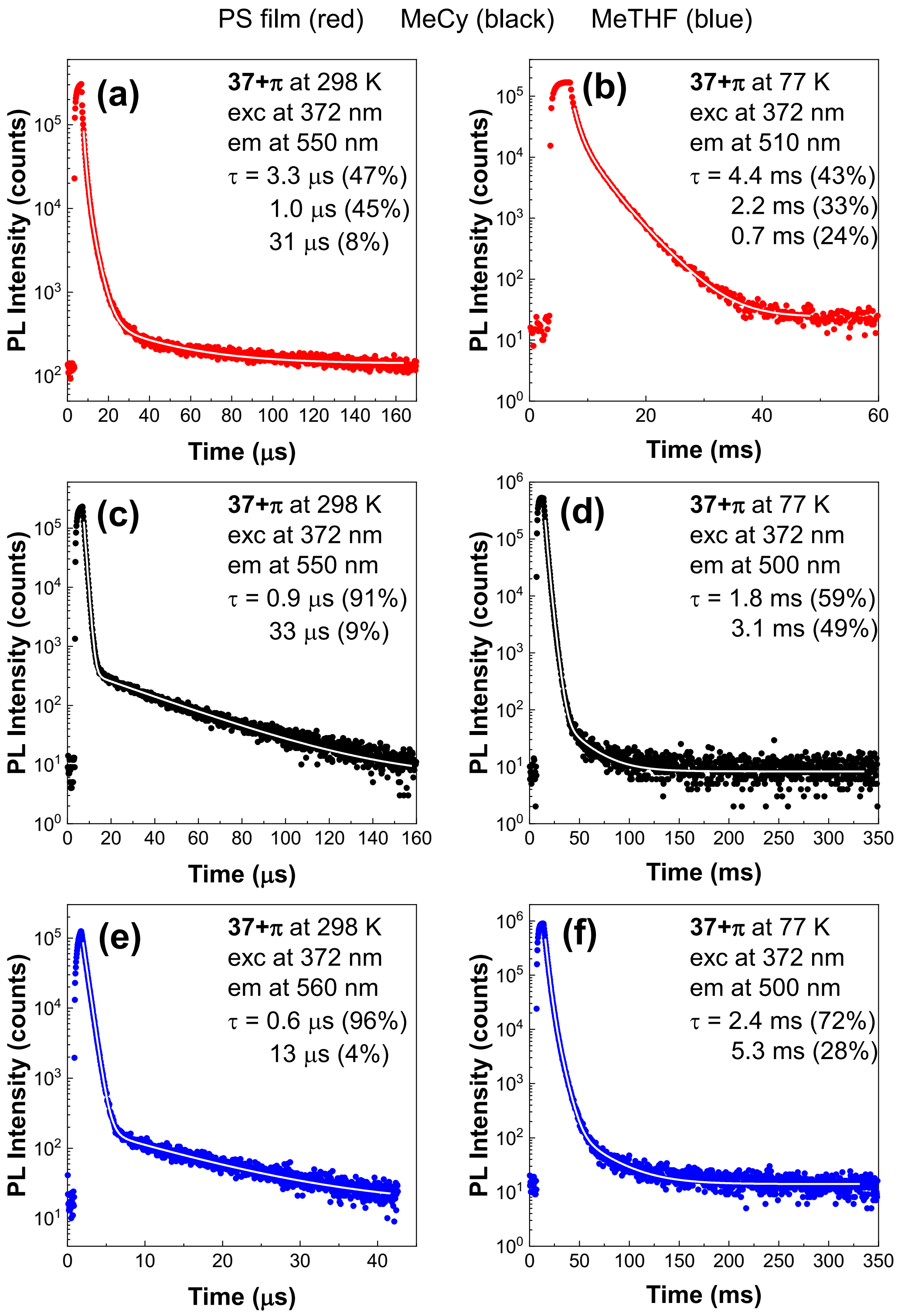

Supplementary Figure 6. Emission lifetime decay for **37+π** in (a-b) PS film, (c-d) MeCy, and (e-f) MeTHF at 298 K (left side) and 77 K (right side).

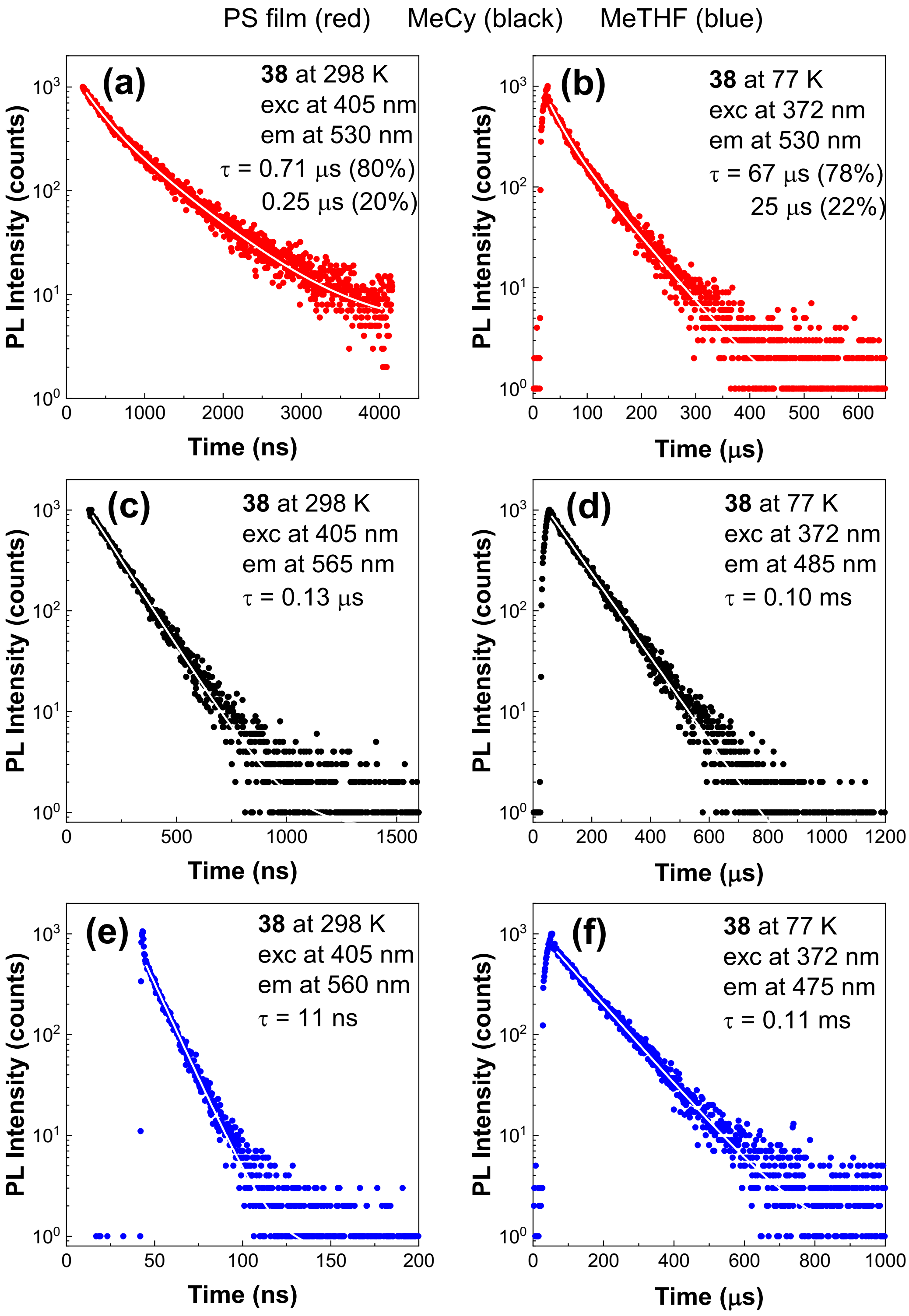

Supplementary Figure 7. Emission lifetime decay for **38** in (a-b) PS film, (c-d) MeCy, and (e-f) MeTHF at 298 K (left side) and 77 K (right side).

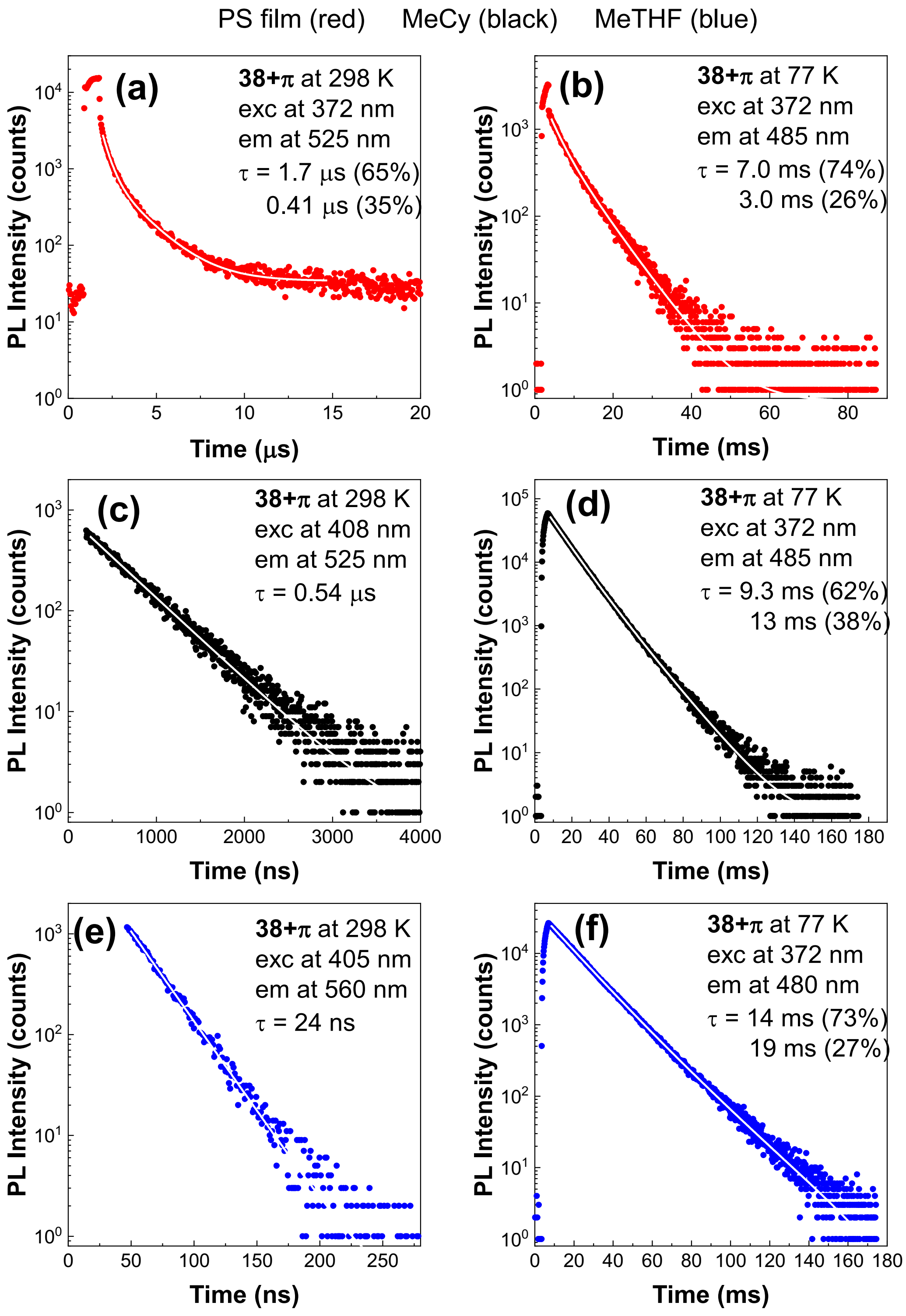

Supplementary Figure 8. Emission lifetime decay for **38+π** in (a-b) PS film, (c-d) MeCy, and (e-f) MeTHF at 298 K (left side) and 77 K (right side). In **37+π**, in both MeCy and MeTHF at 298 K

a biexponential decay is observed. The slow lifetime is in the range of tens of microseconds. It is assigned to p-type delayed fluorescence caused by a bimolecular triplet-triplet annihilation.

^{1}H NMR Spectra

Purified Product, 1H NMR

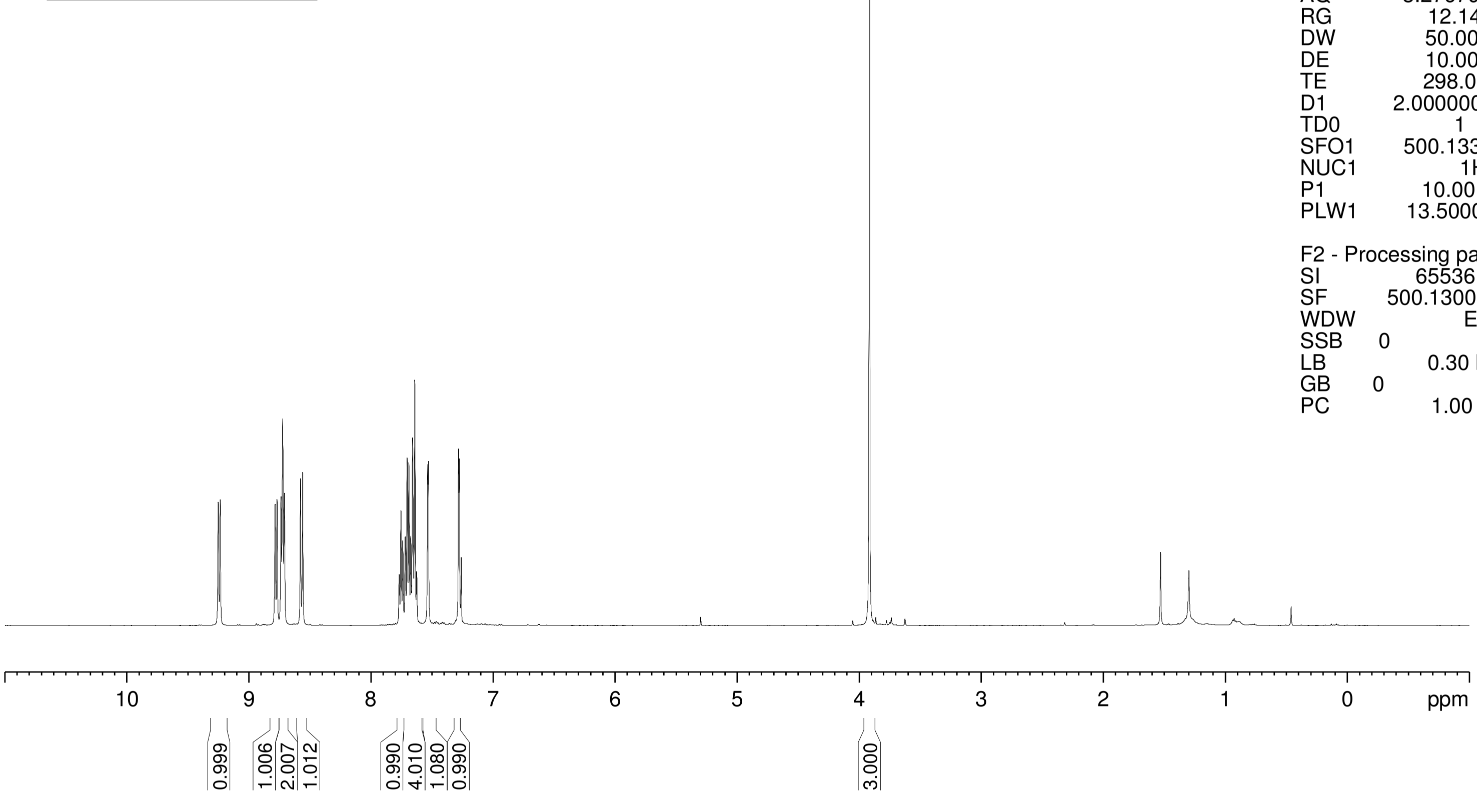

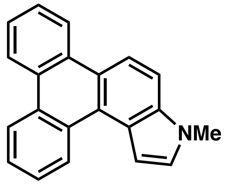

16

Current Data Parameters
NAME JVC-2018-087
EXPNO 3
PROCNO 1

F2 - Acquisition Parameters
Date_ 20180619
Time 17.09 h
INSTRUM av500
PROBHD Z119248_0002 (
PULPROG zg30
TD 65536
SOLVENT CDCl3
NS 24
DS 0
SWH 10000.000 Hz
FIDRES 0.305176 Hz
AQ 3.2767999 sec
RG 12.14
DW 50.000 usec
DE 10.00 usec
TE 298.0 K
D1 2.00000000 sec
TD0 1
SFO1 500.1330008 MHz
NUC1 1H
P1 10.00 usec
PLW1 13.50000000 W

F2 - Processing parameters
SI 65536
SF 500.1300120 MHz
WDW EM
SSB 0
LB 0.30 Hz
GB 0
PC 1.00

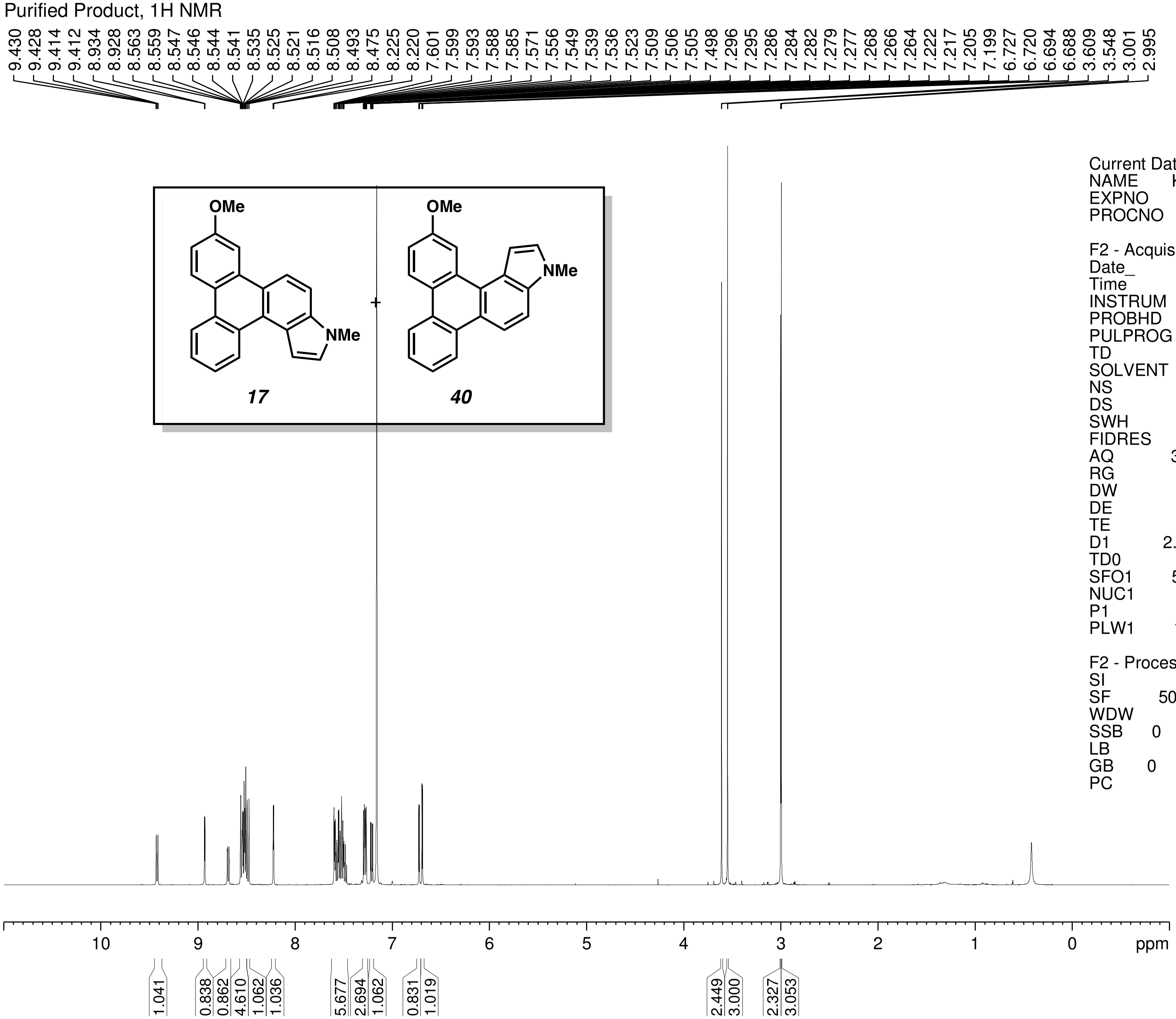

Current Data Parameters
NAME KAS-2018-052
EXPNO 1
PROCNO 1

F2 - Acquisition Parameters
Date_ 20180923
Time 17.34 h
INSTRUM av500
PROBHD Z119248_0002 (
PULPROG zg30
TD 65536
SOLVENT C6D6
NS 8
DS 0
SWH 10000.000 Hz
FIDRES 0.305176 Hz
AQ 3.2767999 sec
RG 12.14
DW 50.000 usec
DE 10.00 usec
TE 298.0 K
D1 2.00000000 sec
TD0 1
SFO1 500.1330008 MHz
NUC1 1H
P1 10.00 usec
PLW1 13.50000000 W

F2 - Processing parameters
SI 65536
SF 500.1299957 MHz
WDW EM
SSB 0
LB 0.30 Hz
GB 0
PC 1.00

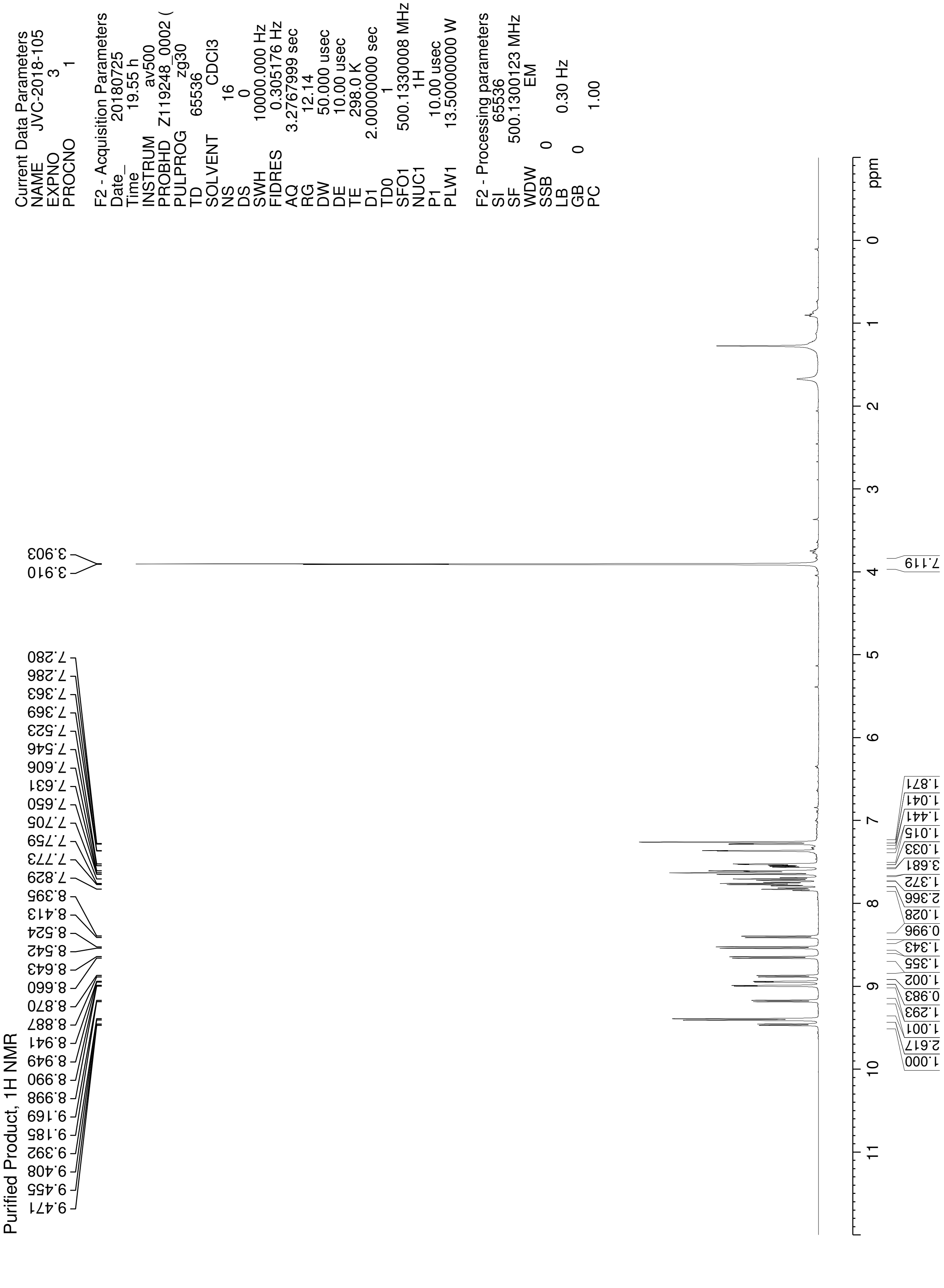

Purified Product, 1H NMR
9.471
9.455
9.408
9.392
9.185
9.169
8.998
8.990
8.949
8.941
8.887
8.870
8.660
8.643
8.542
8.524
8.413
8.395
7.829
7.773
7.759
7.705
7.650
7.631
7.606
7.546
7.523
7.369
7.363
7.286
7.280
3.910
3.903
Current Data Parameters
NAME JVC-2018-105
EXPNO 3
PROCNO 1
F2 - Acquisition Parameters
Date_ 20180725
Time 19.55 h
INSTRUM av500
PROBHD Z119248_0002 (
PULPROG zg30
TD 65536
SOLVENT CDCl3
NS 16
DS 0
SWH 10000.000 Hz
FIDRES 0.305176 Hz
AQ 3.2767999 sec
RG 12.14
DW 50.000 usec
DE 10.00 usec
TE 298.0 K
D1 2.00000000 sec
TD0 1
SFO1 500.1330008 MHz
NUC1 1H
P1 10.00 usec
PLW1 13.50000000 W
F2 - Processing parameters
SI 65536
SF 500.1300123 MHz
WDW EM
SSB 0
LB 0.30 Hz
GB 0
PC 1.00
1.000
2.617
1.001
1.293
0.983
1.002
1.355
1.343
0.996
1.028
2.366
1.372
3.681
1.033
1.015
1.441
1.041
1.871
7.119
ppm

Purified Product, 1H NMR

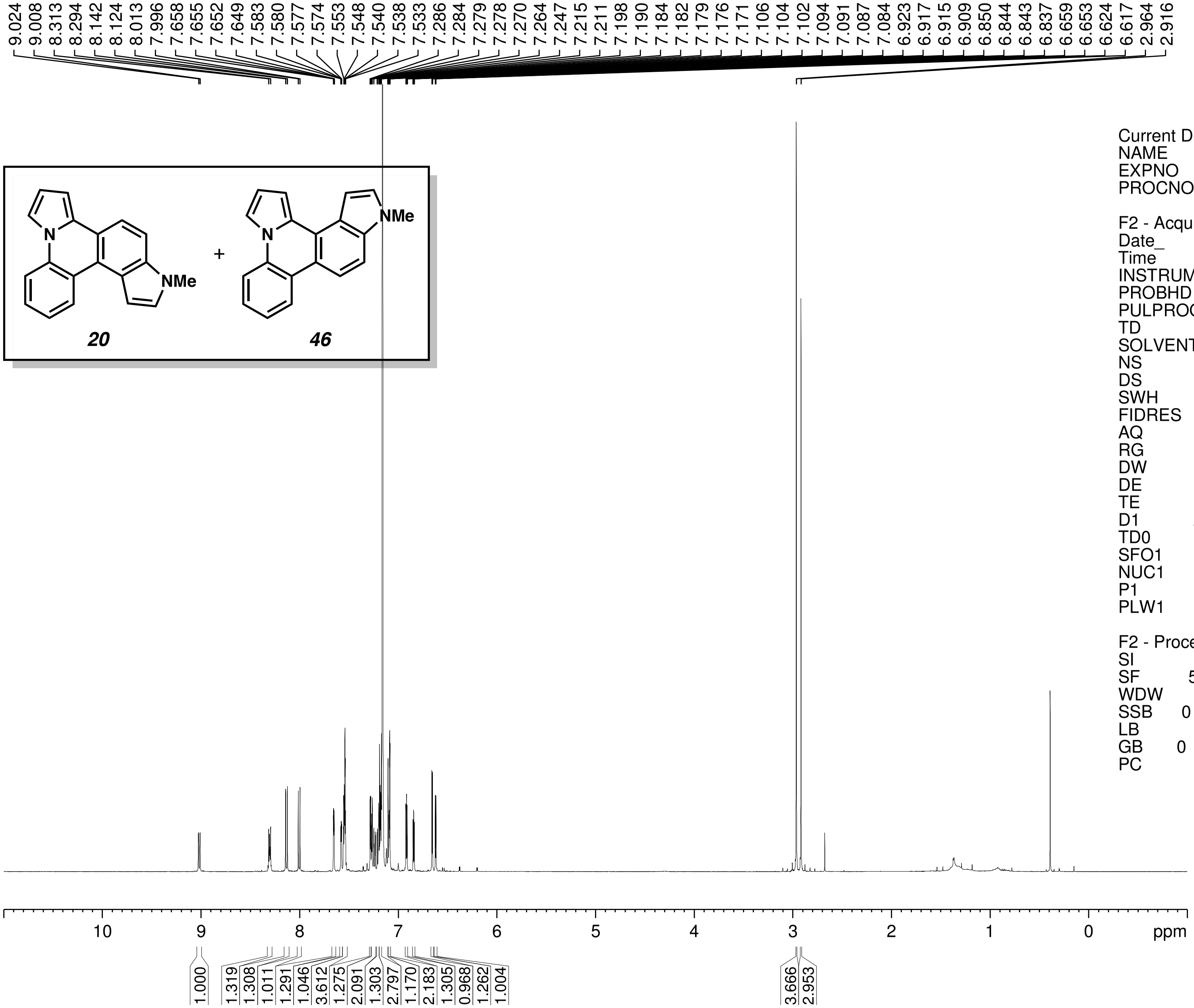

Current Data Parameters
NAME KAS-2019-015
EXPNO 1
PROCNO 1

F2 - Acquisition Parameters
Date_ 20190122
Time 20.00 h
INSTRUM av500
PROBHD Z119248_0002 (
PULPROG zg30
TD 65536
SOLVENT C6D6
NS 8
DS 0
SWH 10000.000 Hz
FIDRES 0.305176 Hz
AQ 3.2767999 sec
RG 12.14
DW 50.000 usec
DE 10.00 usec
TE 298.0 K
D1 2.00000000 sec
TD0 1
SFO1 500.1330008 MHz
NUC1 1H
P1 10.00 usec
PLW1 13.50000000 W

F2 - Processing parameters
SI 65536
SF 500.1299961 MHz
WDW EM
SSB 0
LB 0.30 Hz
GB 0
PC 1.00

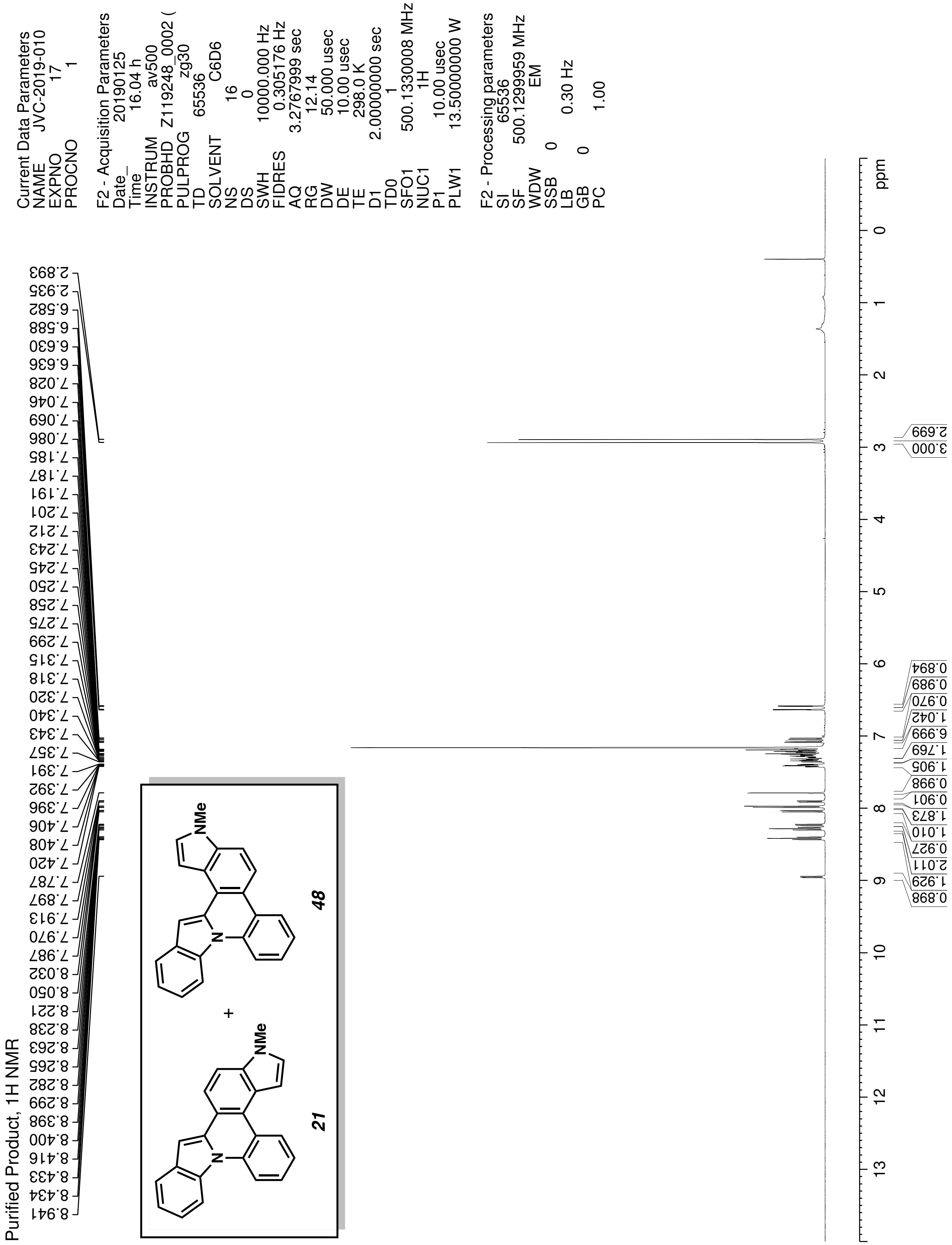

Purified Product, 1H NMR
8.941 8.434 8.433 8.416 8.400 8.398 8.299 8.282 8.265 8.263 8.238 8.221 8.050 8.032 7.987 7.970 7.913 7.897 7.787 7.420 7.408 7.406 7.396 7.392 7.391 7.357 7.343 7.340 7.320 7.318 7.315 7.299 7.275 7.258 7.250 7.245 7.243 7.212 7.201 7.191 7.187 7.185 7.086 7.069 7.046 7.028 6.636 6.630 6.588 6.582 2.935 2.893
N
NMe
21
+
48
Current Data Parameters
NAME JVC-2019-010
EXPNO 17
PROCNO 1
F2 - Acquisition Parameters
Date_ 20190125
Time 16.04 h
INSTRUM av500
PROBHD Z119248_0002 (
PULPROG zg30
TD 65536
SOLVENT C6D6
NS 16
DS 0
SWH 10000.000 Hz
FIDRES 0.305176 Hz
AQ 3.2767999 sec
RG 12.14
DW 50.000 usec
DE 10.00 usec
TE 298.0 K
D1 2.00000000 sec
TD0 1
SFO1 500.1330008 MHz
NUC1 1H
P1 10.00 usec
PLW1 13.50000000 W
F2 - Processing parameters
SI 65536
SF 500.1299959 MHz
WDW EM
SSB 0
LB 0.30 Hz
GB 0
PC 1.00
0.898 1.929 2.011 0.927 1.010 1.873 0.901 0.998 1.905 1.769 6.999 1.042 0.970 0.989 0.894 3.000 2.699
13 12 11 10 9 8 7 6 5 4 3 2 1 0 ppm

Purified Product, 1H NMR

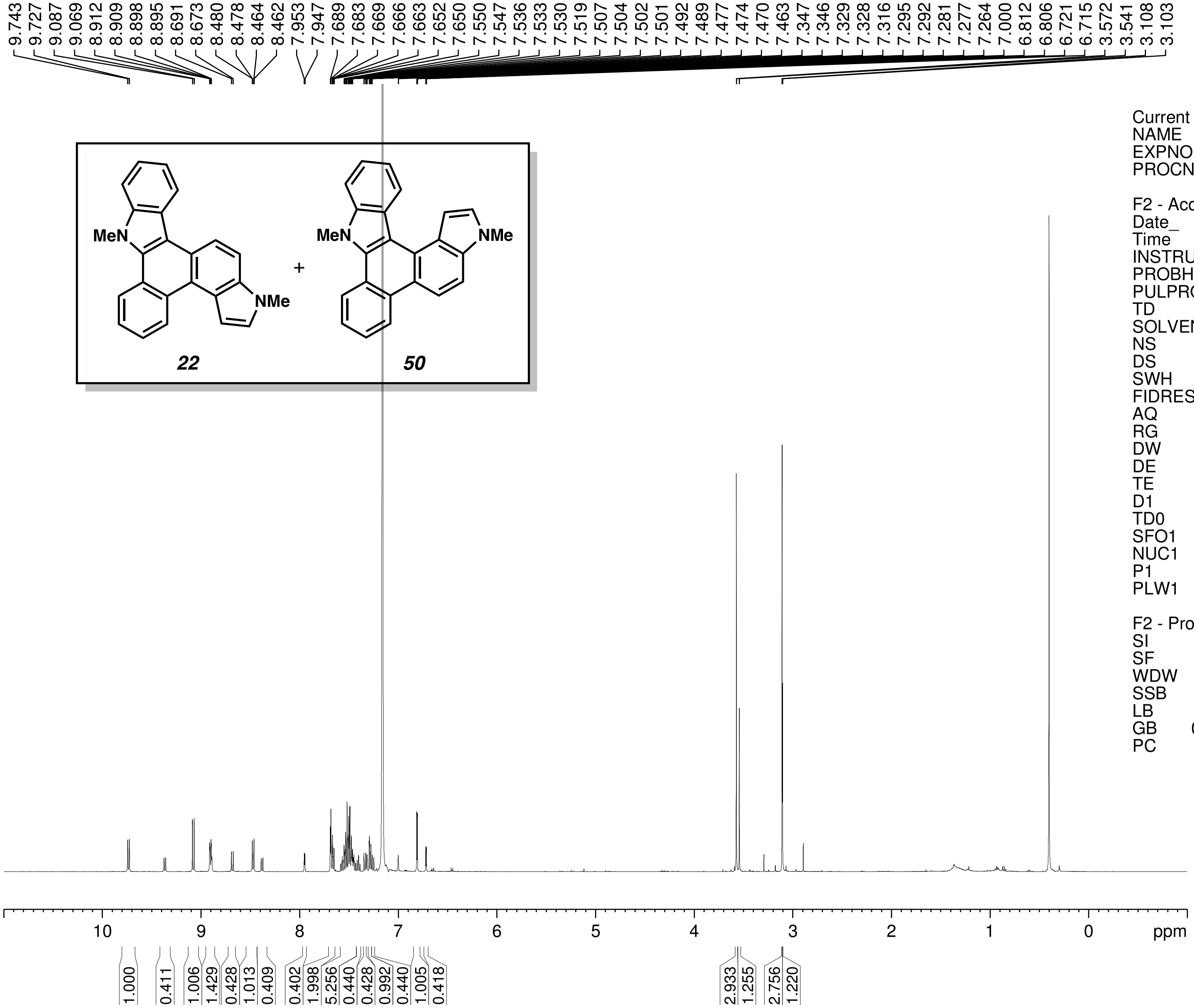

Current Data Parameters
NAME JVC-2018-107-3-2D
EXPNO 1
PROCNO 1

F2 - Acquisition Parameters
Date_ 20180819
Time 20.13 h
INSTRUM av500
PROBHD Z119248_0002 (
PULPROG zg30
TD 65536
SOLVENT C6D6
NS 16
DS 0
SWH 10000.000 Hz
FIDRES 0.305176 Hz
AQ 3.2767999 sec
RG 12.14
DW 50.000 usec
DE 10.00 usec
TE 298.0 K
D1 2.00000000 sec
TD0 1
SFO1 500.1330008 MHz
NUC1 1H
P1 10.00 usec
PLW1 13.50000000 W

F2 - Processing parameters
SI 65536
SF 500.1299959 MHz
WDW EM
SSB 0
LB 0.30 Hz
GB 0
PC 1.00

Purified Product, 1H NMR

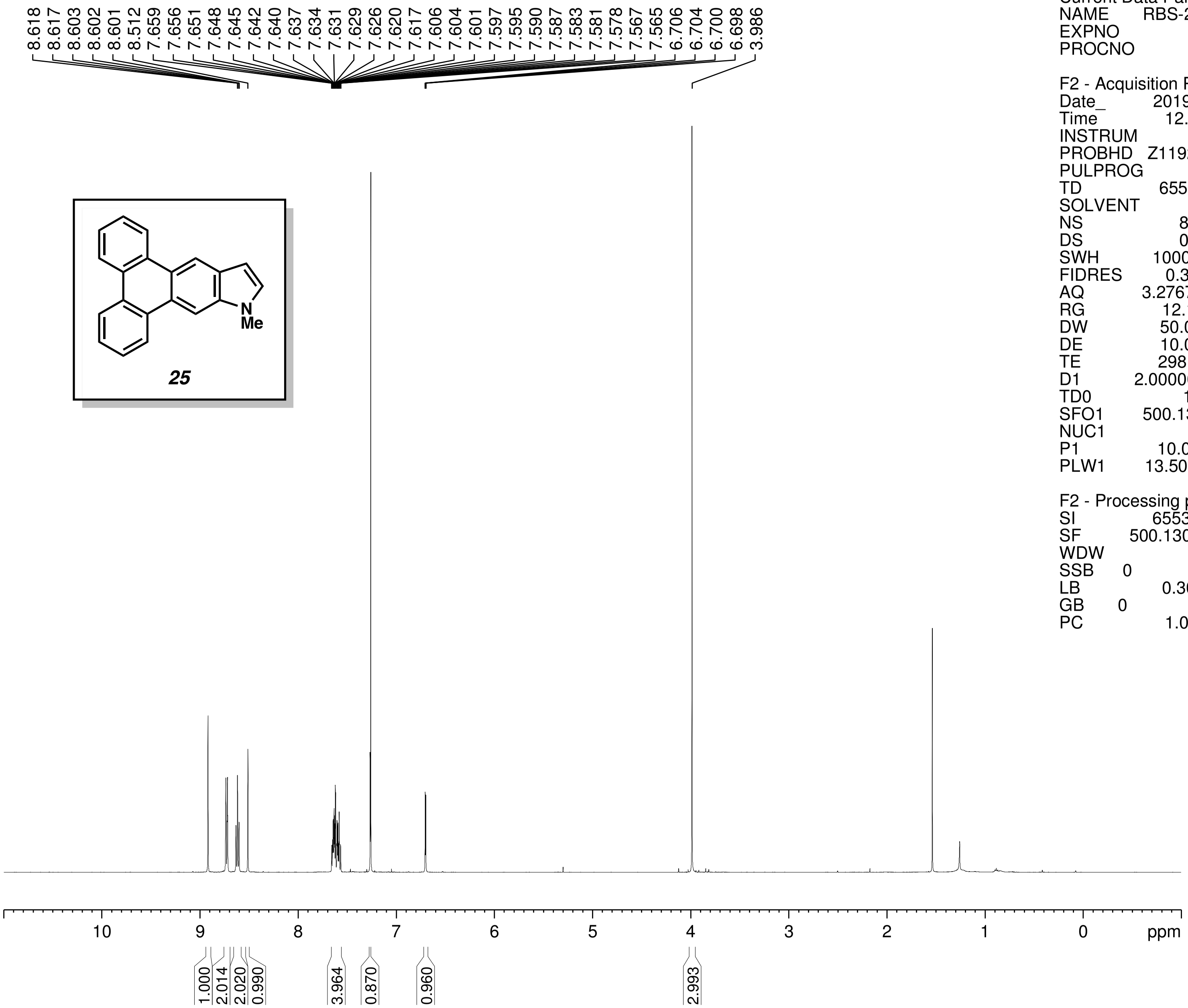

25

Current Data Parameters
NAME RBS-2019-012
EXPNO 3
PROCNO 1

F2 - Acquisition Parameters
Date_ 20190127
Time 12.41 h
INSTRUM av500
PROBHD Z119248_0002 (
PULPROG zg30
TD 65536
SOLVENT CDCl3
NS 8
DS 0
SWH 10000.000 Hz
FIDRES 0.305176 Hz
AQ 3.2767999 sec
RG 12.14
DW 50.000 usec
DE 10.00 usec
TE 298.0 K
D1 2.00000000 sec
TD0 1
SFO1 500.1330008 MHz
NUC1 1H
P1 10.00 usec
PLW1 13.50000000 W

F2 - Processing parameters
SI 65536
SF 500.1300122 MHz
WDW EM
SSB 0
LB 0.30 Hz
GB 0
PC 1.00

Purified Product, 1H NMR

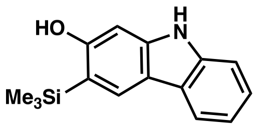

28

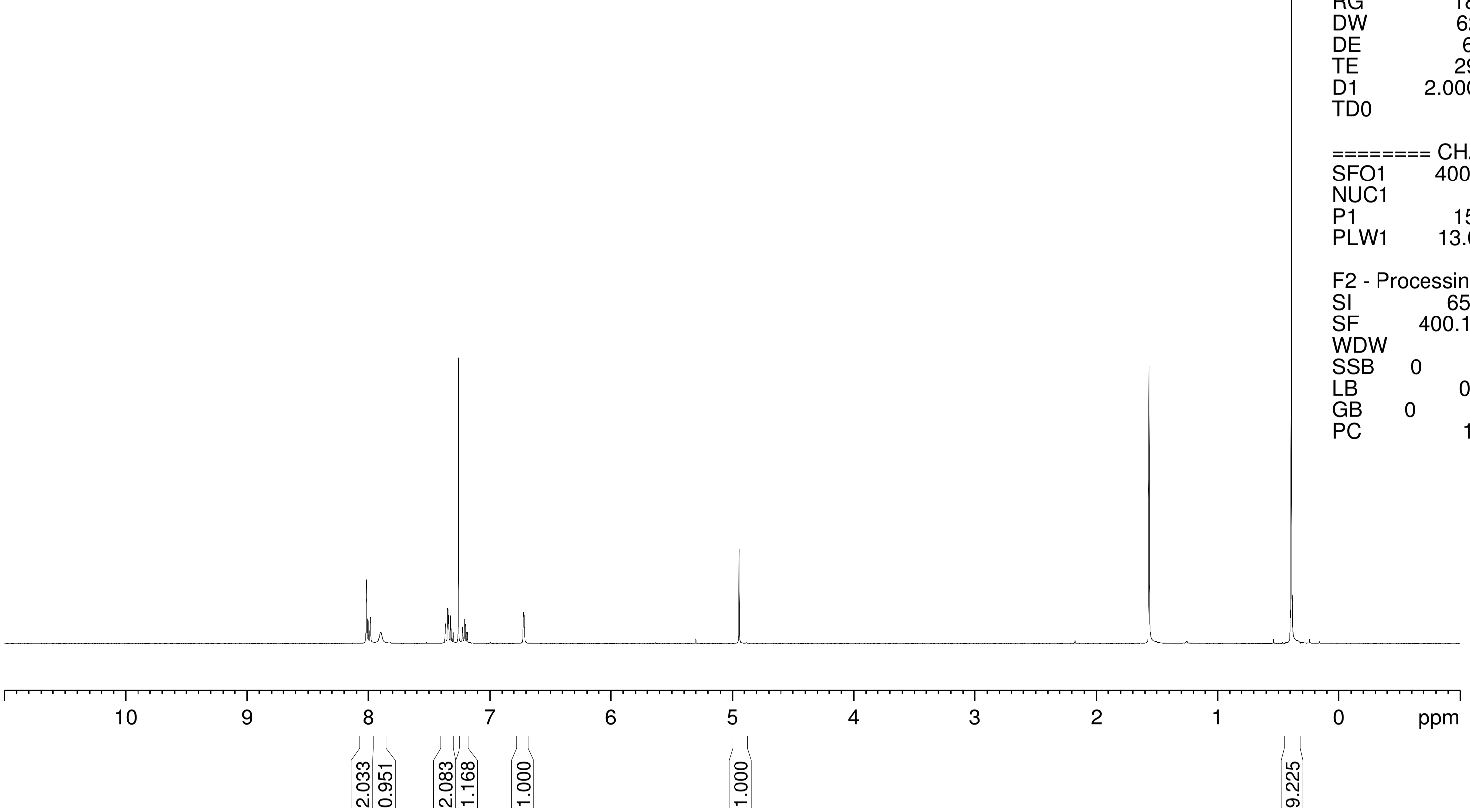

Current Data Parameters
NAME RBS-2019-139
EXPNO 3
PROCNO 1

F2 - Acquisition Parameters
Date_ 20190918
Time 19.44
INSTRUM av400
PROBHD 5 mm PABBO BB/
PULPROG zg30
TD 52882
SOLVENT CDCl3
NS 24
DS 0
SWH 8012.820 Hz
FIDRES 0.151523 Hz
AQ 3.2998369 sec
RG 189.85
DW 62.400 usec
DE 6.50 usec
TE 296.3 K
D1 2.00000000 sec
TD0 1

======== CHANNEL f1 ======
SFO1 400.1324008 MHz
NUC1 1H
P1 15.00 usec
PLW1 13.00000000 W

F2 - Processing parameters
SI 65536
SF 400.1300184 MHz
WDW EM
SSB 0
LB 0.30 Hz
GB 0
PC 1.00

Purified Product, 1H NMR

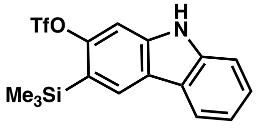

29

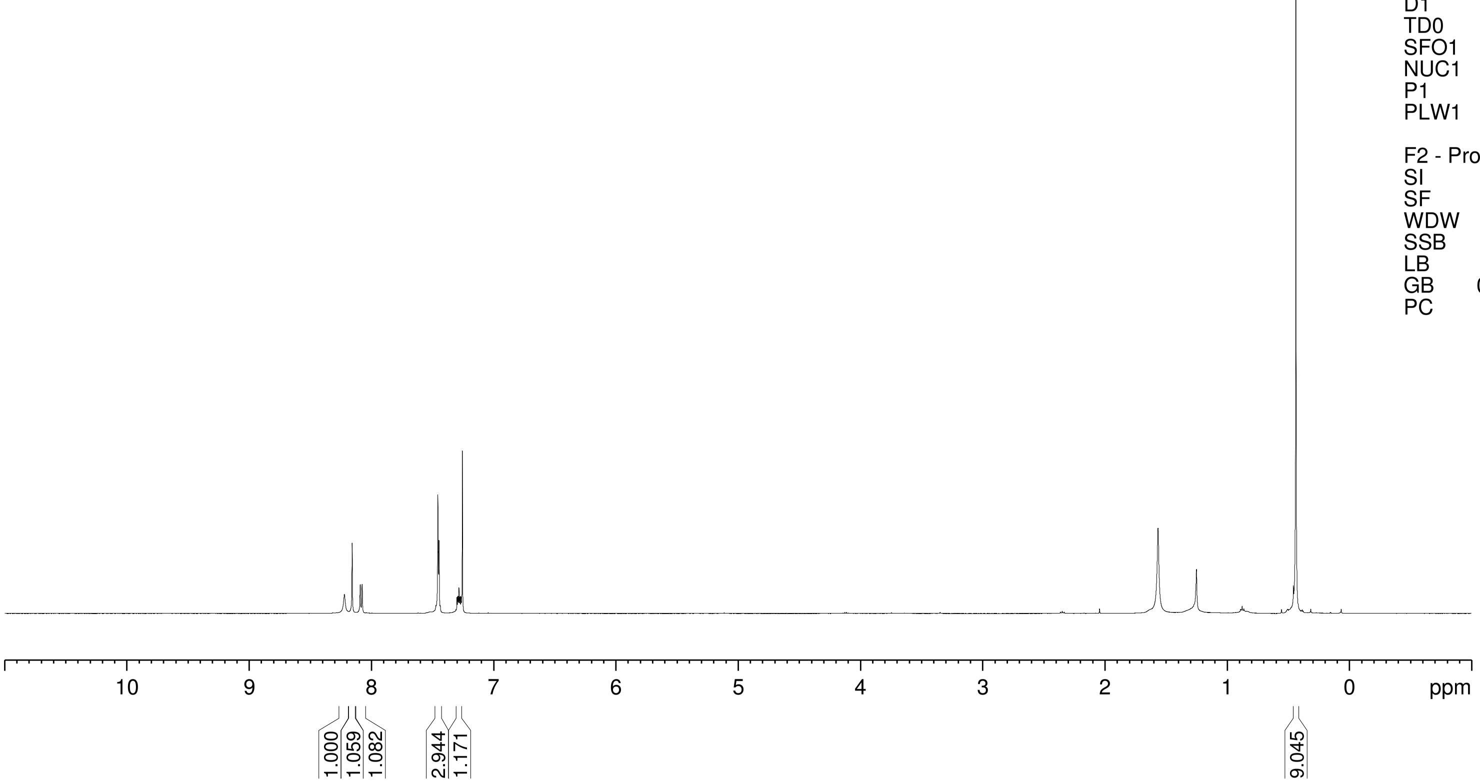

Current Data Parameters
NAME KAS-2021-000
EXPNO 200
PROCNO 1

F2 - Acquisition Parameters
Date_ 20210728
Time 15.09 h
INSTRUM av500
PROBHD Z119248_0002 (
PULPROG zg30
TD 65536
SOLVENT CDCl3
NS 16
DS 0
SWH 10000.000 Hz
FIDRES 0.305176 Hz
AQ 3.2767999 sec
RG 12.14
DW 50.000 usec
DE 10.00 usec
TE 298.0 K
D1 2.00000000 sec
TD0 1
SFO1 500.1330008 MHz
NUC1 1H
P1 10.00 usec
PLW1 13.50000000 W

F2 - Processing parameters
SI 65536
SF 500.1300146 MHz
WDW EM
SSB 0
LB 0.30 Hz
GB 0
PC 1.00

Purified Material, 1H NMR

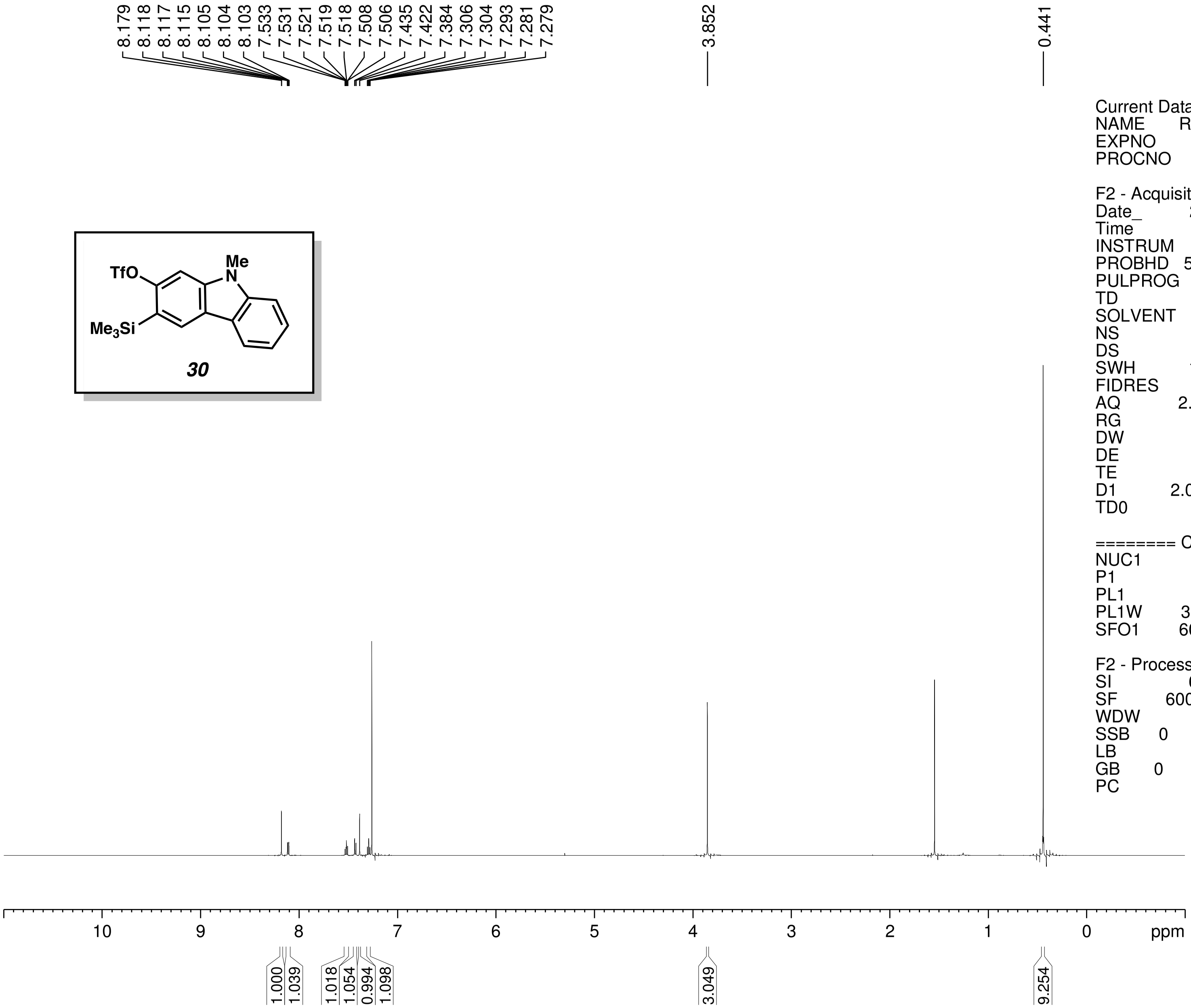

Current Data Parameters
NAME RBS-2019-153
EXPNO 1
PROCNO 1

F2 - Acquisition Parameters
Date_ 20190929
Time 20.34
INSTRUM av600
PROBHD 5 mm TBI5
PULPROG zg30
TD 65536
SOLVENT CDCl3
NS 16
DS 0
SWH 12376.237 Hz
FIDRES 0.188846 Hz
AQ 2.6476543 sec
RG 181
DW 40.400 usec
DE 6.50 usec
TE 294.5 K
D1 2.00000000 sec
TD0 1

======== CHANNEL f1 ========
NUC1 1H
P1 11.55 usec
PL1 -2.00 dB
PL1W 39.81071854 W
SFO1 600.1336008 MHz

F2 - Processing parameters
SI 65536
SF 600.1300282 MHz
WDW EM
SSB 0
LB 0.30 Hz
GB 0
PC 1.00

Purified Material, 1H NMR

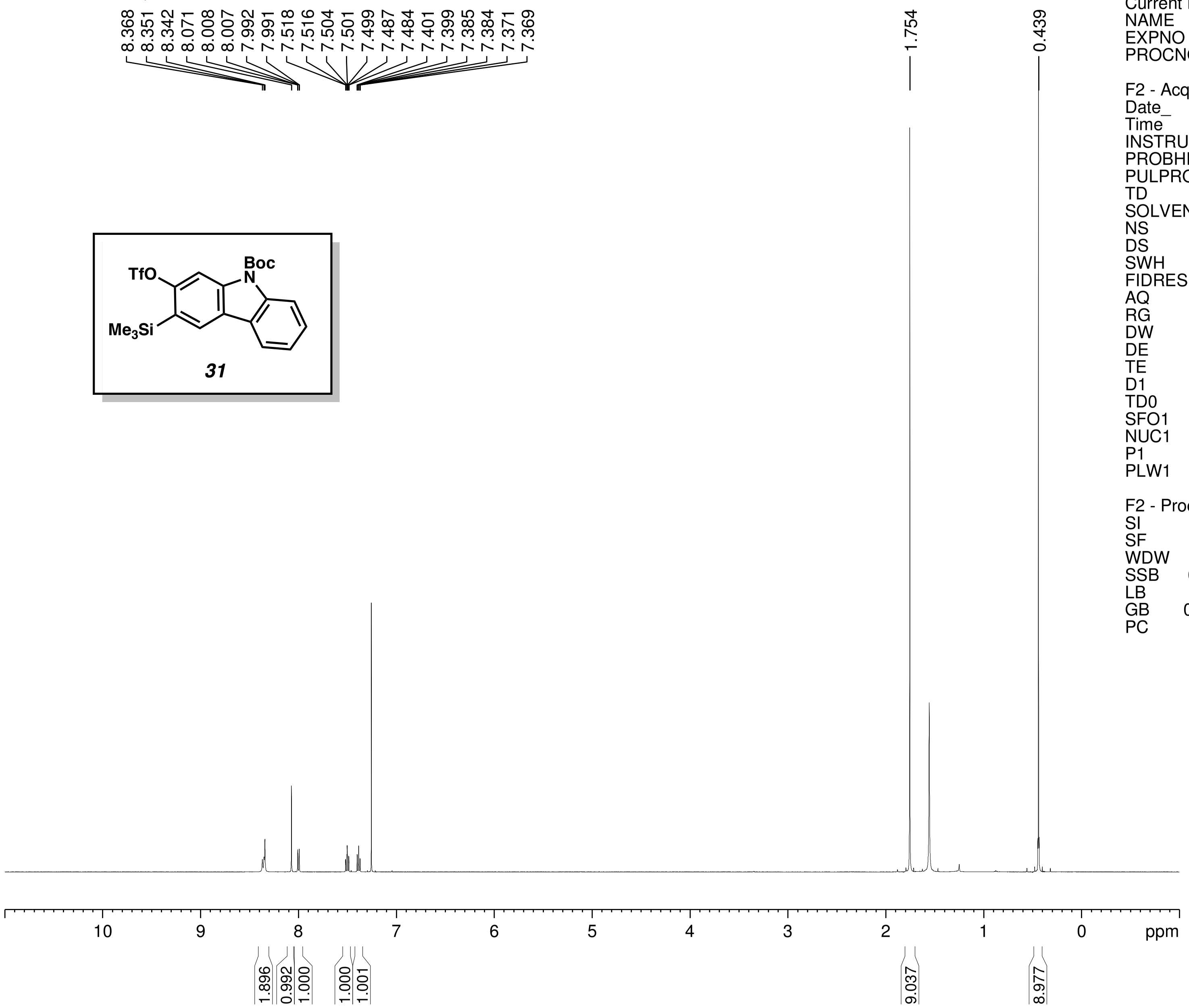

Current Data Parameters
NAME KAS-2021-000
EXPNO 110
PROCNO 1

F2 - Acquisition Parameters
Date_ 20210722
Time 16.19 h
INSTRUM av500
PROBHD Z119248_0002 (
PULPROG zg30
TD 65536
SOLVENT CDCl3
NS 8
DS 0
SWH 10000.000 Hz
FIDRES 0.305176 Hz
AQ 3.2767999 sec
RG 12.14
DW 50.000 usec
DE 10.00 usec
TE 298.0 K
D1 2.00000000 sec
TD0 1
SFO1 500.1330008 MHz
NUC1 1H
P1 10.00 usec
PLW1 13.50000000 W

F2 - Processing parameters
SI 65536
SF 500.1300146 MHz
WDW EM
SSB 0
LB 0.30 Hz
GB 0
PC 1.00

Purified Product, 1H NMR

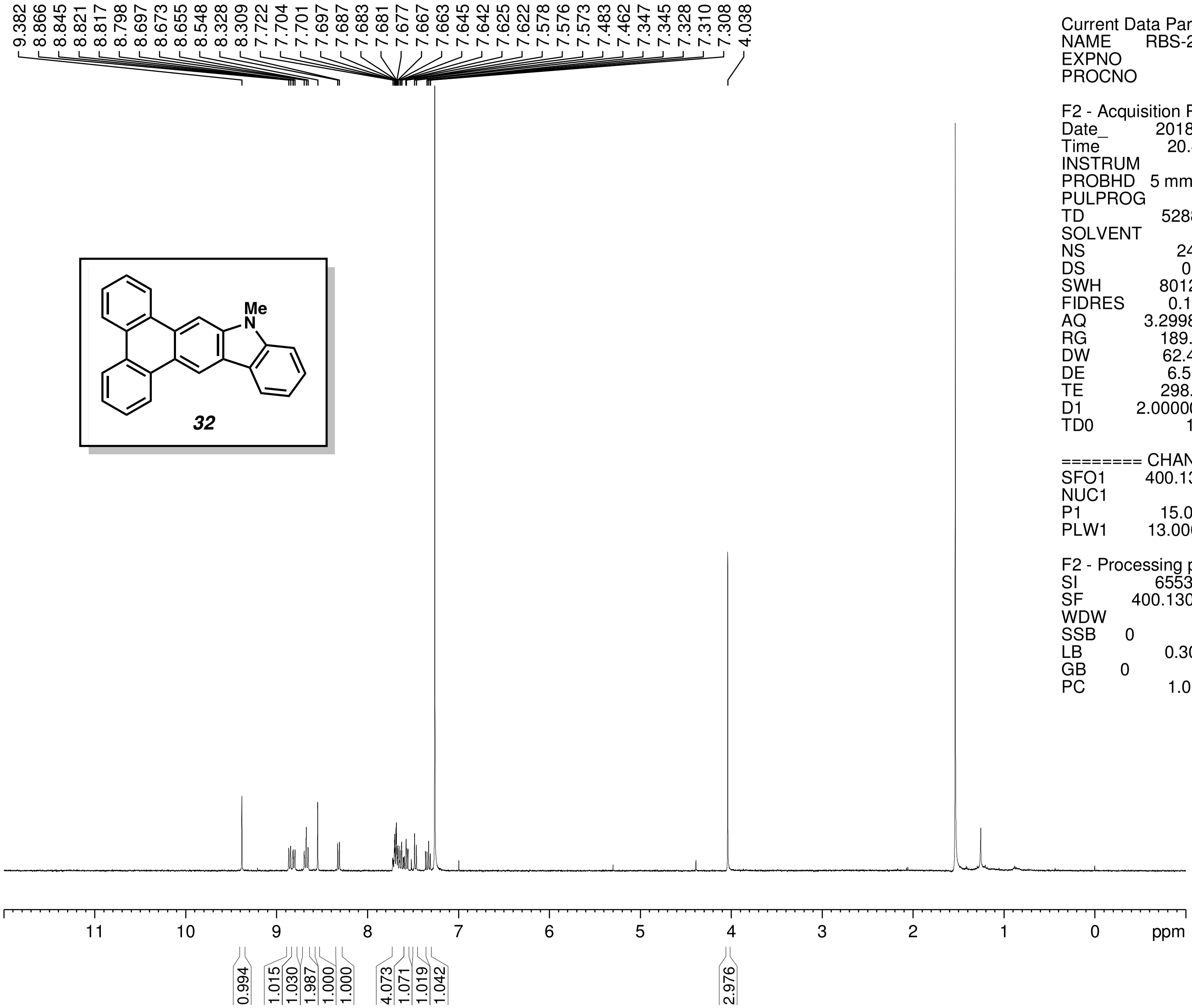

Current Data Parameters
NAME RBS-2018-171
EXPNO 3
PROCNO 1

F2 - Acquisition Parameters
Date_ 20181023
Time 20.40
INSTRUM av400
PROBHD 5 mm PABBO BB/
PULPROG zg30
TD 52882
SOLVENT CDCl3
NS 24
DS 0
SWH 8012.820 Hz
FIDRES 0.151523 Hz
AQ 3.2998369 sec
RG 189.85
DW 62.400 usec
DE 6.50 usec
TE 298.3 K
D1 2.00000000 sec
TD0 1

======== CHANNEL f1 =====
SFO1 400.1324008 MHz
NUC1 1H
P1 15.00 usec
PLW1 13.00000000 W

F2 - Processing parameters
SI 65536
SF 400.1300177 MHz
WDW EM
SSB 0
LB 0.30 Hz
GB 0
PC 1.00

Purified Product, 1H NMR

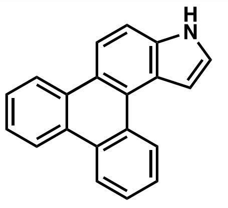

34

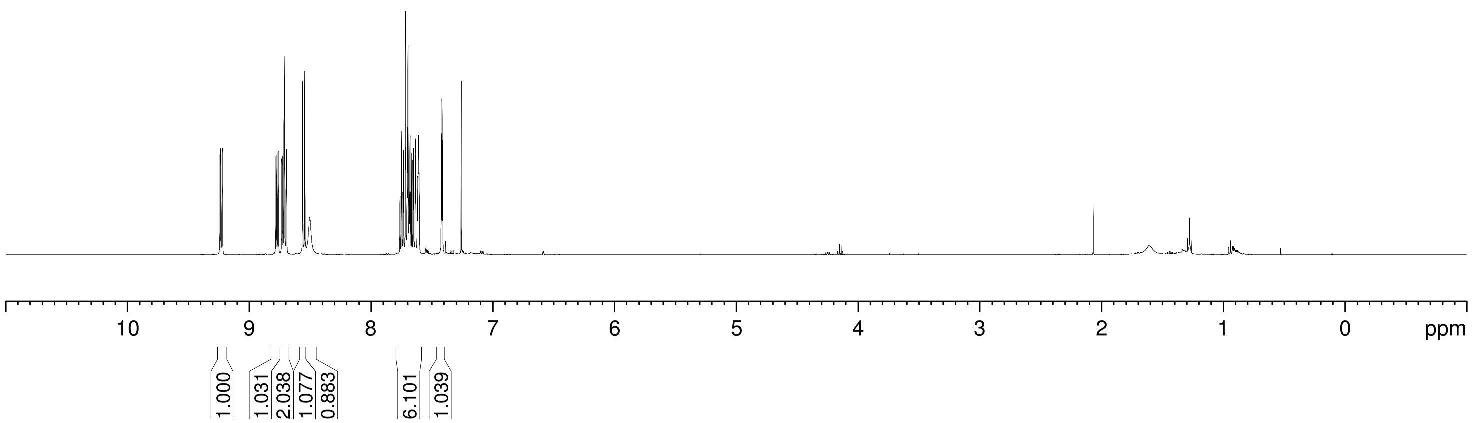

Current Data Parameters
NAME KAS-2021-000
EXPNO 32
PROCNO 1

F2 - Acquisition Parameters
Date_ 20210714
Time 13.56 h
INSTRUM av500
PROBHD Z119248_0002 (
PULPROG zg30
TD 65536
SOLVENT CDCl3
NS 8
DS 0
SWH 10000.000 Hz
FIDRES 0.305176 Hz
AQ 3.2767999 sec
RG 26.59
DW 50.000 usec
DE 10.00 usec
TE 298.0 K
D1 2.00000000 sec
TD0 1
SFO1 500.1330008 MHz
NUC1 1H
P1 10.00 usec
PLW1 13.50000000 W

F2 - Processing parameters
SI 65536
SF 500.1300127 MHz
WDW EM
SSB 0
LB 0.30 Hz
GB 0
PC 1.00

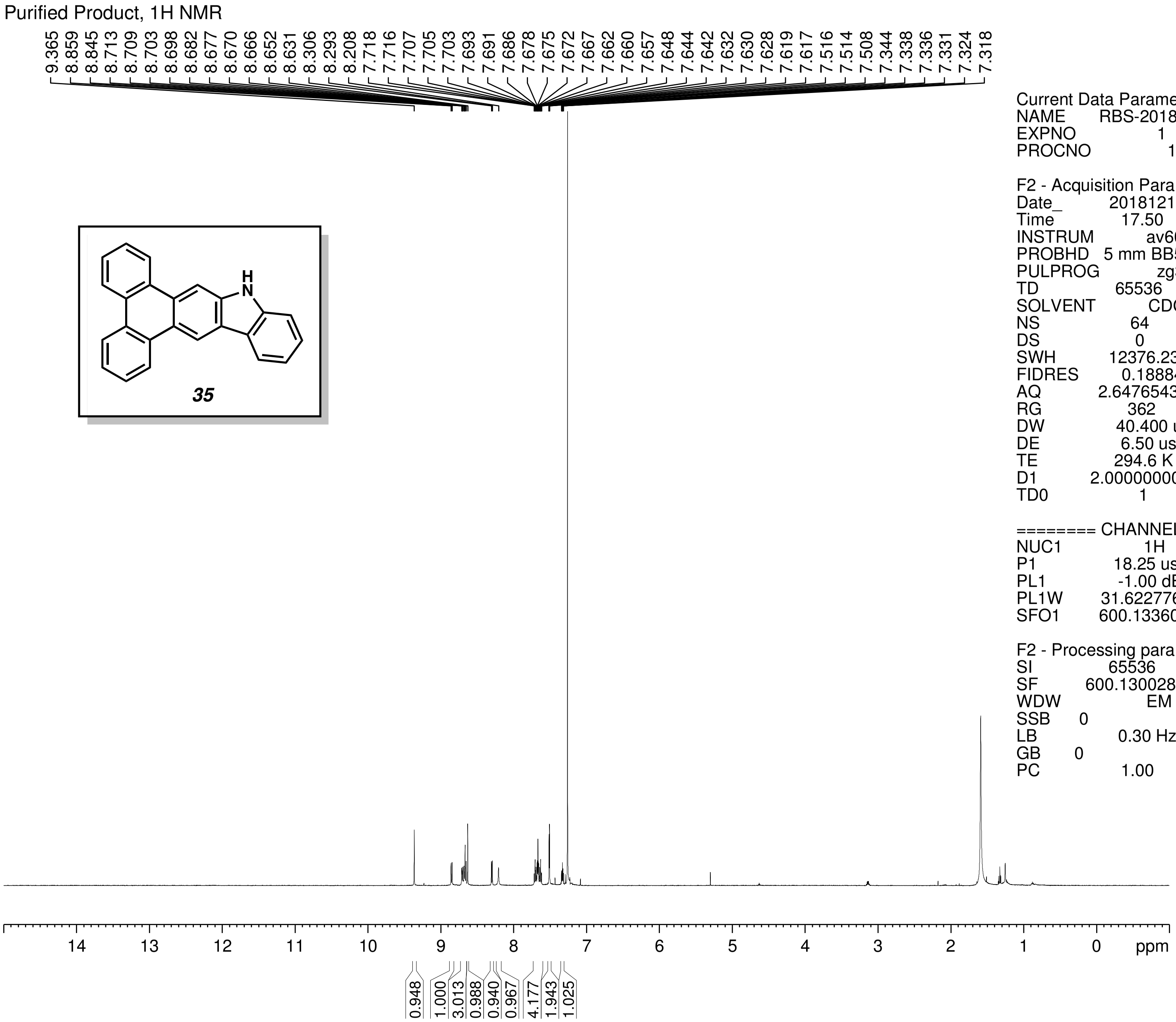

Current Data Parameters
NAME RBS-2018-202
EXPNO 1
PROCNO 1

F2 - Acquisition Parameters
Date_ 20181217
Time 17.50
INSTRUM av600
PROBHD 5 mm BB5
PULPROG zg30
TD 65536
SOLVENT CDCl3
NS 64
DS 0
SWH 12376.237 Hz
FIDRES 0.188846 Hz
AQ 2.6476543 sec
RG 362
DW 40.400 usec
DE 6.50 usec
TE 294.6 K
D1 2.00000000 sec
TD0 1

======== CHANNEL f1 ========
NUC1 1H
P1 18.25 usec
PL1 -1.00 dB
PL1W 31.62277603 W
SFO1 600.1336008 MHz

F2 - Processing parameters
SI 65536
SF 600.1300286 MHz
WDW EM
SSB 0
LB 0.30 Hz
GB 0
PC 1.00

Purified Product, 1H NMR

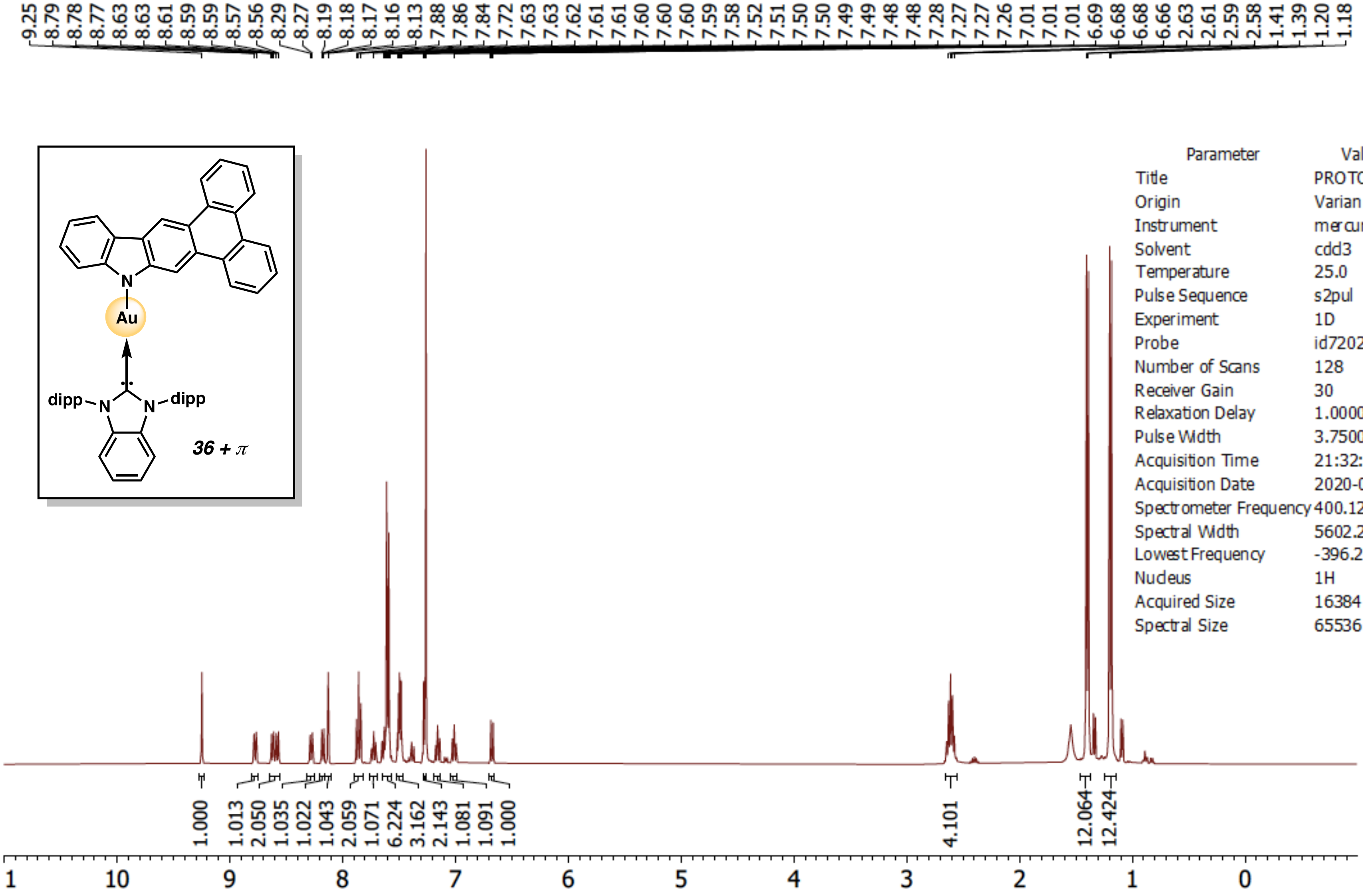

Parameter	Value
Title	PROTON_01
Origin	Varian
Instrument	mercury
Solvent	cdd3
Temperature	25.0
Pulse Sequence	s2pul
Experiment	1D
Probe	id7202
Number of Scans	128
Receiver Gain	30
Relaxation Delay	1.0000
Pulse Width	3.7500
Acquisition Time	21:32:55
Acquisition Date	2020-02-28
Spectrometer Frequency	400.12
Spectral Width	5602.2
Lowest Frequency	-396.2
Nucleus	1H
Acquired Size	16384
Spectral Size	65536

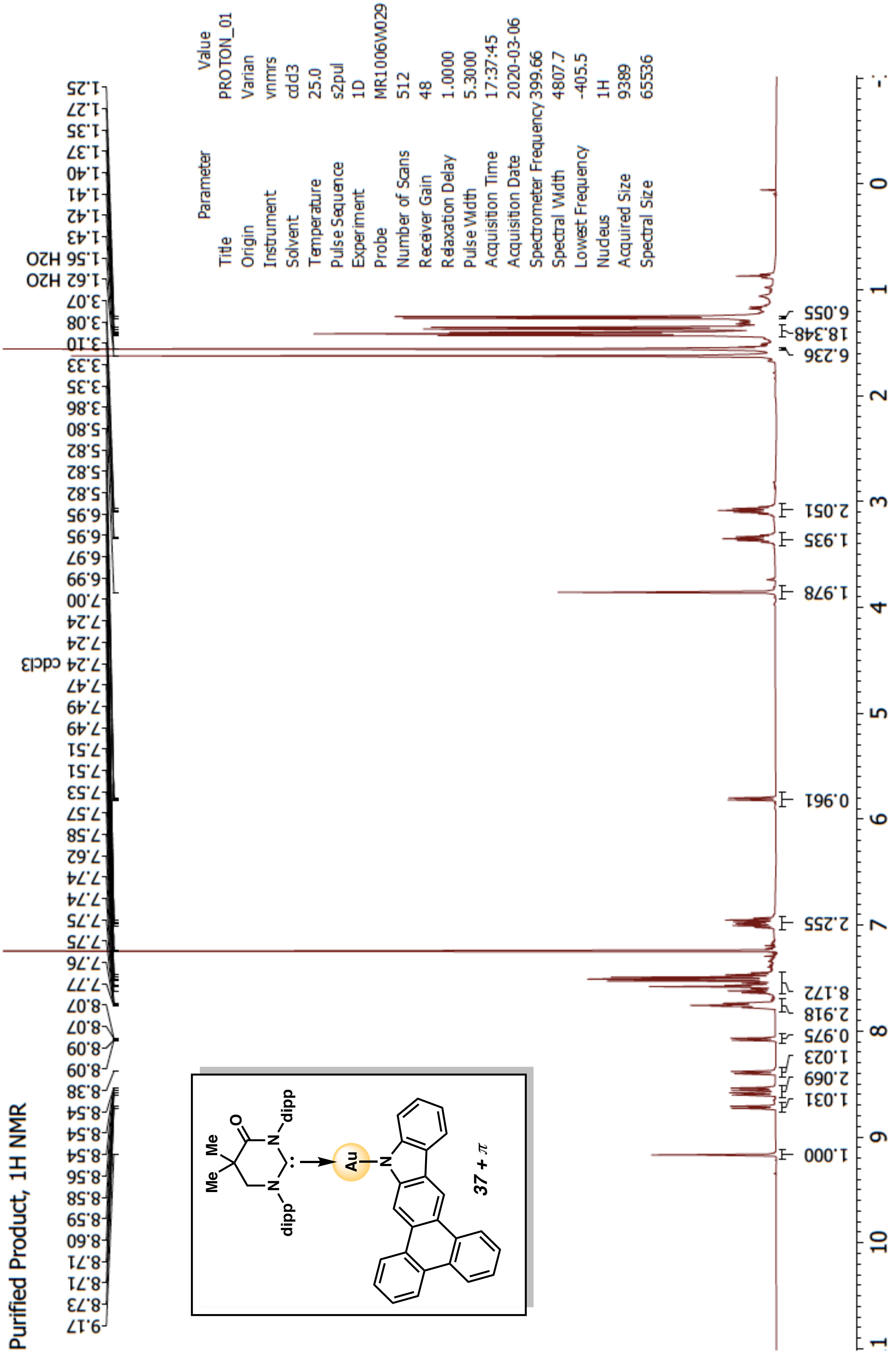
Purified Product, 1H NMR
9.17 8.73 8.71 8.71 8.60 8.59 8.58 8.56 8.54 8.54 8.54 8.38 8.09 8.09 8.07 8.07 7.77 7.76 7.75 7.75 7.74 7.74 7.62 7.58 7.57 7.53 7.51 7.51 7.49 7.49 7.47 7.24 cdcl3 7.24 7.24 7.00 6.99 6.97 6.95 6.95 5.82 5.82 5.82 5.80 3.86 3.35 3.33 3.10 3.08 3.07 1.62 H2O 1.56 H2O 1.43 1.42 1.41 1.40 1.37 1.35 1.27 1.25
Parameter / Value
Title / PROTON_01
Origin / Varian
Instrument / vnmrs
Solvent / cdcl3
Temperature / 25.0
Pulse Sequence / s2pul
Experiment / 1D
Probe / MR1006W029
Number of Scans / 512
Receiver Gain / 48
Relaxation Delay / 1.0000
Pulse Width / 5.3000
Acquisition Time / 17:37:45
Acquisition Date / 2020-03-06
Spectrometer Frequency / 399.66
Spectral Width / 4807.7
Lowest Frequency / -405.5
Nucleus / 1H
Acquired Size / 9389
Spectral Size / 65536
1.000 1.031 2.069 1.023 0.975 2.918 8.172 2.255 0.961 1.978 1.935 2.051 6.236 18.348 6.055
11 10 9 8 7 6 5 4 3 2 1 0 -1
Me Me O N dipp dipp N Au N
37 + π

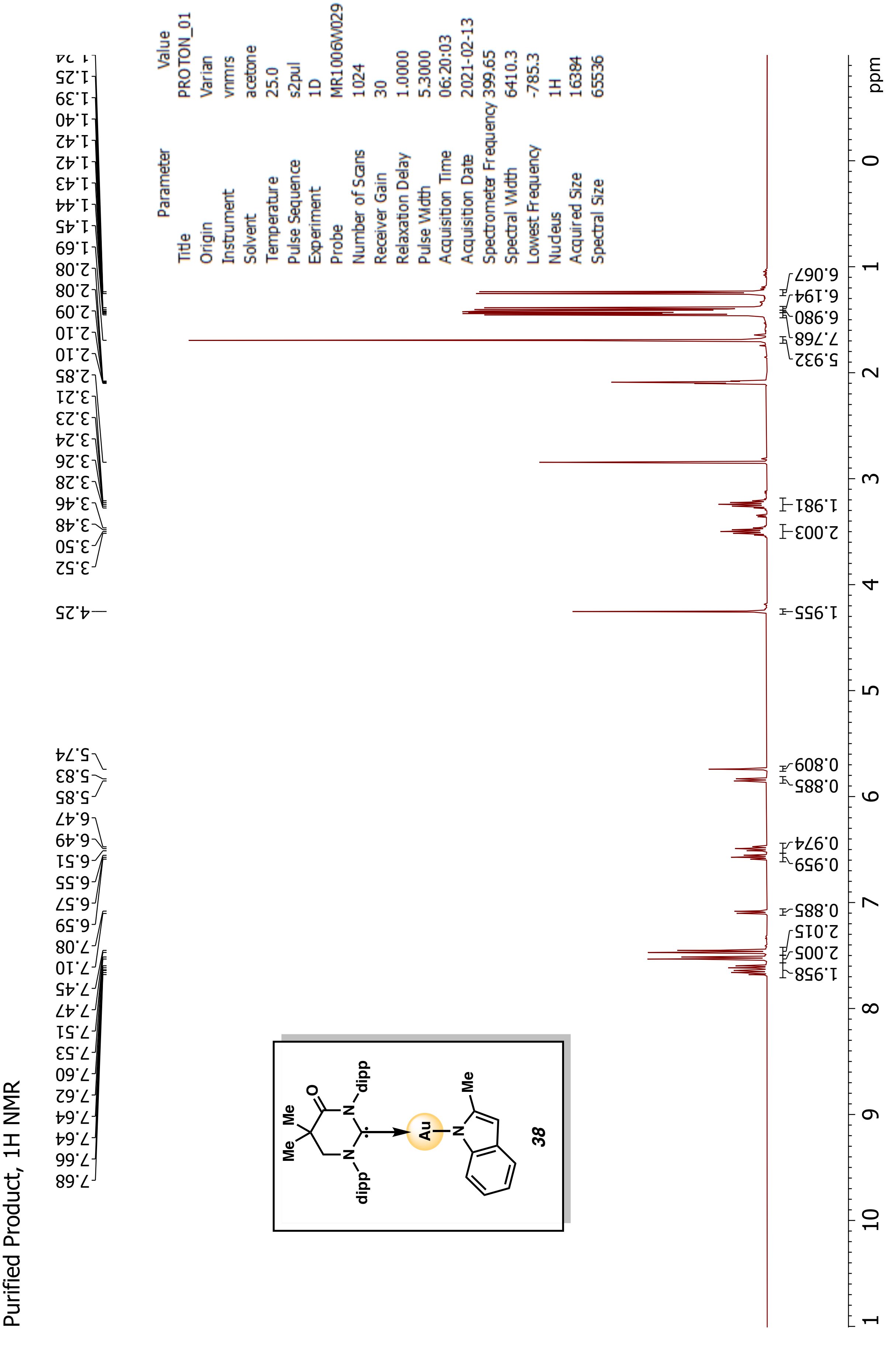
Purified Product, 1H NMR
Parameter Value
Title PROTON_01
Origin Varian
Instrument vnmrs
Solvent acetone
Temperature 25.0
Pulse Sequence s2pul
Experiment 1D
Probe MR1006W029
Number of Scans 1024
Receiver Gain 30
Relaxation Delay 1.0000
Pulse Width 5.3000
Acquisition Time 06:20:03
Acquisition Date 2021-02-13
Spectrometer Frequency 399.65
Spectral Width 6410.3
Lowest Frequency -785.3
Nucleus 1H
Acquired Size 16384
Spectral Size 65536
7.68 7.66 7.64 7.64 7.62 7.60 7.53 7.51 7.47 7.45 7.10 7.08 6.59 6.57 6.55 6.51 6.49 6.47 5.85 5.83 5.74
4.25
3.52 3.50 3.48 3.46 3.28 3.26 3.24 3.23 3.21 2.85 2.10 2.10 2.09 2.08 2.08 1.69 1.45 1.44 1.43 1.42 1.42 1.40 1.39 1.25 1.24
1.958 2.005 2.015 0.885 0.959 0.974 0.885 0.809 1.955 2.003 1.981 5.932 7.768 6.980 6.194 6.067
Me Me O N dipp N dipp Au N Me
38
ppm

Purified Product, 1H NMR

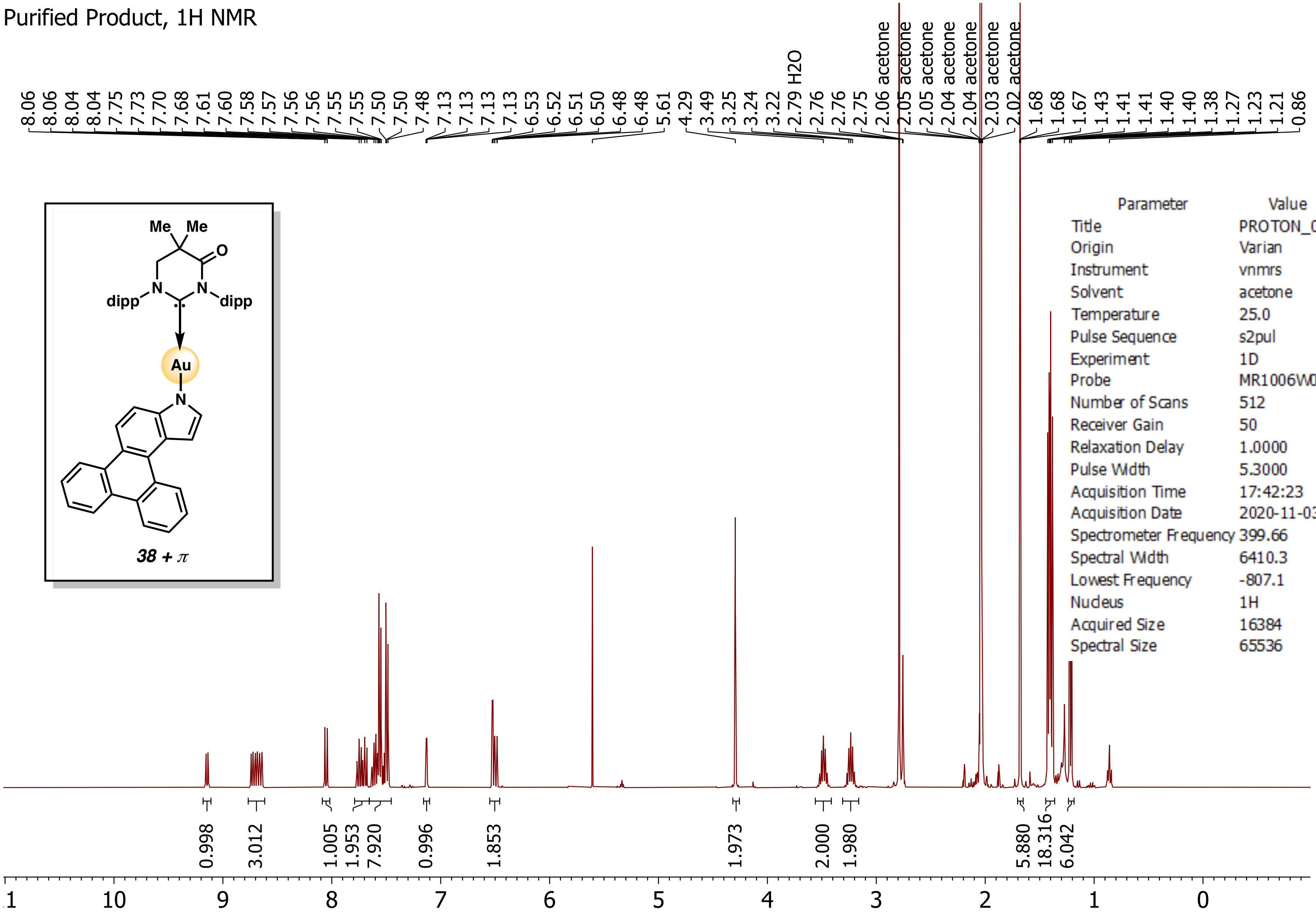

Parameter	Value
Title	PROTON_01
Origin	Varian
Instrument	vnmrs
Solvent	acetone
Temperature	25.0
Pulse Sequence	s2pul
Experiment	1D
Probe	MR1006W029
Number of Scans	512
Receiver Gain	50
Relaxation Delay	1.0000
Pulse Width	5.3000
Acquisition Time	17:42:23
Acquisition Date	2020-11-03
Spectrometer Frequency	399.66
Spectral Width	6410.3
Lowest Frequency	-807.1
Nucleus	1H
Acquired Size	16384
Spectral Size	65536

^{13}C NMR Spectra

Purified Product, 13C NMR

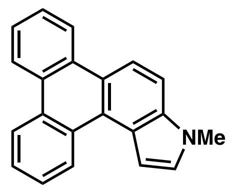

16

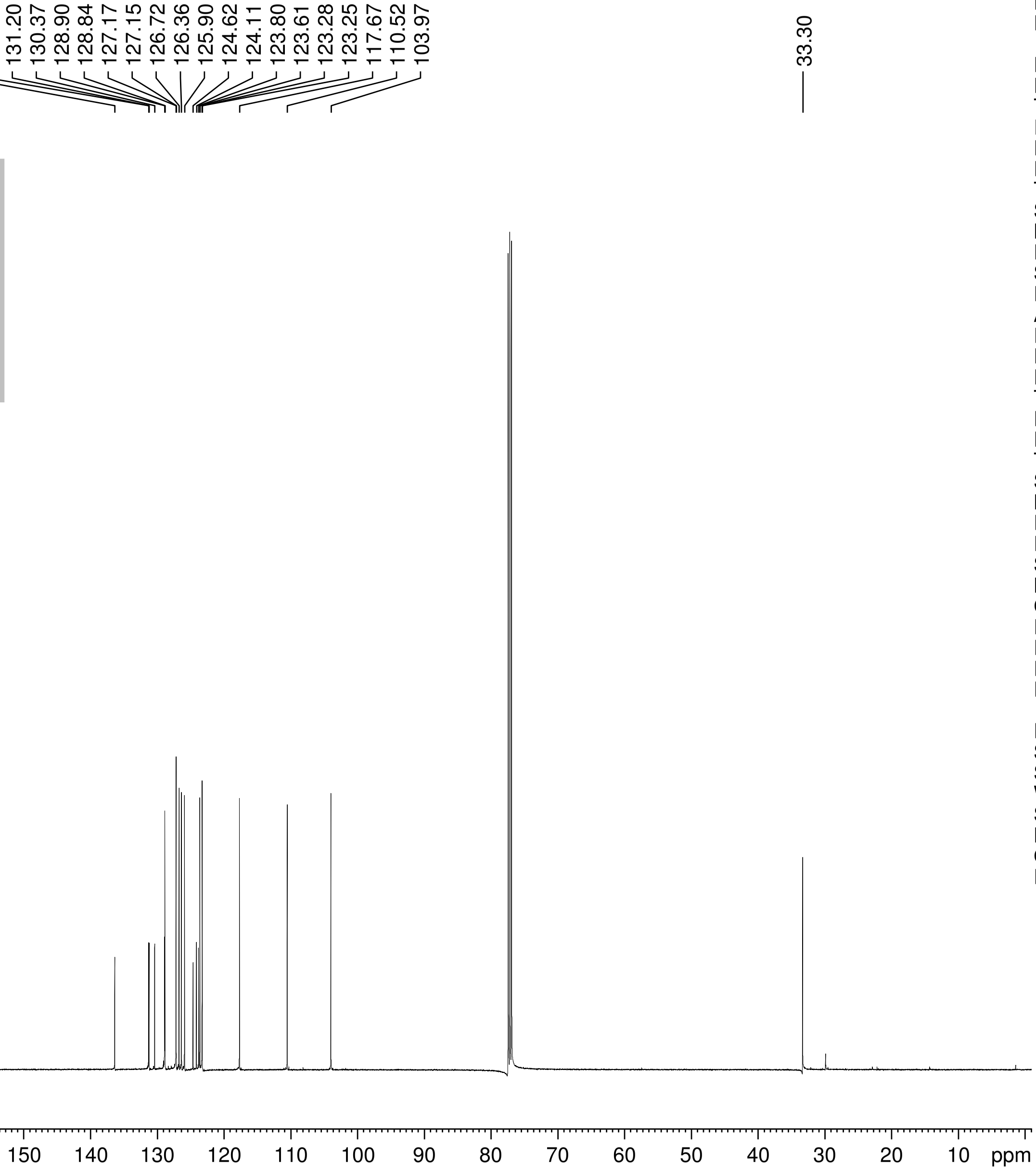

Current Data Parameters
NAME JVC-2018-087
EXPNO 11
PROCNO 1

F2 - Acquisition Parameters
Date_ 20180624
Time 18.05 h
INSTRUM av500
PROBHD Z119248_0002 (
PULPROG zgpg30
TD 65536
SOLVENT CDCl3
NS 368
DS 2
SWH 31250.000 Hz
FIDRES 0.953674 Hz
AQ 1.0485760 sec
RG 204.54
DW 16.000 usec
DE 18.00 usec
TE 298.0 K
D1 2.00000000 sec
D11 0.03000000 sec
TD0 1
SFO1 125.7722511 MHz
NUC1 13C
P1 10.50 usec
PLW1 23.00000000 W
SFO2 500.1330008 MHz
NUC2 1H
CPDPRG[2 waltz16
PCPD2 80.00 usec
PLW2 13.50000000 W
PLW12 0.21094000 W
PLW13 0.10610000 W

F2 - Processing parameters
SI 131072
SF 125.7577785 MHz
WDW EM
SSB 0
LB 1.00 Hz
GB 0
PC 1.40

Purified Product, 13C NMR

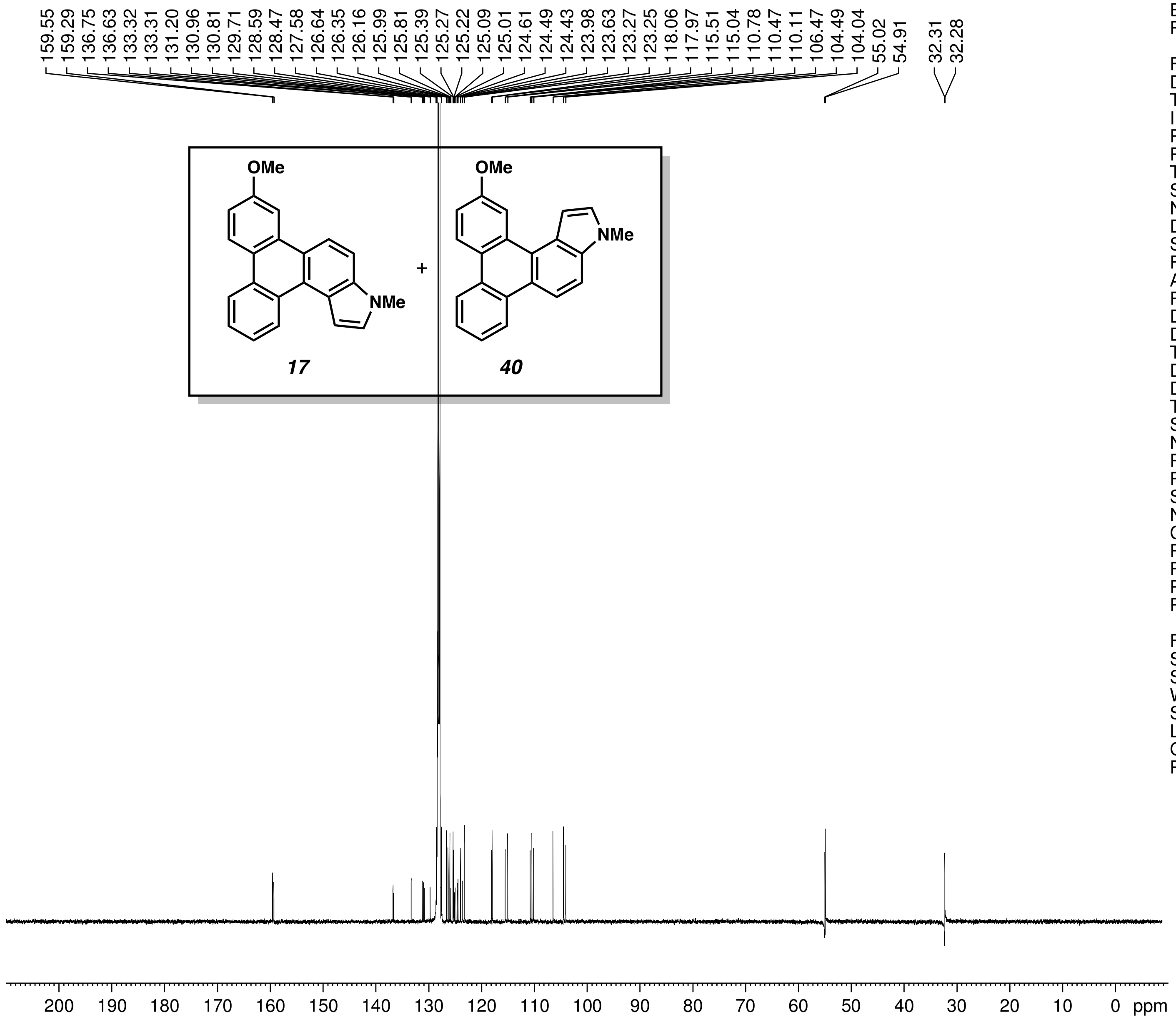

Current Data Parameters
NAME KAS-2018-052
EXPNO 2
PROCNO 1

F2 - Acquisition Parameters
Date_ 20180923
Time 17.42 h
INSTRUM av500
PROBHD Z119248_0002 (
PULPROG zgpg30
TD 65536
SOLVENT C6D6
NS 92
DS 2
SWH 31250.000 Hz
FIDRES 0.953674 Hz
AQ 1.0485760 sec
RG 14.67
DW 16.000 usec
DE 18.00 usec
TE 298.0 K
D1 2.00000000 sec
D11 0.03000000 sec
TD0 1
SFO1 125.7722511 MHz
NUC1 13C
P1 9.63 usec
PLW1 23.00000000 W
SFO2 500.1330008 MHz
NUC2 1H
CPDPRG[2 waltz16
PCPD2 80.00 usec
PLW2 13.50000000 W
PLW12 0.21094000 W
PLW13 0.13500001 W

F2 - Processing parameters
SI 131072
SF 125.7577435 MHz
WDW EM
SSB 0
LB 1.00 Hz
GB 0
PC 1.40

Purified Product, 13C NMR

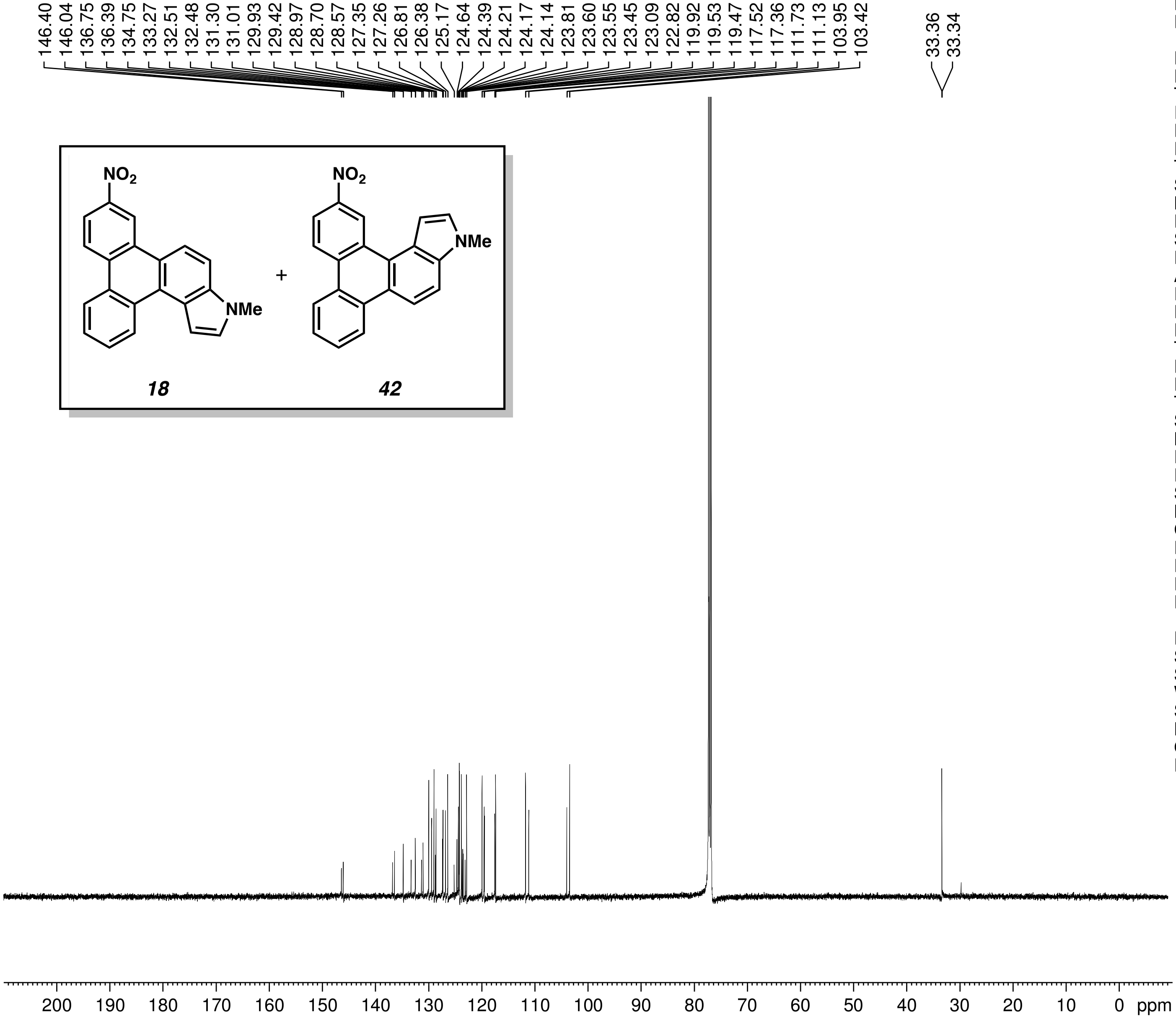

Current Data Parameters
NAME KAS-2019-014
EXPNO 2
PROCNO 1

F2 - Acquisition Parameters
Date_ 20190122
Time 16.27 h
INSTRUM av500
PROBHD Z119248_0002 (
PULPROG zgpg30
TD 65536
SOLVENT CDCl3
NS 529
DS 2
SWH 31250.000 Hz
FIDRES 0.953674 Hz
AQ 1.0485760 sec
RG 204.54
DW 16.000 usec
DE 28.00 usec
TE 298.0 K
D1 2.00000000 sec
D11 0.03000000 sec
TD0 1
SFO1 125.7722511 MHz
NUC1 13C
P1 10.50 usec
PLW1 23.00000000 W
SFO2 500.1330008 MHz
NUC2 1H
CPDPRG[2 waltz16
PCPD2 80.00 usec
PLW2 13.50000000 W
PLW12 0.21094000 W
PLW13 0.10610000 W

F2 - Processing parameters
SI 131072
SF 125.7577892 MHz
WDW EM
SSB 0
LB 1.00 Hz
GB 0
PC 1.40

Purified Product, 13C NMR

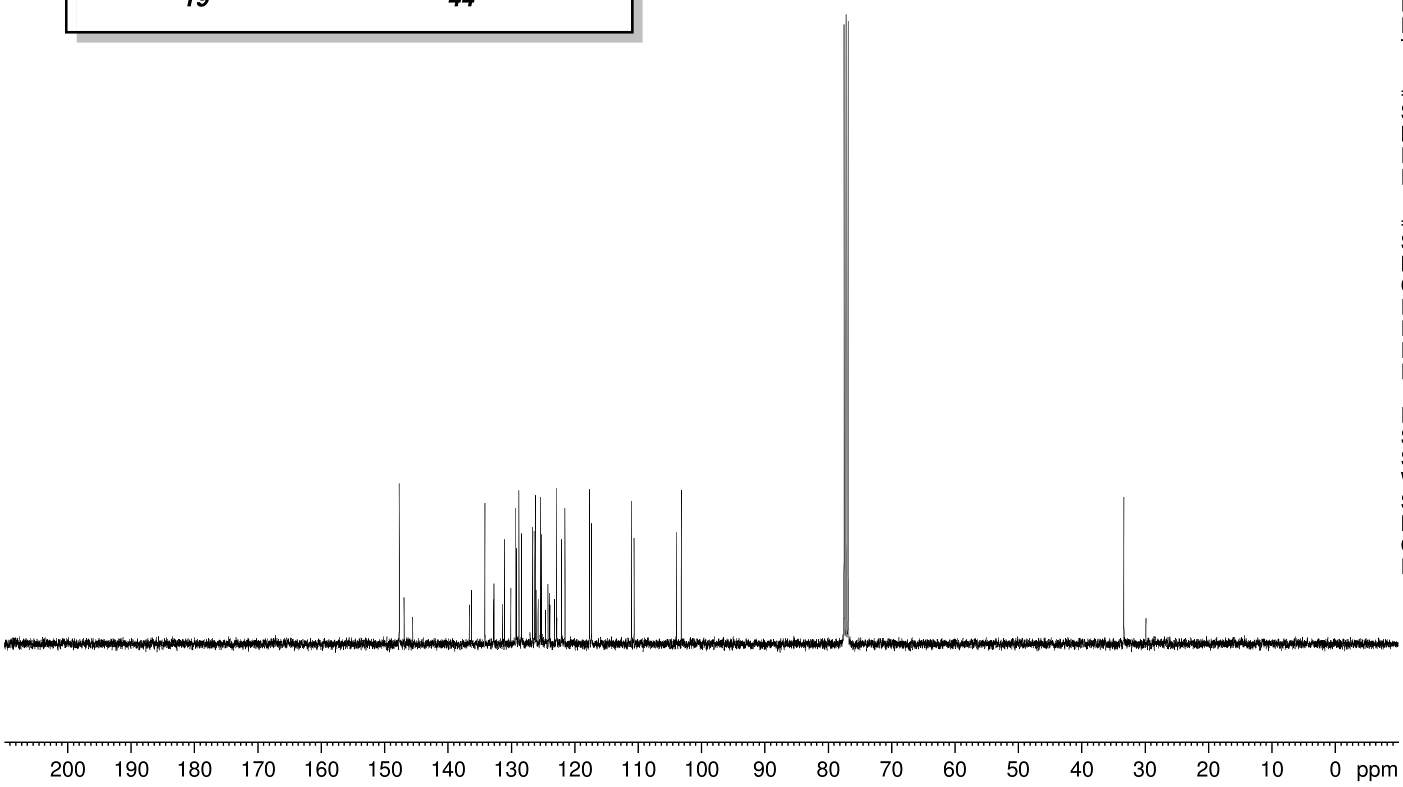

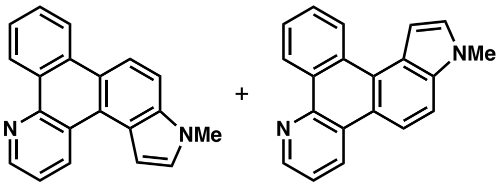

19 **44**

Current Data Parameters
NAME JVC-2018-105
EXPNO 4
PROCNO 1

F2 - Acquisition Parameters
Date_ 20180725
Time 23.55
INSTRUM av400
PROBHD 5 mm PABBO BB/
PULPROG zgpg30
TD 65536
SOLVENT CDCl3
NS 352
DS 0
SWH 25252.525 Hz
FIDRES 0.385323 Hz
AQ 1.2976128 sec
RG 189.85
DW 19.800 usec
DE 6.50 usec
TE 299.1 K
D1 2.00000000 sec
D11 0.03000000 sec
TD0 1

======== CHANNEL f1 ======
SFO1 100.6243395 MHz
NUC1 13C
P1 10.00 usec
PLW1 52.00000000 W

======== CHANNEL f2 ======
SFO2 400.1324008 MHz
NUC2 1H
CPDPRG[2 waltz16
PCPD2 90.00 usec
PLW2 13.00000000 W
PLW12 0.36111000 W
PLW13 0.29249999 W

F2 - Processing parameters
SI 65536
SF 100.6127603 MHz
WDW EM
SSB 0
LB 1.00 Hz
GB 0
PC 1.40

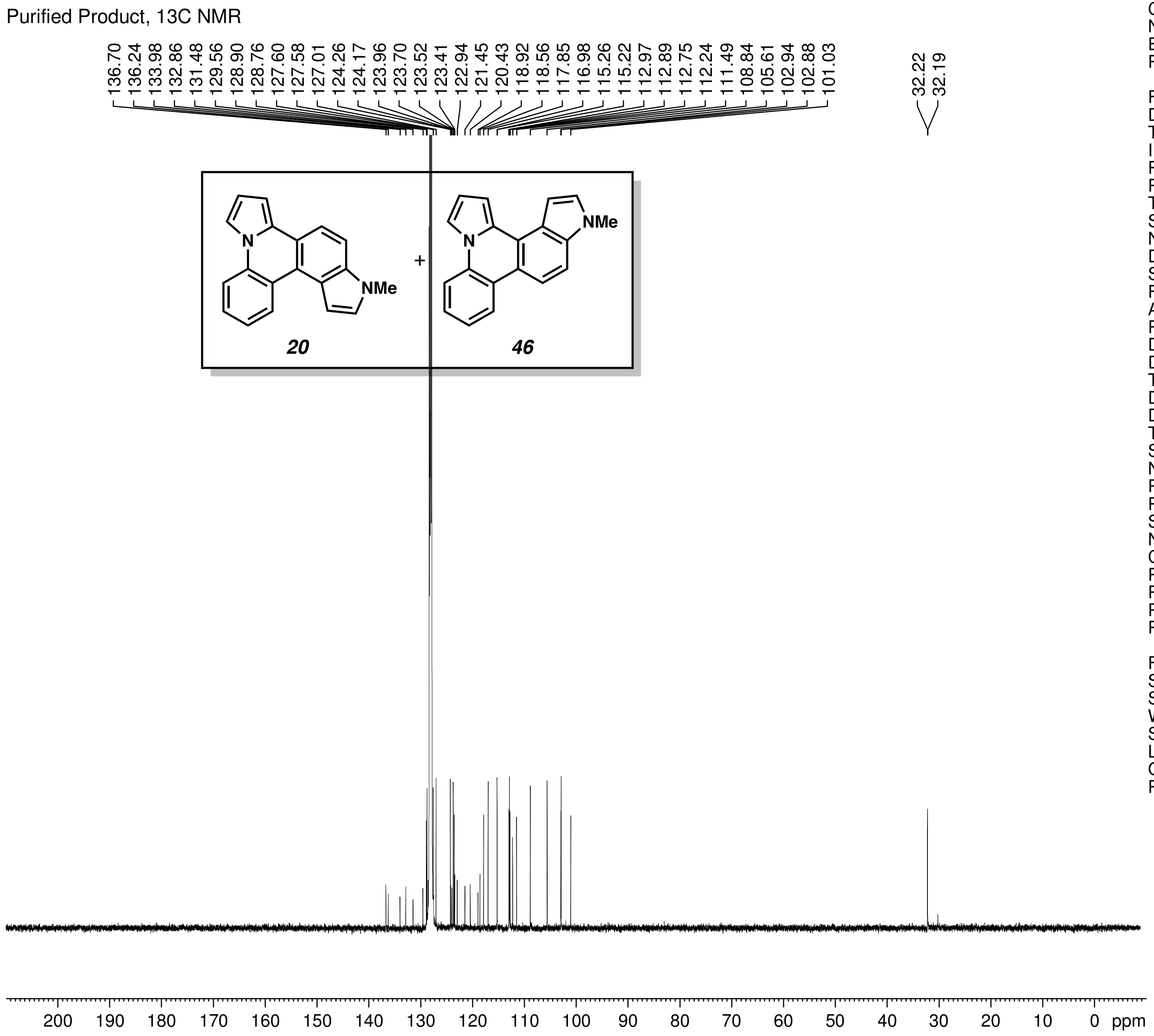

Current Data Parameters
NAME KAS-2019-015
EXPNO 2
PROCNO 1

F2 - Acquisition Parameters
Date_ 20190122
Time 20.13 h
INSTRUM av500
PROBHD Z119248_0002 (
PULPROG zgpg30
TD 65536
SOLVENT C6D6
NS 147
DS 2
SWH 31250.000 Hz
FIDRES 0.953674 Hz
AQ 1.0485760 sec
RG 204.54
DW 16.000 usec
DE 28.00 usec
TE 298.0 K
D1 2.00000000 sec
D11 0.03000000 sec
TD0 1
SFO1 125.7722511 MHz
NUC1 13C
P1 10.50 usec
PLW1 23.00000000 W
SFO2 500.1330008 MHz
NUC2 1H
CPDPRG[2 waltz16
PCPD2 80.00 usec
PLW2 13.50000000 W
PLW12 0.21094000 W
PLW13 0.10610000 W

F2 - Processing parameters
SI 131072
SF 125.7577434 MHz
WDW EM
SSB 0
LB 1.00 Hz
GB 0
PC 1.40

Purified Product, 13C NMR

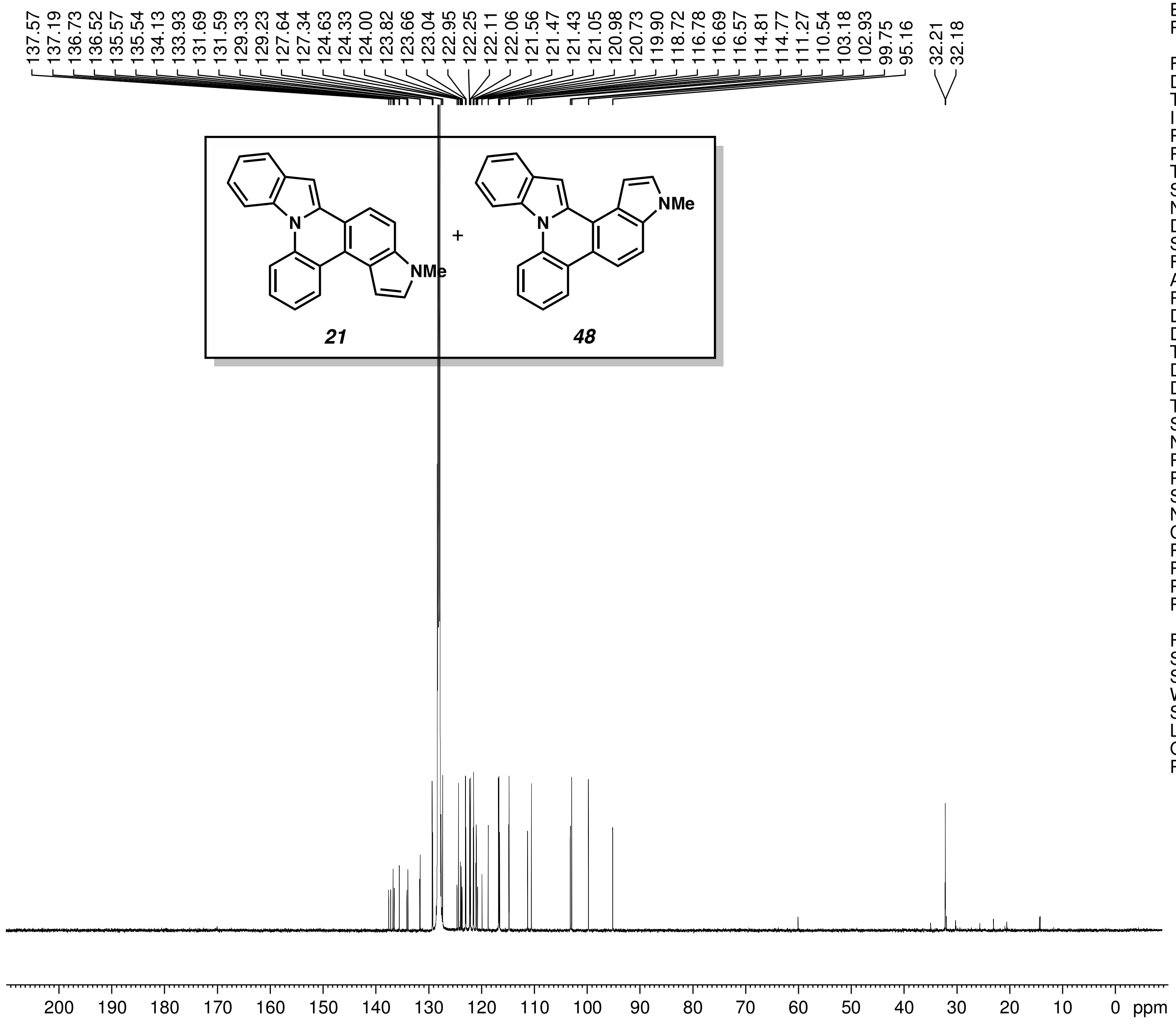

Current Data Parameters
NAME JVC-2019-010
EXPNO 16
PROCNO 1

F2 - Acquisition Parameters
Date_ 20190125
Time 15.05 h
INSTRUM av500
PROBHD Z119248_0002 (
PULPROG zgpg30
TD 65536
SOLVENT C6D6
NS 234
DS 2
SWH 31250.000 Hz
FIDRES 0.953674 Hz
AQ 1.0485760 sec
RG 204.54
DW 16.000 usec
DE 28.00 usec
TE 298.0 K
D1 2.00000000 sec
D11 0.03000000 sec
TD0 1
SFO1 125.7722511 MHz
NUC1 13C
P1 10.50 usec
PLW1 23.00000000 W
SFO2 500.1330008 MHz
NUC2 1H
CPDPRG[2 waltz16
PCPD2 80.00 usec
PLW2 13.50000000 W
PLW12 0.21094000 W
PLW13 0.10610000 W

F2 - Processing parameters
SI 131072
SF 125.7577432 MHz
WDW EM
SSB 0
LB 1.00 Hz
GB 0
PC 1.40

Purified Product, 13C NMR

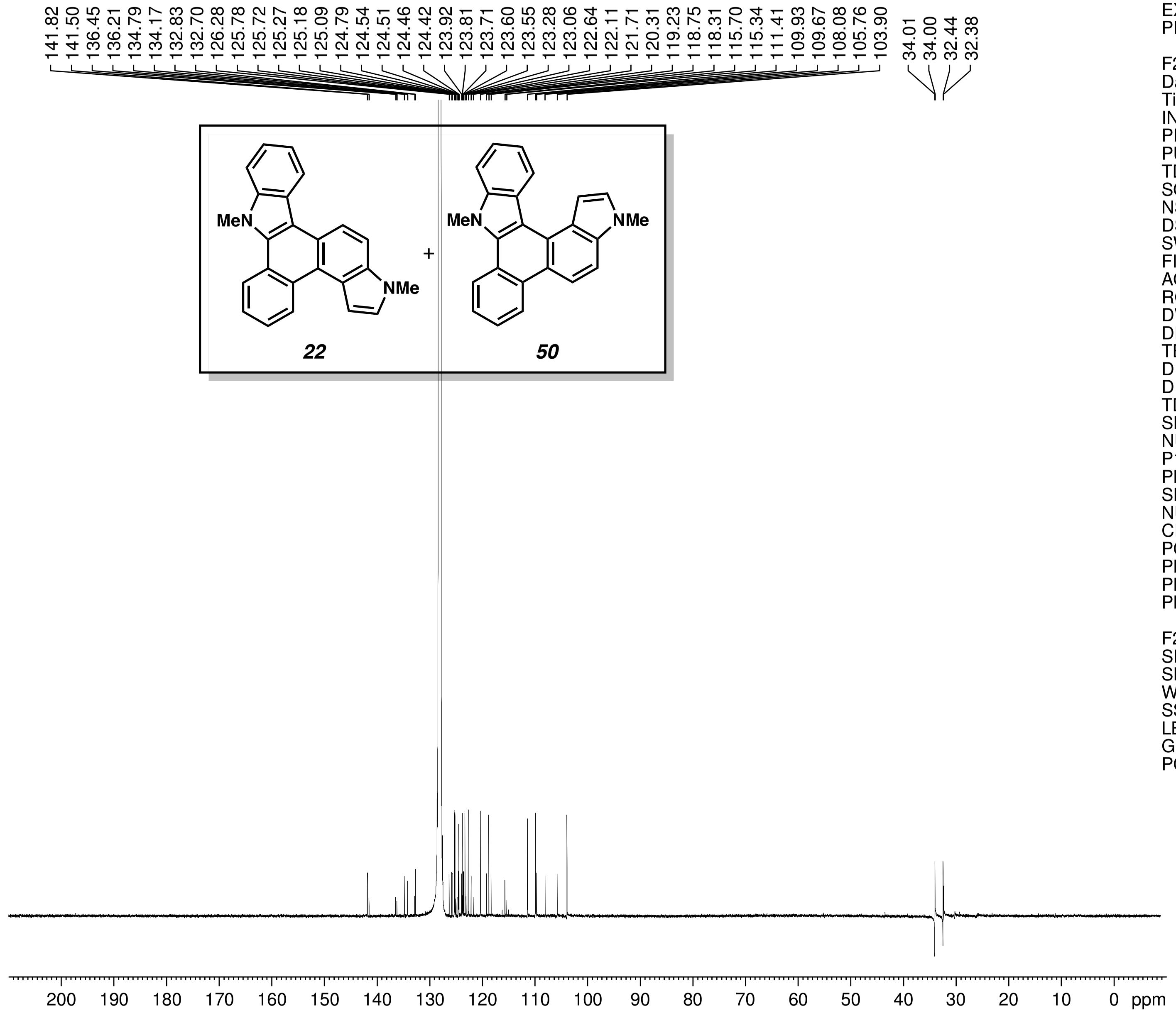

Current Data Parameters
NAME JVC-2018-107-3-2D
EXPNO 4
PROCNO 1

F2 - Acquisition Parameters
Date_ 20180820
Time 2.52 h
INSTRUM av500
PROBHD Z119248_0002 (
PULPROG zgpg30
TD 65536
SOLVENT C6D6
NS 4600
DS 2
SWH 31250.000 Hz
FIDRES 0.953674 Hz
AQ 1.0485760 sec
RG 204.54
DW 16.000 usec
DE 18.00 usec
TE 298.0 K
D1 2.00000000 sec
D11 0.03000000 sec
TD0 1
SFO1 125.7722511 MHz
NUC1 13C
P1 10.50 usec
PLW1 23.00000000 W
SFO2 500.1330008 MHz
NUC2 1H
CPDPRG[2 waltz16
PCPD2 80.00 usec
PLW2 13.50000000 W
PLW12 0.21094000 W
PLW13 0.10610000 W

F2 - Processing parameters
SI 131072
SF 125.7577430 MHz
WDW EM
SSB 0
LB 1.00 Hz
GB 0
PC 1.40

Purified Product, 13C NMR

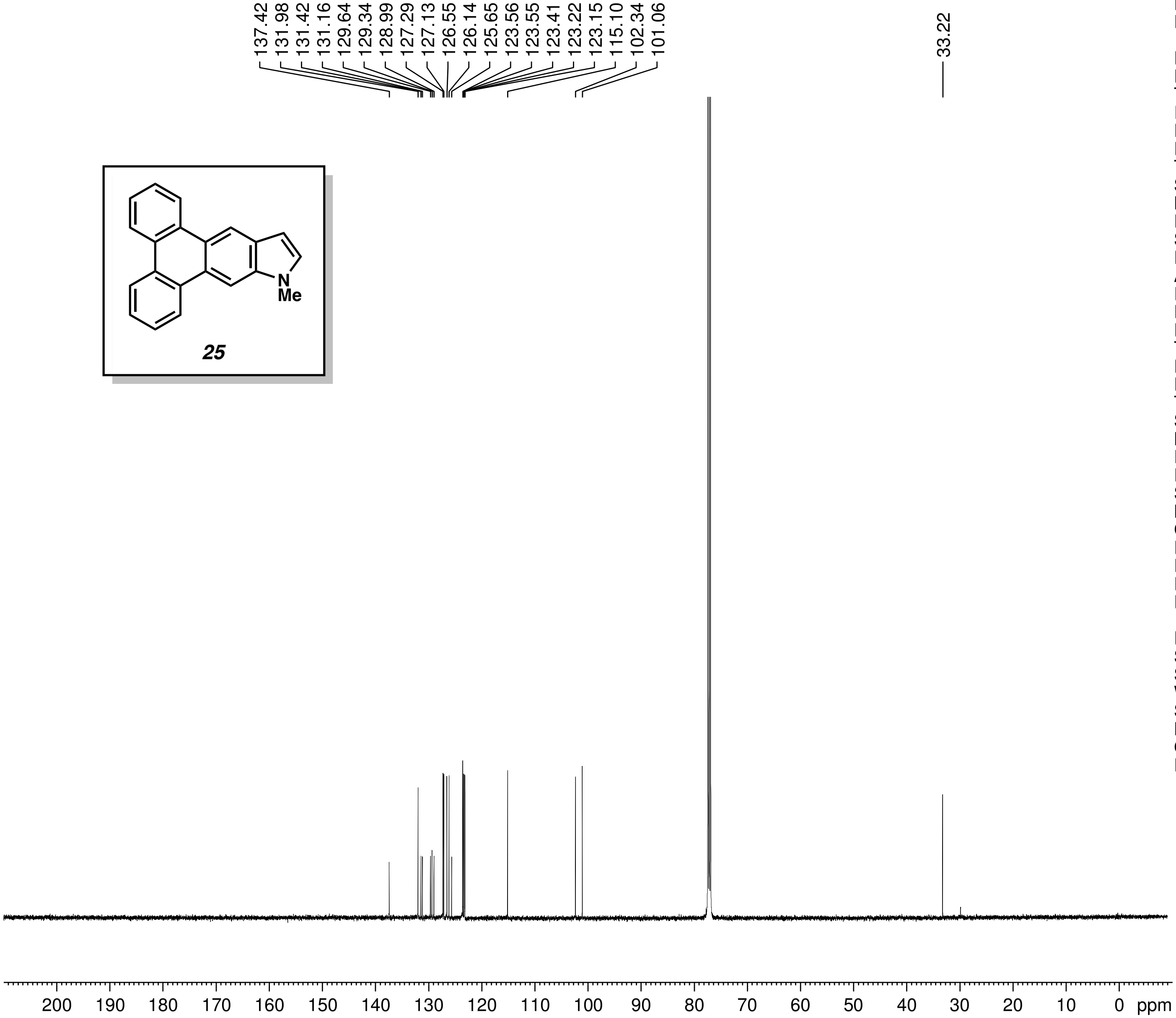

Current Data Parameters
NAME RBS-2019-012
EXPNO 4
PROCNO 1

F2 - Acquisition Parameters
Date_ 20190127
Time 12.59 h
INSTRUM av500
PROBHD Z119248_0002 (
PULPROG zgpg30
TD 65536
SOLVENT CDCl3
NS 240
DS 2
SWH 31250.000 Hz
FIDRES 0.953674 Hz
AQ 1.0485760 sec
RG 204.54
DW 16.000 usec
DE 28.00 usec
TE 298.0 K
D1 2.00000000 sec
D11 0.03000000 sec
TD0 1
SFO1 125.7722511 MHz
NUC1 13C
P1 10.50 usec
PLW1 23.00000000 W
SFO2 500.1330008 MHz
NUC2 1H
CPDPRG[2 waltz16
PCPD2 80.00 usec
PLW2 13.50000000 W
PLW12 0.21094000 W
PLW13 0.10610000 W

F2 - Processing parameters
SI 131072
SF 125.7577725 MHz
WDW EM
SSB 0
LB 1.00 Hz
GB 0
PC 1.40

Purified Product, 13C NMR

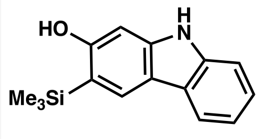

28

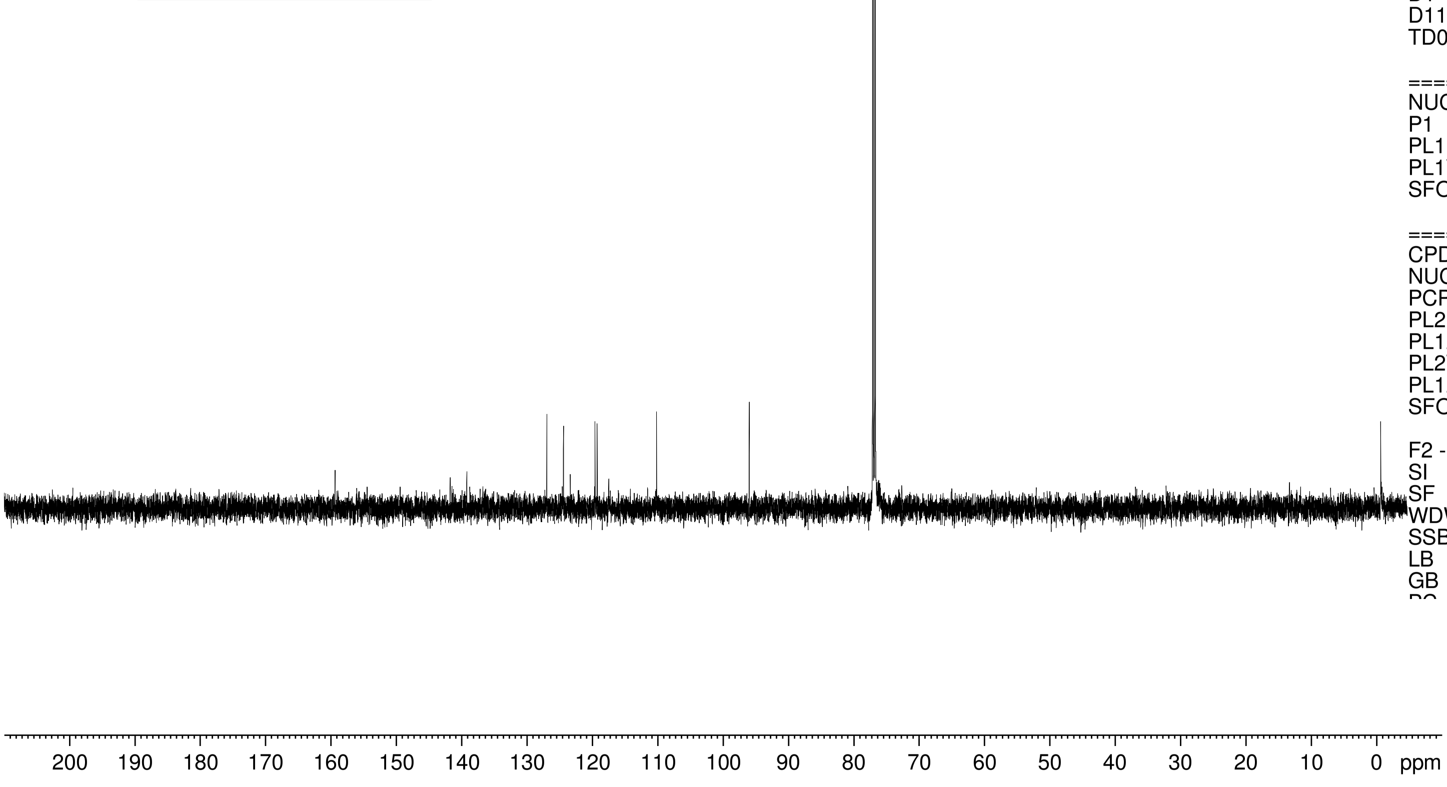

Current Data Parameters
NAME KAS-2020-017
EXPNO 3
PROCNO 999

F2 - Acquisition Parameters
Date_ 20200121
Time 10.32
INSTRUM av600
PROBHD 5 mm BB5
PULPROG zgdc30
TD 65536
SOLVENT CDCl3
NS 181
DS 0
SWH 37593.984 Hz
FIDRES 0.573639 Hz
AQ 0.8716288 sec
RG 18390.4
DW 13.300 usec
DE 6.50 usec
TE 295.9 K
D1 2.00000000 sec
D11 0.03000000 sec
TD0 1

======== CHANNEL f1 =====
NUC1 13C
P1 10.50 usec
PL1 0 dB
PL1W 75.35659027 W
SFO1 150.9209173 MHz

======== CHANNEL f2 =====
CPDPRG[2 waltz16
NUC2 1H
PCPD2 100.00 usec
PL2 -1.00 dB
PL12 14.65 dB
PL2W 31.62277603 W
PL12W 0.86099380 W
SFO2 600.1336008 MHz

F2 - Processing parameters
SI 65536
SF 150.9028319 MHz
WDW EM
SSB 0
LB 1.00 Hz
GB 0
[illegible]

Purified Product, 13C NMR

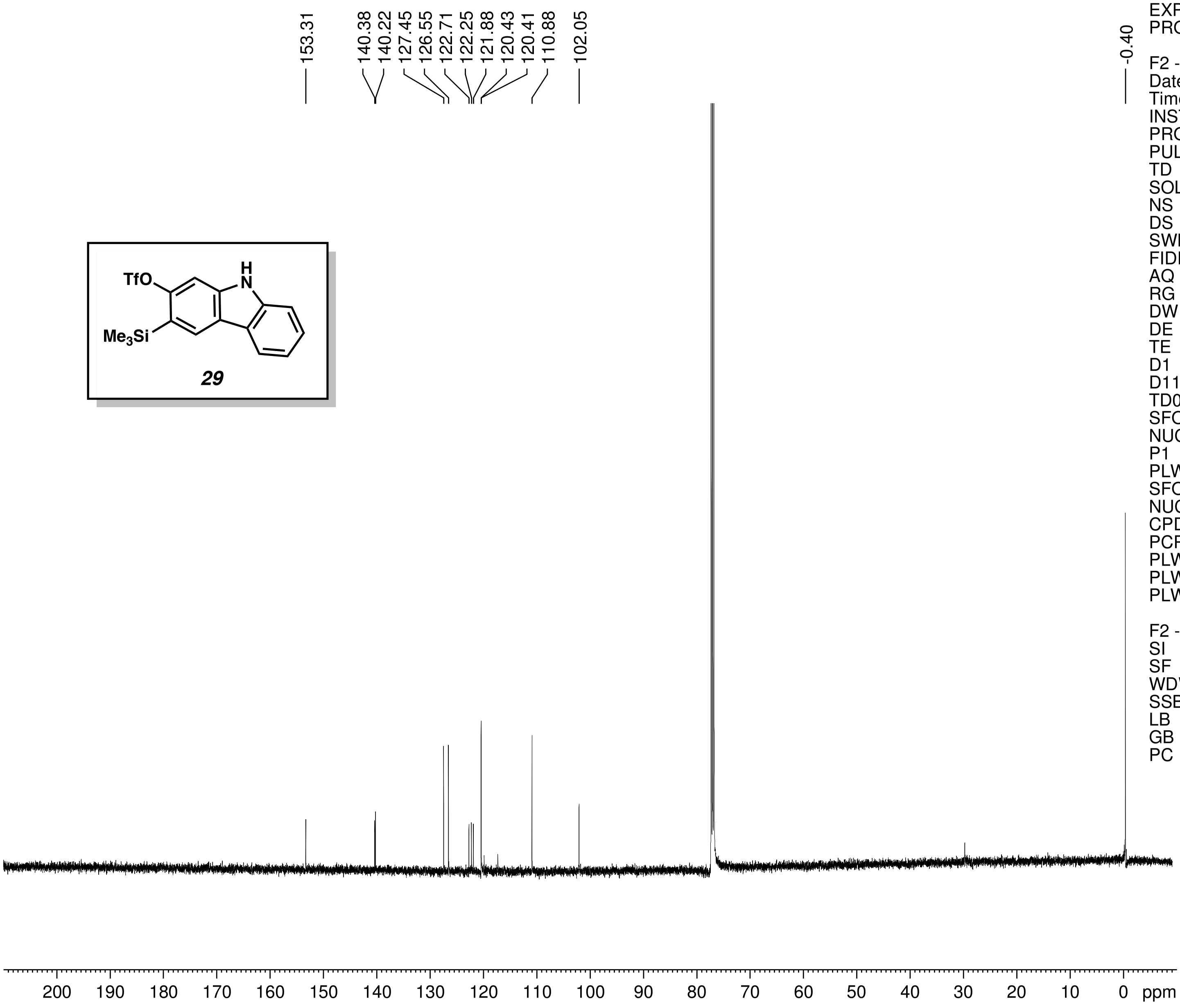

Current Data Parameters
NAME KAS-2021-000
EXPNO 201
PROCNO 1

F2 - Acquisition Parameters
Date_ 20210728
Time 15.26 h
INSTRUM av500
PROBHD Z119248_0002 (
PULPROG zgpg30
TD 65536
SOLVENT CDCl3
NS 193
DS 2
SWH 31250.000 Hz
FIDRES 0.953674 Hz
AQ 1.0485760 sec
RG 204.54
DW 16.000 usec
DE 28.00 usec
TE 298.0 K
D1 2.00000000 sec
D11 0.03000000 sec
TD0 1
SFO1 125.7722511 MHz
NUC1 13C
P1 10.50 usec
PLW1 23.00000000 W
SFO2 500.1330008 MHz
NUC2 1H
CPDPRG[2 waltz16
PCPD2 80.00 usec
PLW2 13.50000000 W
PLW12 0.21094000 W
PLW13 0.10610000 W

F2 - Processing parameters
SI 131072
SF 125.7577892 MHz
WDW EM
SSB 0
LB 1.00 Hz
GB 0
PC 1.40

Purified Product, 13C NMR

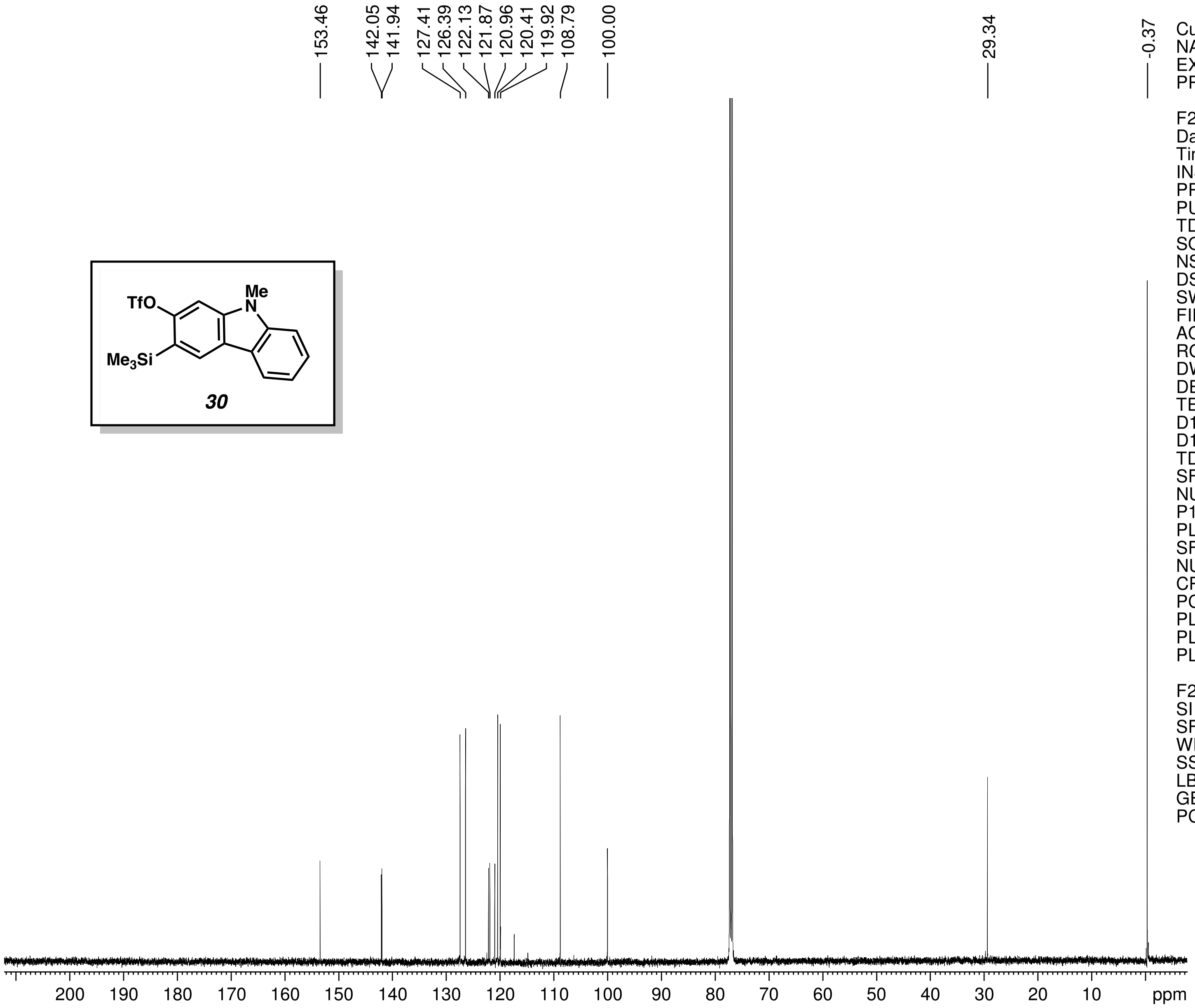

30

Current Data Parameters
NAME KAS-2021-000
EXPNO 21
PROCNO 1

F2 - Acquisition Parameters
Date_ 20210713
Time 18.47 h
INSTRUM av500
PROBHD Z119248_0002 (
PULPROG zgpg30
TD 65536
SOLVENT CDCl3
NS 46
DS 2
SWH 31250.000 Hz
FIDRES 0.953674 Hz
AQ 1.0485760 sec
RG 204.54
DW 16.000 usec
DE 28.00 usec
TE 298.0 K
D1 2.00000000 sec
D11 0.03000000 sec
TD0 1
SFO1 125.7722511 MHz
NUC1 13C
P1 10.50 usec
PLW1 23.00000000 W
SFO2 500.1330008 MHz
NUC2 1H
CPDPRG[2 waltz16
PCPD2 80.00 usec
PLW2 13.50000000 W
PLW12 0.21094000 W
PLW13 0.10610000 W

F2 - Processing parameters
SI 131072
SF 125.7577892 MHz
WDW EM
SSB 0
LB 1.00 Hz
GB 0
PC 1.40

Purified Product, 13C NMR

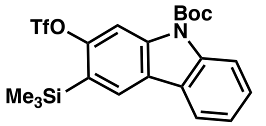

31

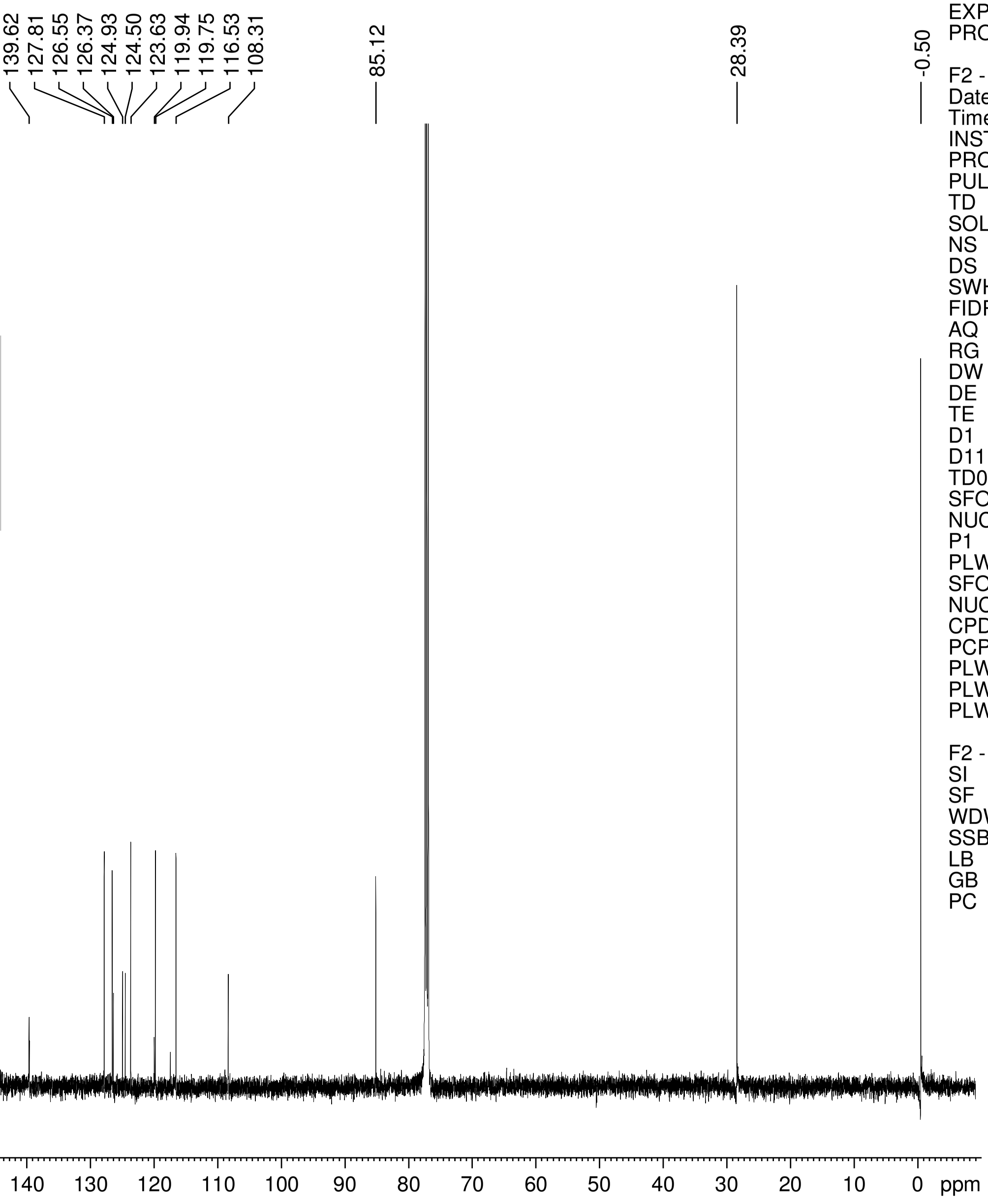

Current Data Parameters
NAME KAS-2021-000
EXPNO 111
PROCNO 1

F2 - Acquisition Parameters
Date_ 20210722
Time 16.27 h
INSTRUM av500
PROBHD Z119248_0002 (
PULPROG zgpg30
TD 65536
SOLVENT CDCl3
NS 85
DS 2
SWH 31250.000 Hz
FIDRES 0.953674 Hz
AQ 1.0485760 sec
RG 204.54
DW 16.000 usec
DE 28.00 usec
TE 298.0 K
D1 2.00000000 sec
D11 0.03000000 sec
TD0 1
SFO1 125.7722511 MHz
NUC1 13C
P1 10.50 usec
PLW1 23.00000000 W
SFO2 500.1330008 MHz
NUC2 1H
CPDPRG[2 waltz16
PCPD2 80.00 usec
PLW2 13.50000000 W
PLW12 0.21094000 W
PLW13 0.10610000 W

F2 - Processing parameters
SI 131072
SF 125.7577769 MHz
WDW EM
SSB 0
LB 1.00 Hz
GB 0
PC 1.40

Purified Product, 13C NMR

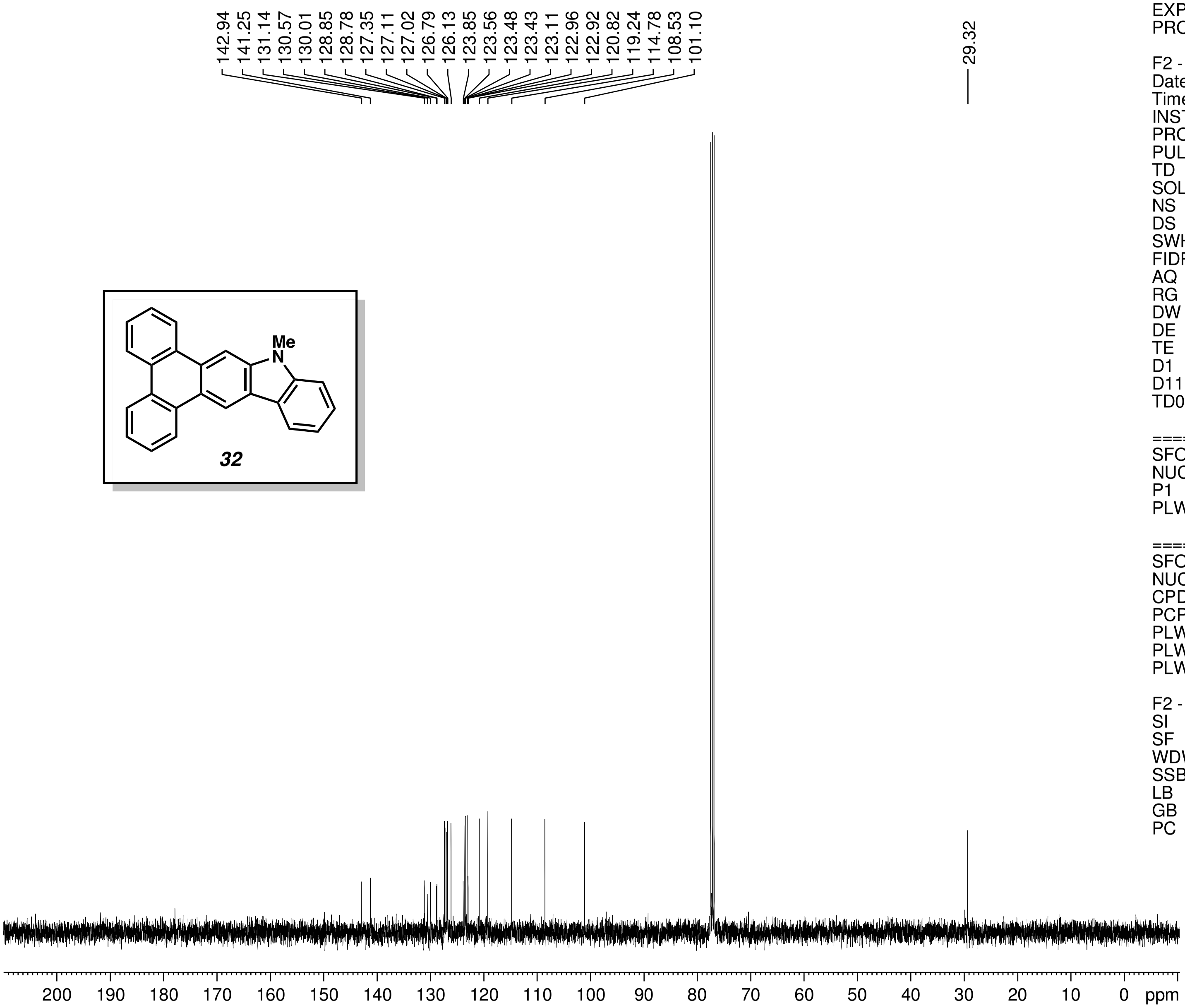

32

Current Data Parameters
NAME RBS-2018-171
EXPNO 5
PROCNO 1

F2 - Acquisition Parameters
Date_ 20181106
Time 16.21
INSTRUM av400
PROBHD 5 mm PABBO BB/
PULPROG zgpg30
TD 65536
SOLVENT CDCl3
NS 88
DS 0
SWH 25252.525 Hz
FIDRES 0.385323 Hz
AQ 1.2976128 sec
RG 189.85
DW 19.800 usec
DE 6.50 usec
TE 299.2 K
D1 2.00000000 sec
D11 0.03000000 sec
TD0 1

======== CHANNEL f1 =====
SFO1 100.6243395 MHz
NUC1 13C
P1 10.00 usec
PLW1 52.00000000 W

======== CHANNEL f2 =====
SFO2 400.1324008 MHz
NUC2 1H
CPDPRG[2 waltz16
PCPD2 90.00 usec
PLW2 13.00000000 W
PLW12 0.36111000 W
PLW13 0.29249999 W

F2 - Processing parameters
SI 65536
SF 100.6127581 MHz
WDW EM
SSB 0
LB 1.00 Hz
GB 0
PC 1.40

Purified Product, 13C NMR

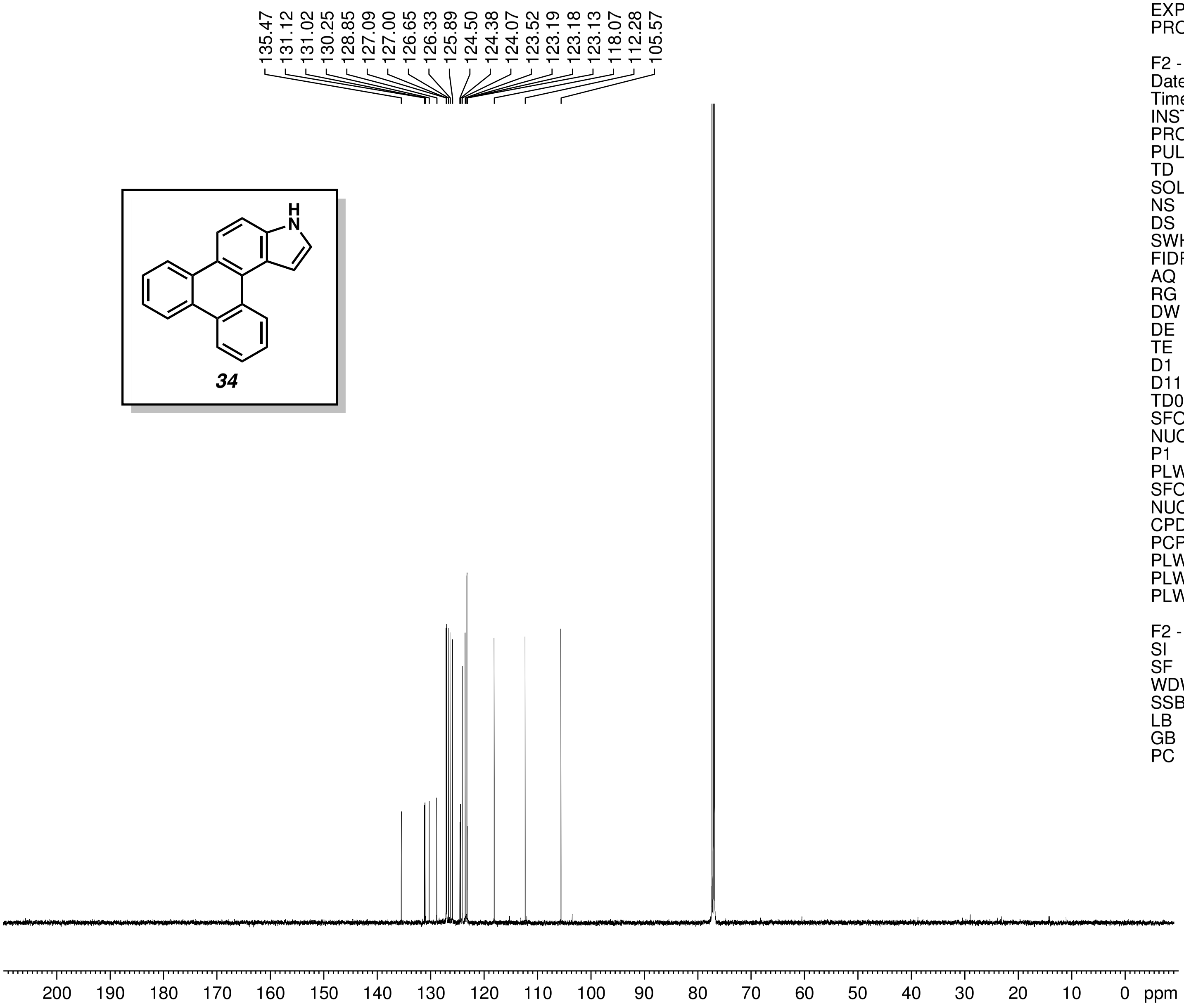

Current Data Parameters
NAME KAS-2021-000
EXPNO 33
PROCNO 1

F2 - Acquisition Parameters
Date_ 20210714
Time 14.01 h
INSTRUM av500
PROBHD Z119248_0002 (
PULPROG zgpg30
TD 65536
SOLVENT CDCl3
NS 32
DS 2
SWH 31250.000 Hz
FIDRES 0.953674 Hz
AQ 1.0485760 sec
RG 204.54
DW 16.000 usec
DE 28.00 usec
TE 298.0 K
D1 2.00000000 sec
D11 0.03000000 sec
TD0 1
SFO1 125.7722511 MHz
NUC1 13C
P1 10.50 usec
PLW1 23.00000000 W
SFO2 500.1330008 MHz
NUC2 1H
CPDPRG[2 waltz16
PCPD2 80.00 usec
PLW2 13.50000000 W
PLW12 0.21094000 W
PLW13 0.10610000 W

F2 - Processing parameters
SI 131072
SF 125.7577907 MHz
WDW EM
SSB 0
LB 1.00 Hz
GB 0
PC 1.40

Purified Material, 13C NMR

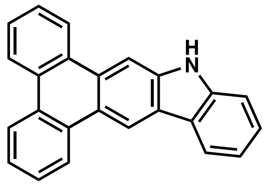

35

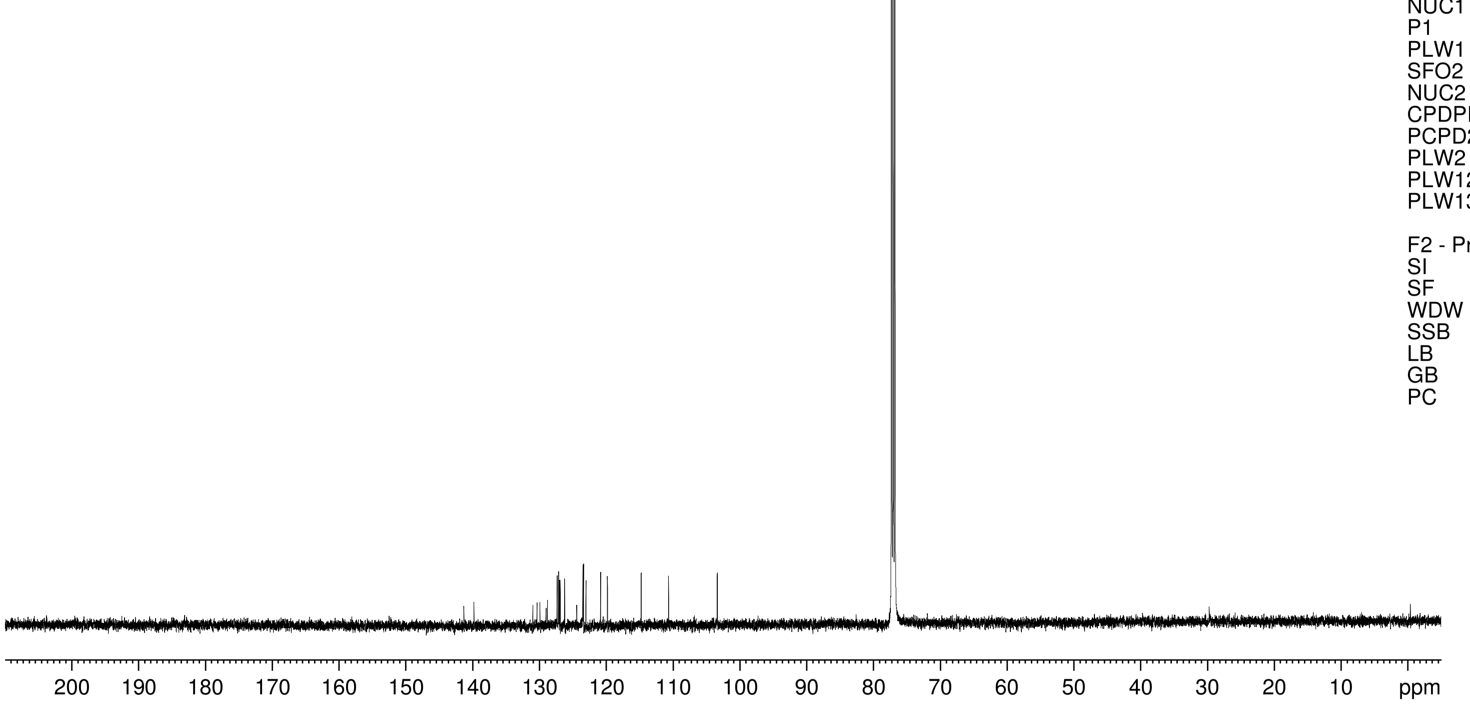

Current Data Parameters
NAME KAS-2021-000
EXPNO 6
PROCNO 1

F2 - Acquisition Parameters
Date_ 20210405
Time 11.06 h
INSTRUM av500
PROBHD Z119248_0002 (
PULPROG zgpg30
TD 65536
SOLVENT CDCl3
NS 193
DS 2
SWH 31250.000 Hz
FIDRES 0.953674 Hz
AQ 1.0485760 sec
RG 204.54
DW 16.000 usec
DE 28.00 usec
TE 298.0 K
D1 2.00000000 sec
D11 0.03000000 sec
TD0 1
SFO1 125.7722511 MHz
NUC1 13C
P1 10.50 usec
PLW1 23.00000000 W
SFO2 500.1330008 MHz
NUC2 1H
CPDPRG[2 waltz16
PCPD2 80.00 usec
PLW2 13.50000000 W
PLW12 0.21094000 W
PLW13 0.10610000 W

F2 - Processing parameters
SI 131072
SF 125.7577892 MHz
WDW EM
SSB 0
LB 1.00 Hz
GB 0
PC 1.40

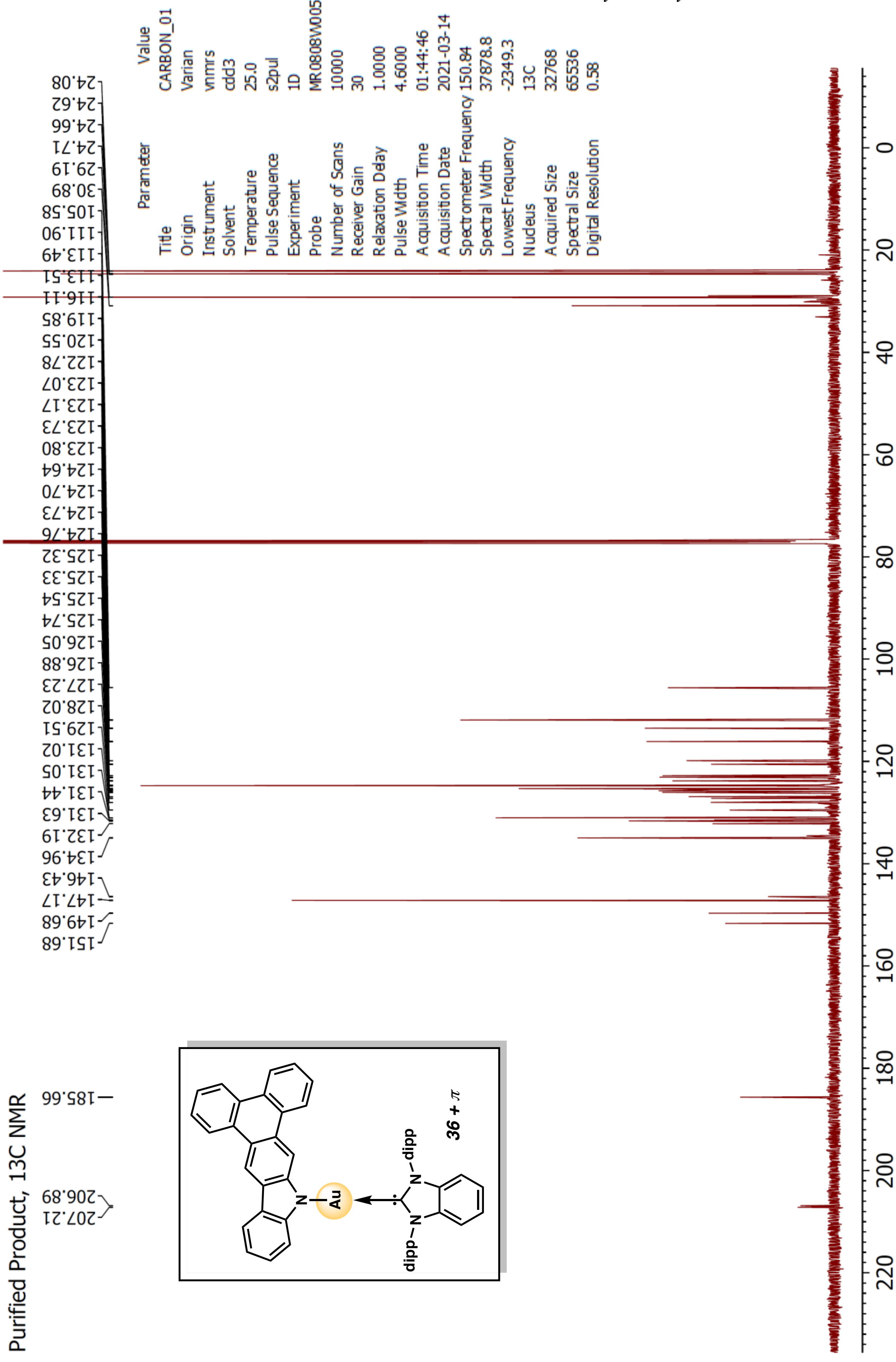
Purified Product, 13C NMR
207.21
206.89
185.66
151.68
149.68
147.17
146.43
134.96
132.19
131.63
131.44
131.05
131.02
129.51
128.02
127.23
126.88
126.05
125.74
125.54
125.33
125.32
124.76
124.73
124.70
124.64
123.80
123.73
123.17
123.07
122.78
120.55
119.85
116.11
113.51
113.49
111.90
105.58
30.89
29.19
24.71
24.66
24.62
24.08
Parameter Value
Title CARBON_01
Origin Varian
Instrument vnmrs
Solvent cdcl3
Temperature 25.0
Pulse Sequence s2pul
Experiment 1D
Probe MR0808W005
Number of Scans 10000
Receiver Gain 30
Relaxation Delay 1.0000
Pulse Width 4.6000
Acquisition Time 01:44:46
Acquisition Date 2021-03-14
Spectrometer Frequency 150.84
Spectral Width 37878.8
Lowest Frequency -2349.3
Nucleus 13C
Acquired Size 32768
Spectral Size 65536
Digital Resolution 0.58
N
Au
dipp
36 + π
220
200
180
160
140
120
100
80
60
40
20
0

Purified Product, 13C NMR

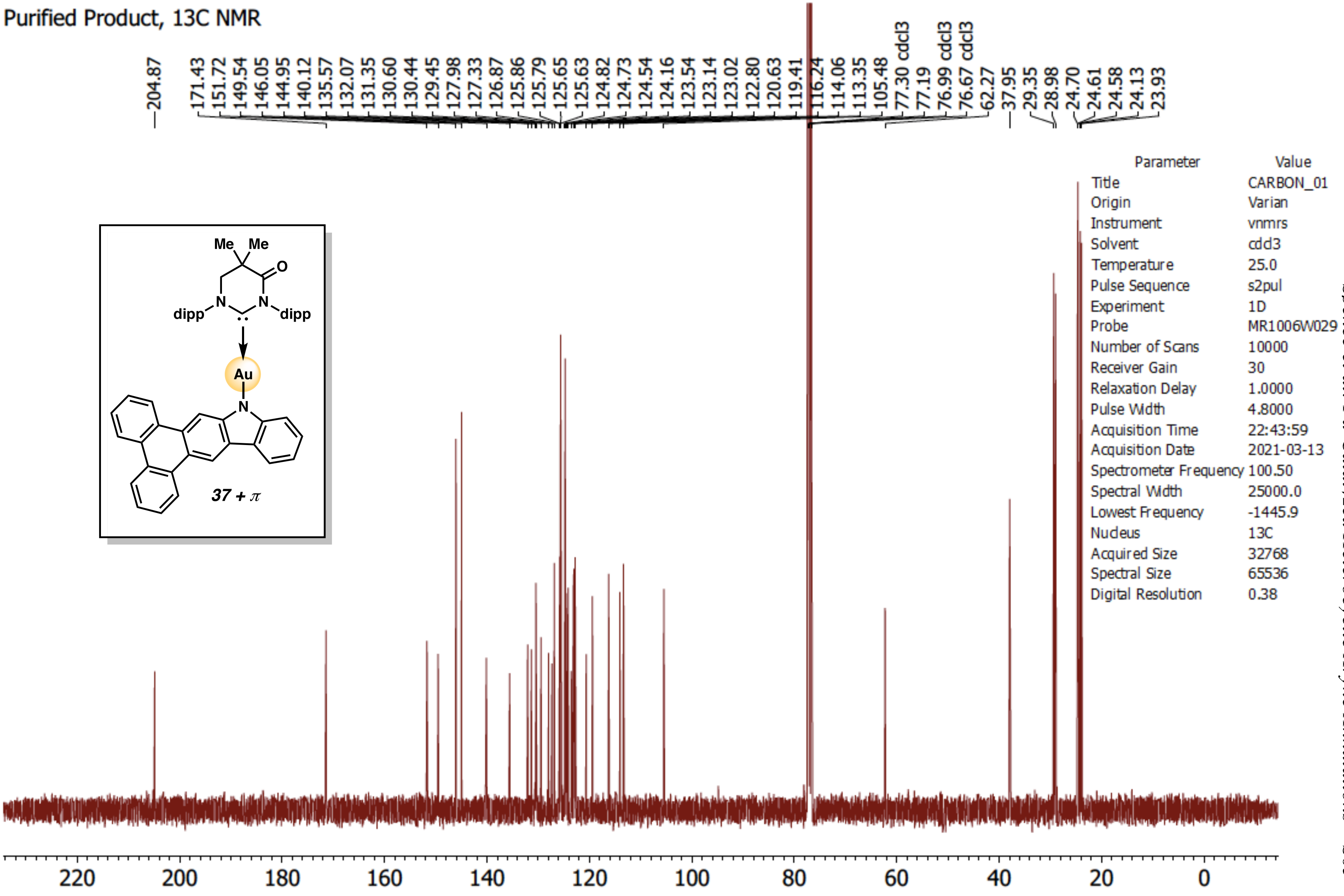

Purified Product, 13C NMR

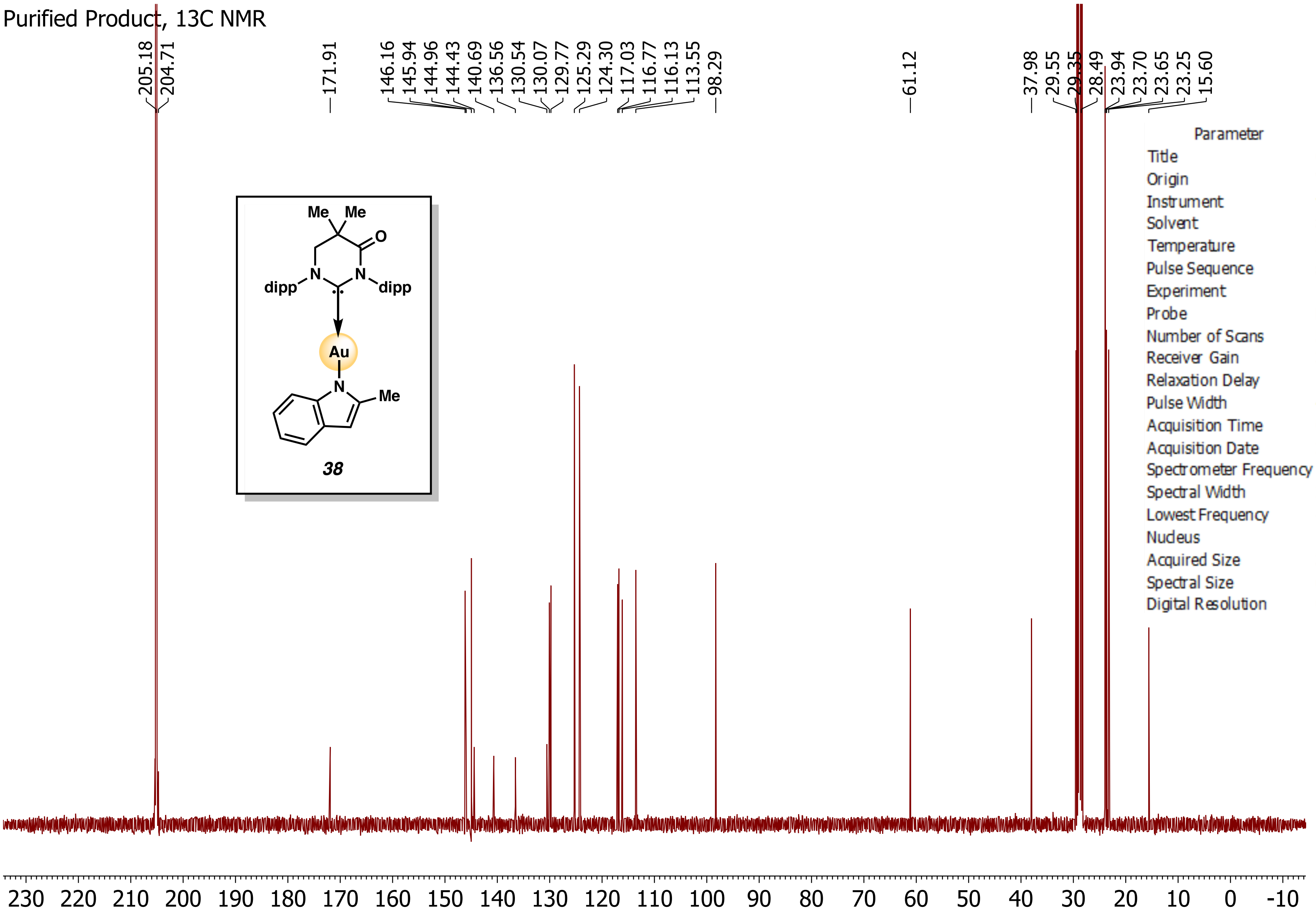

Parameter	Value
Title	CARBON_01
Origin	Varian
Instrument	vnmrs
Solvent	acetone
Temperature	25.0
Pulse Sequence	s2pul
Experiment	1D
Probe	MR1006W029
Number of Scans	5000
Receiver Gain	30
Relaxation Delay	1.0000
Pulse Width	4.8000
Acquisition Time	02:11:47
Acquisition Date	2021-02-14
Spectrometer Frequency	100.50
Spectral Width	25000.0
Lowest Frequency	-1445.9
Nucleus	13C
Acquired Size	32768
Spectral Size	65536
Digital Resolution	0.38

Purified Product, 13C NMR

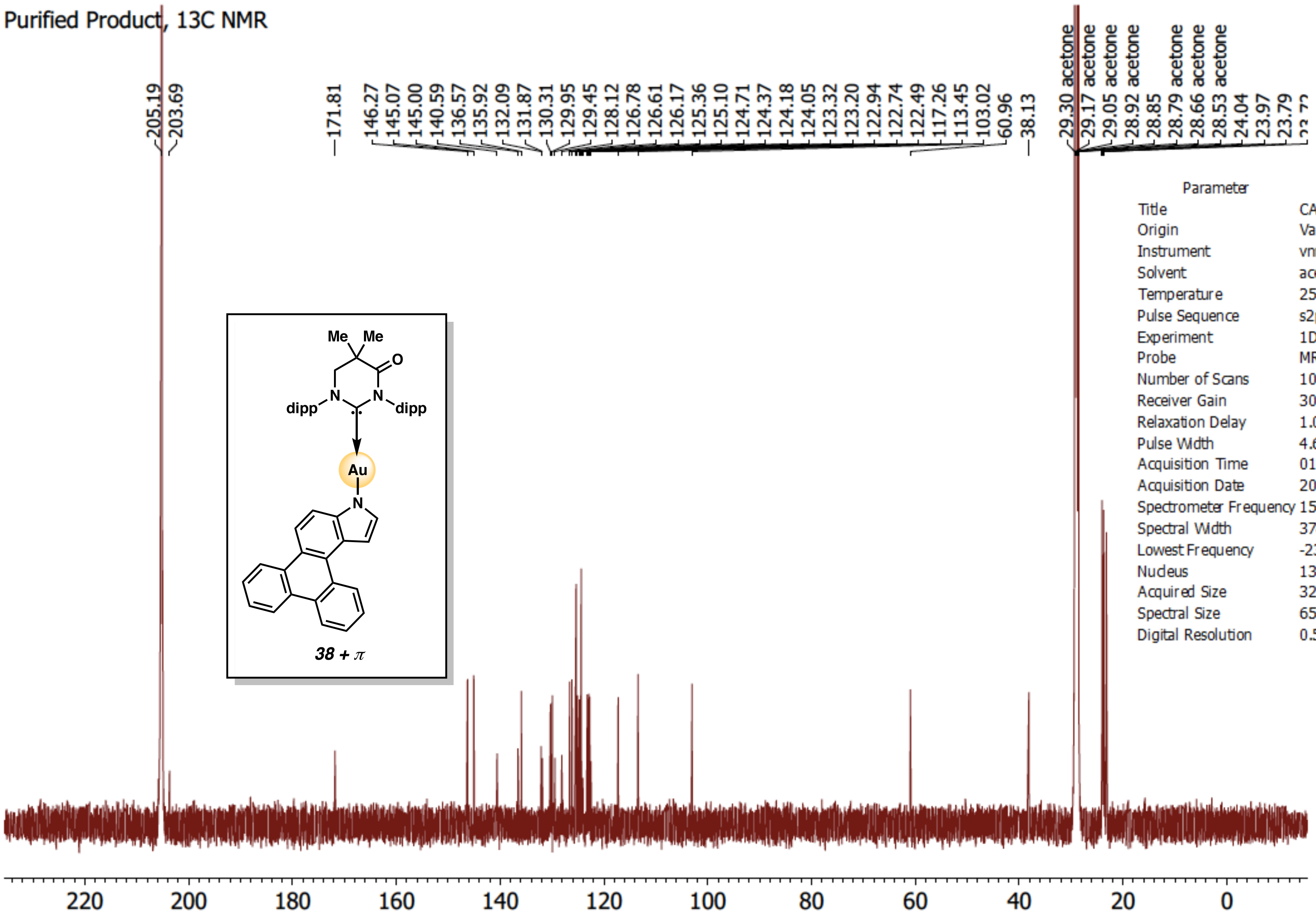

38 + π

Parameter	Value
Title	CARBON_01
Origin	Varian
Instrument	vnmrs
Solvent	acetone
Temperature	25.0
Pulse Sequence	s2pul
Experiment	1D
Probe	MR0808W005
Number of Scans	10000
Receiver Gain	30
Relaxation Delay	1.0000
Pulse Width	4.6000
Acquisition Time	01:07:53
Acquisition Date	2021-03-16
Spectrometer Frequency	150.84
Spectral Width	37878.8
Lowest Frequency	-2349.1
Nucleus	13C
Acquired Size	32768
Spectral Size	65536
Digital Resolution	0.58

NOESY Spectra

NOESY

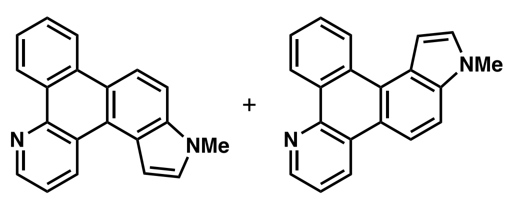

19 **44**

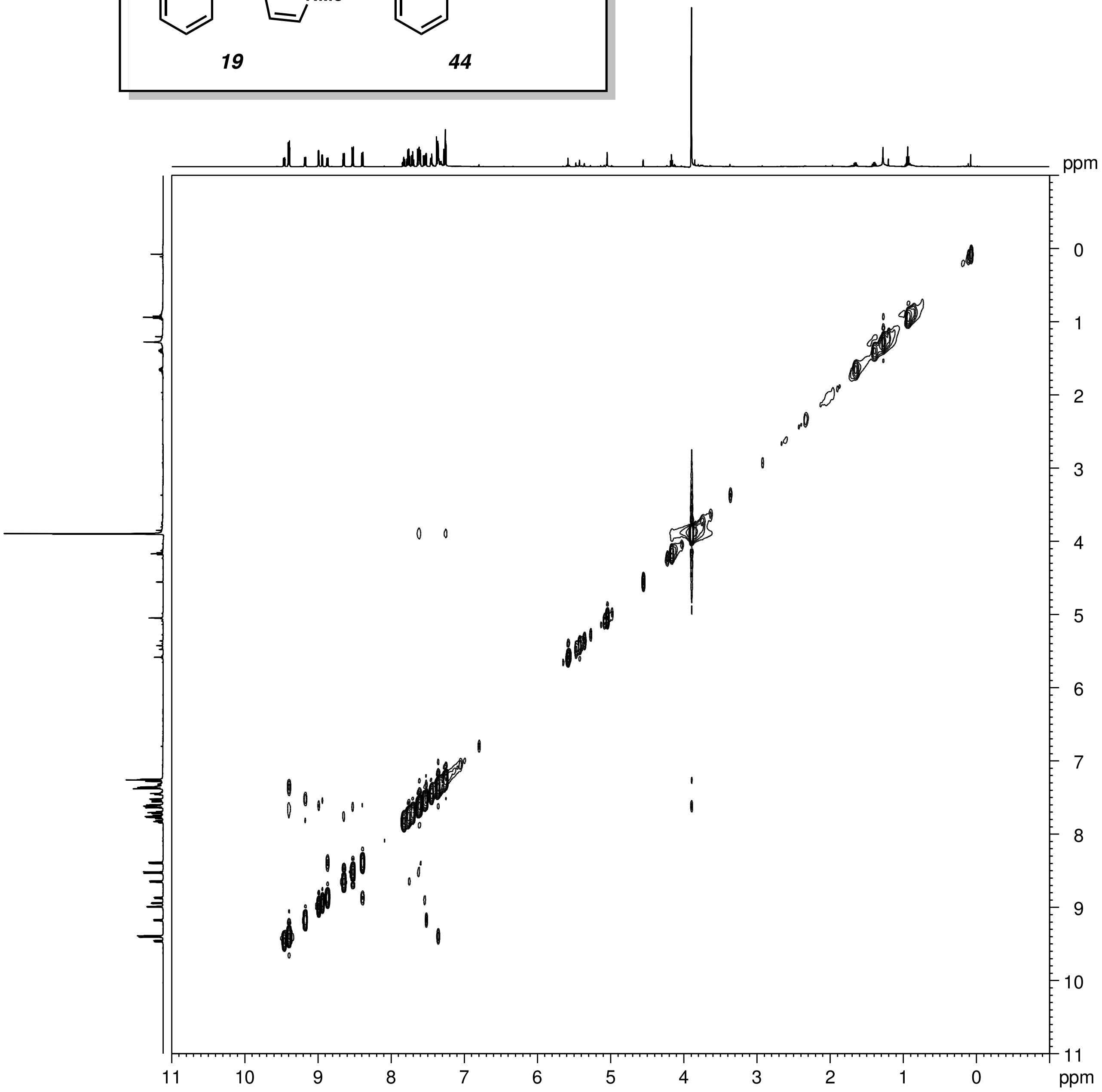

Current Data Parameters
NAME JVC-2018-097
EXPNO 6
PROCNO 1

F2 - Acquisition Parameters
Date_ 20180719
Time 11.16 h
INSTRUM av500
PROBHD Z119248_0002 (
PULPROG noesygpph
TD 2048
SOLVENT CDCl3
NS 6
DS 8
SWH 10000.000 Hz
FIDRES 9.765625 Hz
AQ 0.1024000 sec
RG 12.14
DW 50.000 usec
DE 10.00 usec
TE 298.0 K
D0 0.00003727 sec
D1 2.00000000 sec
D8 0.75000000 sec
D16 0.00020000 sec
IN0 0.00010000 sec
TDav 1
SFO1 500.1330008 MHz
NUC1 1H
P1 10.00 usec
P2 20.00 usec
PLW1 13.50000000 W
GPNAM[1] SINE.100
GPZ1 40.00 %
P16 1000.00 usec

F1 - Acquisition parameters
TD 256
SFO1 500.133 MHz
FIDRES 78.125000 Hz
SW 19.995 ppm
FnMODE States-TPPI

F2 - Processing parameters
SI 2048
SF 500.1300124 MHz
WDW QSINE
SSB 2
LB 0 Hz
GB 0
PC 1.40

F1 - Processing parameters
SI 2048
MC2 States-TPPI
SF 500.1300128 MHz
WDW QSINE
SSB 2
LB 0 Hz
GB 0

NOESY

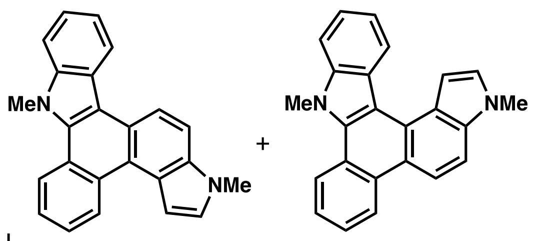

22 50

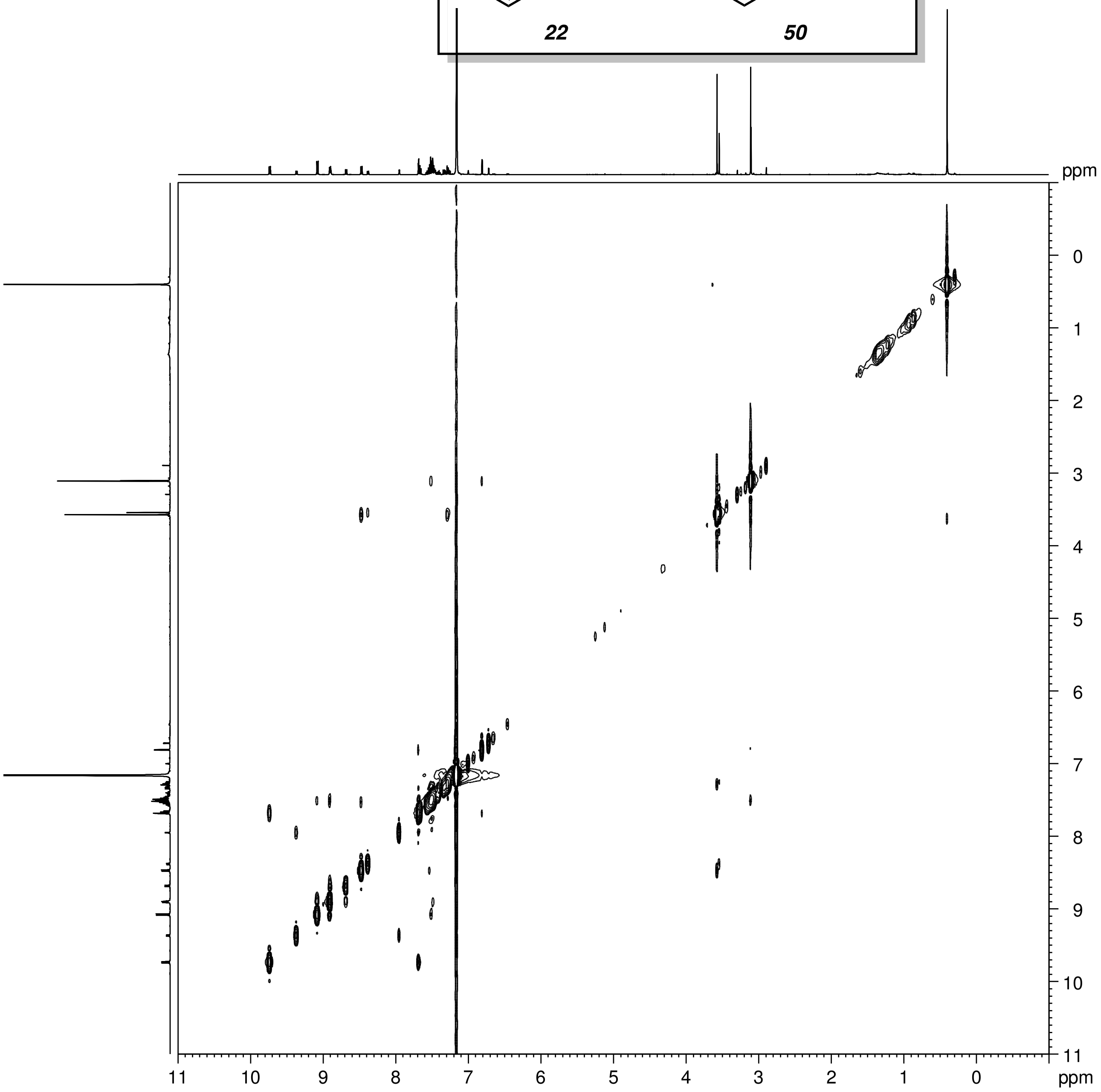

Current Data Parameters
NAME JVC-2018-107-3-2D
EXPNO 3
PROCNO 1

F2 - Acquisition Parameters
Date_ 20180819
Time 21.40 h
INSTRUM av500
PROBHD Z119248_0002 (
PULPROG noesygpph
TD 2048
SOLVENT C6D6
NS 6
DS 8
SWH 10000.000 Hz
FIDRES 9.765625 Hz
AQ 0.1024000 sec
RG 12.14
DW 50.000 usec
DE 10.00 usec
TE 298.0 K
D0 0.00003727 sec
D1 2.00000000 sec
D8 0.75000000 sec
D16 0.00020000 sec
IN0 0.00010000 sec
TDav 1
SFO1 500.1330008 MHz
NUC1 1H
P1 10.00 usec
P2 20.00 usec
PLW1 13.50000000 W
GPNAM[1] SINE.100
GPZ1 40.00 %
P16 1000.00 usec

F1 - Acquisition parameters
TD 256
SFO1 500.133 MHz
FIDRES 78.125000 Hz
SW 19.995 ppm
FnMODE States-TPPI

F2 - Processing parameters
SI 2048
SF 500.1299963 MHz
WDW QSINE
SSB 2
LB 0 Hz
GB 0
PC 1.40

F1 - Processing parameters
SI 2048
MC2 States-TPPI
SF 500.1299969 MHz
WDW QSINE
SSB 2
LB 0 Hz
GB 0

Supplementary References

(1) Zhang, Q.-W.; An, K.; Liu, L.-C.; Guo, S.; Jiang, C.; Guo, H.; He, W. *Angew. Chem., Int. Ed.* **2016**, *55*, 6319–6323.

(2) Wang, T.-F.; Lin, C.-L.; Chen, C.-N.; Wang, T.-C. *J. Chin. Chem. Soc.* **2007**, *54*, 811–816.

(3) Wu, D.; Chen, L.; Ma, S.; Luo, H.; Cao, J.; Chen, R.; Duan, Z.; Mathey, F. *Org. Lett.* **2018**, *20*, 4103−4106.

(4) Panteleev, J.; Geyer, K.; Aguilar-Aguilar, A.; Wang, L.; Lautens, M. *Org. Lett.* **2010**, *12*, 5092–5095.

(5) Wong, S. M.; Yuen, O. Y.; Choy, P. Y.; So, C. M.; Kwong, F. Y. *Org. Synth.* **2016**, *93*, 14–28.

(6) Bronner, S. M.; Bahnck, K. B.; Garg, N. K. *Org. Lett.* **2009**, *11*, 1007–1010.

(7) Im, G.-Y. J.; Bronner, S. M.; Goetz, A. E.; Paton, R. S.; Cheong, P. H.-Y.; Houk, K. N.; Garg, N. K. *J. Am. Chem. Soc.* **2010**, *132*, 17933–17944.

(8) Goetz, A. E.; Silberstein, A. L.; Corsello, M. A.; Garg, N. K. *J. Am. Chem. Soc.* **2014**, *136*, 3036–3099.

(9) Hamze, R.; Idris, M.; Ravinson, D. S. M.; Jung, M. C.; Haiges, R.; Djurovich, P. I.; Thompson. M. E. *Front. Chem.* **2020**, *8*, 401.

(10) Shi, S.; Jung, M. C.; Coburn, C.; Tadle, A.; Sylvinson, D. M. R.; Djurovich, P. I.; Forrest, S. R.; Thompson, M. E. *J. Am. Chem. Soc.* **2019**, *141*, 3576–3588.

(11) Shao, Y. H.; Gan, Z. T.; Epifanovsky, E.; Gilbert, A. T. B.; Wormit, M.; Kussmann, J.; Lange, A. W.; Behn, A.; Deng, J.; Feng, X. T.; Ghosh, D.; Goldey, M.; Horn, P. R.; Jacobson, L. D.; Kaliman, I.; Khaliullin, R. Z.; Kus, T.; Landau, A.; Liu, J.; Proynov, E. I.; Rhee, Y. M.; Richard, R. M.; Rohrdanz, M. A.; Steele, R. P.; Sundstrom, E. J.; Woodcock, H. L.;

Zimmerman, P. M.; Zuev, D.; Albrecht, B.; Alguire, E.; Austin, B.; Beran, G. J. O.; Bernard, Y. A.; Berquist, E.; Brandhorst, K.; Bravaya, K. B.; Brown, S. T.; Casanova, D.; Chang, C. M.; Chen, Y. Q.; Chien, S. H.; Closser, K. D.; Crittenden, D. L.; Diedenhofen, M.; DiStasio, R. A.; Do, H.; Dutoi, A. D.; Edgar, R. G.; Fatehi, S.; Fusti-Molnar, L.; Ghysels, A.; Golubeva-Zadorozhnaya, A.; Gomes, J.; Hanson-Heine, M. W. D.; Harbach, P. H. P.; Hauser, A. W.; Hohenstein, E. G.; Holden, Z. C.; Jagau, T. C.; Ji, H. J.; Kaduk, B.; Khistyaev, K.; Kim, J.; King, R. A.; Klunzinger, P.; Kosenkov, D.; Kowalczyk, T.; Krauter, C. M.; Lao, K. U.; Laurent, A. D.; Lawler, K. V.; Levchenko, S. V.; Lin, C. Y.; Liu, F.; Livshits, E.; Lochan, R. C.; Luenser, A.; Manohar, P.; Manzer, S. F.; Mao, S. P.; Mardirossian, N.; Marenich, A. V.; Maurer, S. A.; Mayhall, N. J.; Neuscamman, E.; Oana, C. M.; Olivares-Amaya, R.; O'Neill, D. P.; Parkhill, J. A.; Perrine, T. M.; Peverati, R.; Prociuk, A.; Rehn, D. R.; Rosta, E.; Russ, N. J.; Sharada, S. M.; Sharma, S.; Small, D. W.; Sodt, A.; Stein, T.; Stuck, D.; Su, Y. C.; Thom, A. J. W.; Tsuchimochi, T.; Vanovschi, V.; Vogt, L.; Vydrov, O.; Wang, T.; Watson, M. A.; Wenzel, J.; White, A.; Williams, C. F.; Yang, J.; Yeganeh, S.; Yost, S. R.; You, Z. Q.; Zhang, I. Y.; Zhang, X.; Zhao, Y.; Brooks, B. R.; Chan, G. K. L.; Chipman, D. M.; Cramer, C. J.; Goddard, W. A.; Gordon, M. S.; Hehre, W. J.; Klamt, A.; Schaefer, H. F.; Schmidt, M. W.; Sherrill, C. D.; Truhlar, D. G.; Warshel, A.; Xu, X.; Aspuru-Guzik, A.; Baer, R.; Bell, A. T.; Besley, N. A.; Chai, J. D.; Dreuw, A.; Dunietz, B. D.; Furlani, T. R.; Gwaltney, S. R.; Hsu, C. P.; Jung, Y. S.; Kong, J.; Lambrecht, D. S.; Liang, W. Z.; Ochsenfeld, C.; Rassolov, V. A.; Slipchenko, L. V.; Subotnik, J. E.; Van Voorhis, T.; Herbert, J. M.; Krylov, A. I.; Gill, P. M. W.; Head-Gordon, M. *Mol. Phys.* **2015,** *113*, 184–215.
